# Dissecting the neurocomputational bases of patch-switching

**DOI:** 10.1093/cercor/bhad088

**Published:** 2023-03-16

**Authors:** George Zacharopoulos, Greg Maio, David E J Linden

**Affiliations:** Cardiff University Brain Research Imaging Centre (CUBRIC), School of Psychology, Cardiff University, Cardiff CF24 4HQ, United Kingdom; School of Psychology, Faculty of Medicine, Health and Life Sciences, Swansea University, Swansea SA28PP, United Kingdom; Department of Psychology, University of Bath, Bath, BA2 7AY, United Kingdom; Cardiff University Brain Research Imaging Centre (CUBRIC), School of Psychology, Cardiff University, Cardiff CF24 4HQ, United Kingdom; Department of Psychiatry and Neuropsychology, School of Mental Health and Neuroscience, Maastricht University Medical Center, Universiteitssingel 40, 6229 ER Maastricht, The Netherlands

**Keywords:** patch-switching, fMRI, individual differences, learning

## Abstract

The survival and well-being of humans require solving the *patch-switching* problem: we must decide when to stop collecting rewards in a current patch and travel somewhere else where gains may be higher. Previous studies suggested that frontal regions are underpinned by several processes in the context of foraging decisions such as tracking task difficulty, and/or the value of exploring the environment. To dissociate between these processes, participants completed an fMRI patch-switching learning task inspired by behavioral ecology. By analyzing >11,000 trials collected across 21 participants, we found that the activation in the cingulate cortex was closely related to several patch-switching-related variables including the decision to leave the current patch, the encounter of a new patch, the harvest value, and the relative forage value. Learning-induced changes in the patch-switching threshold were tracked by activity within frontoparietal regions including the superior frontal gyrus and angular gyrus. Our findings suggest that frontoparietal regions shape patch-switching learning apart from encoding classical non-learning foraging processes. These findings provide a novel neurobiological understanding of how learning emerges neurocomputationally shaping patch-switching behavior with implications in real-life choices such as job selection and pave the way for future studies to probe the causal role of these neurobiological mechanisms.

## Introduction

In everyday life, we must frequently decide whether to stay at the current location (henceforth; patch) or to move to a new one, such as leaving our current job to move to a new one. Indeed, humans engage in patch-switching decisions for a wide range of purposes including survival/reproduction (e.g. food, potential mates), education purposes (e.g. which books to read), human social connection (e.g. which social groups to join), and entertainment (e.g. music/movies/leisure activities). Furthermore, humans are often faced with patch-switching problems not only for ourselves but also for others such as family or community members (Zacharopoulos et al. 2018). Given the ubiquitous nature and impact of such decisions in shaping our survival and well-being, it is not surprising that research across multiple disciplines has recently turned to examine the neurocomputational mechanisms underlying patch-switching (Kolling et al. 2012, 2016a, 2016b; Shenhav et al. 2014, 2016a, 2016b; Constantino and Daw 2015).

There are several parameters encoded by the brain when deciding whether to leave a current location or move to a new one including the patch-switching decision threshold, the harvest value, the travel value, and other parameters, which apply in a changing environment such as the depletion rate and the travel time (for details, see Materials and Methods). For example, activity within the monkey dorsal anterior cingulate cortex (dACC) is positively related to the decision to leave the patch, and this activity increases with greater travel time to the next patch, suggesting that dACC activity reflects a patch-switching decision threshold (Hayden et al. 2011; Hall-McMaster and Luyckx 2019). In the human brain, activity within the dACC was positively related to the value of searching for alternative options (Kolling et al. 2012) although it has also been proposed that dACC encodes task difficulty instead (Shenhav et al. 2014). Still, subsequent reports showed that decision difficulty and search value in the original foraging study (Kolling et al. 2012) shared only 2% of their variance (Kolling et al. 2016a). A subsequent investigation identified a negative correlation between search value and decision difficulty and found that dACC activity still tracked search value (Kolling et al. 2018) and in the macaque, it was shown that dACC activity tracks search value (Stoll et al. 2016).

Moreover, some accounts emphasize that vmPFC activity is positively related to the chosen option's reward magnitude in binary economic choices as well as encoding the value of the default (non-switch) option during stay-switch foraging style decisions (Kolling et al. 2012), and other accounts emphasize that activity within the dACC and vmPFC correlates with the relative value of searching for alternatives over engaging with current options (Shenhav et al. 2016b).

**Fig. 1:**
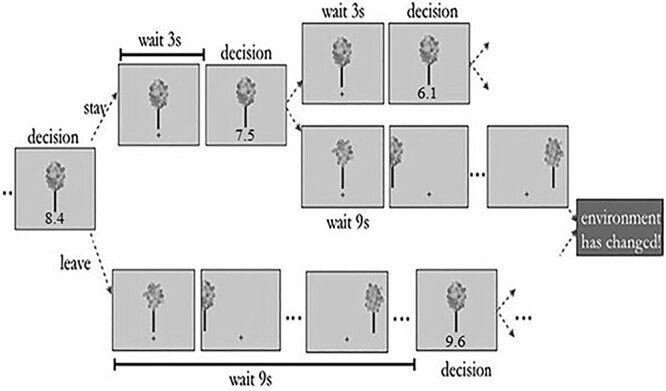
Graphical depiction of the experimental task. On each trial, participants were presented with a cartoonish picture of an apple tree (patch) below which was displayed the harvest value (e.g. 8.4 apples), and participants indicated (within 1.5 s) whether to harvest the tree for apples and incur a short harvest delay (~3 s), or move to a new tree and incur a longer travel delay (~6 or ~9 s). Harvests earned apples, albeit at an exponentially decelerating rate. Choosing to harvest the tree entailed (i) receiving the harvest value (e.g. 8.4) and (ii) experiencing the harvest delay (~3 s) until the tree was ready to be harvested again but in the next trial, the harvest value of the tree was smaller (e.g. 7.5). Choosing to switch the tree entailed (i) not receiving any reward and (ii) experiencing a travel delay (~6 or ~9 s depending on the environment, see Materials and Methods for details) to travel to a new tree with potentially higher starting harvest value (e.g. 9.6) than the harvest value of the last tree at exit (e.g. 8.4) (modified from Constantino and Daw 2015).

Overall, it is currently an open question whether dACC activity indicates closer proximity to a lower patch-switching threshold (Hayden et al. 2011), a higher search value (Kolling et al. 2012, 2016a, 2016b), or task difficulty/cognitive control (Shenhav et al. 2014, 2016a), and whether vmPFC has a role only in classical binary economic choices or whether its role extends to (personal and/or social) patch-switching decisions. To interrogate these possibilities, we asked participants to perform a novel fMRI patch-switching learning task (Fig. 1), which allowed us to decouple the neurocomputational basis of several patch-switching latent variables including the patch-switching threshold, and thus discern the specific role of distinct brain networks in tracking these variables shaping personal and social patch-switching behavior (for details on the calculation of patch-switching latent variables, see Materials and Methods).

During the fMRI patch-switching learning task (Fig. 1), participants were shown an image of an apple tree (patch) and had to decide whether to harvest it for apples and incur a short harvest delay (and subsequent diminishing returns), or move to a new tree (patch) and incur a longer delay because of travel between the initial and new tree. In other words, participants were faced with a patch-switching problem where they experienced an option with diminishing returns and must decide when it was best to leave that option. Importantly, the time they spent in this virtual environment was fixed; therefore, participants had to maximize reward in relation to time. Crucially, the properties of the environment were not static but changed frequently (from run to run), thus, in order to achieve optimal behavior and maximize reward, participants were expected to constantly keep track of the environment properties and update the corresponding patch-switching latent variables. Moreover, and as alluded to earlier, many patch-switching decisions are social decisions (e.g. partner choice, joining/leaving a social group), and people often have to solve the patch-switching problem not only for themselves but also for others. How then do we solve the patch-switching problem when it concerns others? Does the human brain perform patch-switching computations similarly or distinctly when we forage for others compared with when we merely forage for ourselves? To answer these questions participants during the scanning session performed personal patch-switching (the returns were given to the participant) as well as social patch-switching (the returns were given to a charity). This foraging task allowed the quantification of variables that track learning-induced processes such as patch-switching threshold, depletion rate learning, and travel time learning, as well as the quantification of variables that track non-learning foraging processes such as the binary decision (i.e. stay/harvest vs switch/travel), task difficulty/reaction time, harvest value, new patch, and relative forage value (for details, see Materials and Methods).

We started addressing these questions in a previous study where participants made foraging decisions for themselves and a charity of their choice (Zacharopoulos et al. 2018). We found that individuals who possessed a stronger self-focused value orientation obtained more rewards when they foraged for themselves rather than for charity, and this effect was associated with activity in the dACC. The present study aimed to expand on the previous one by examining the neurocomputational bases of a task that involves a wide range of unique computations and learning variables that we had been able to model in our original study. For example, it is currently unknown how the human brain computes and tracks changing environmental properties that shape foraging behavior, such as travel time, and the depletion rate when we forage for others compared with when we merely forage for ourselves. Apart from our little understanding of how these changing environmental properties (which are explicitly set by the experimenter), our study also examined the neurocomputational basis of latent psychological parameters derived from the person’s behavior, such as the patch-switching threshold, which vary considerably between participants.

Therefore, our aims were: (i) to decouple the neurocomputational basis of several patch-switching latent variables, and thus discern the specific role of certain brain networks in tracking these variables, and (ii) to discern the extent to which these computations are differentially modulated by personal vs social patch-switching decisions. (iii) A secondary aim of the study was to discern how individual differences in human value orientation, assessed via a questionnaire outside the scanner (see Materials and Methods for details), predicted patch-switching behavior.

## Materials and methods

### Participants

We recruited 21 healthy participants (mean age: 26.86; the standard deviation of age: 6.3; 5 males, 16 females) by advertising the study online on Cardiff University notice boards. The study was approved by the Ethics Committee of the School of Psychology at Cardiff University. All participants gave written informed consent according to the Declaration of Helsinki and received £15 monetary compensation for taking part in the study plus additional performance-based payment (see below for details).

### Procedure and experimental task

Prior to scanning, all participants underwent an MRI safety screening, were familiarized with the scanning environment, and performed a training session with stimuli similar to those used in the fMRI session. The scanning session comprised 4 functional runs (~16 min each) and the acquisition of a structural image, leading to a total scanning time of ~1.5 h.

Participants were informed at the start of the experiment that the reward (i.e. the number of points/apples) they collected during the scanning session would be converted into real money at the end of the experiment and that the reward obtained during personal patch-switching would be paid to them (on top of the fixed participation payment of £15), whereas the reward obtained during social patch-switching would be given to a charity of their choice. Participants were then asked to select the charity of their choice from a list including the following charities: British Red Cross, Save the Children Fund, Oxfam, The Salvation Army, Cancer Research UK, and Macmillan Cancer Support.

Participants completed a modified version of a virtual patch-switching task used previously (Constantino and Daw 2015; Zacharopoulos et al. 2018; Fig. 1) in a single scanning session. The scanning session consisted of 4 functional runs and each run consisted of 4 blocks (overall 16 blocks: 8 personal patch-switching blocks and 8 social patch-switching blocks). The scanning session consisted of 4 functional runs and each run consisted of 4 blocks (overall 16 blocks: 8 personal patch-switching blocks and 8 social patch-switching blocks). In every run, half of the blocks (i.e. 2 out of 4) were personal patch-switching blocks and the other 2 blocks were social patch-switching blocks. The environment changed from run to run with the first run being short-shallow (ShSh), the second short-steep (ShSt), the third long-steep (LoSt), and the fourth long-shallow (LoSh). For additional information depicting how several parameters changed per participant, run, and block, see Supporting Information 6, which shows that the order of personal/social conditions and the combination of travel time and depletion rate conditions were fixed across participants. For plots presenting how the patch-switching threshold was modulated across the 4 environments for each participant, see Supporting Information 8. At the onset of each block, an introductory screen was briefly presented indicating the reward recipient (e.g. “Self” or “Charity”). Stimuli were presented via a 45° angled mirror positioned above the head coil reflecting the projection of a computer screen. The description of the trial events is explained in the legend of Fig. 1. After the scanning procedure, participants were debriefed, thanked, and paid both for participating (£15) as well as for their performance (i.e. total reward obtained in response to the personal patch-switching, which was ~£10–£15 in most cases).

### Manipulation of environmental parameters defining the quality of the patch-switching environment

Similar to the previous behavioral investigations (Constantino and Daw 2015; Zacharopoulos et al. 2018), we varied the quality of the patch-switching environment by manipulating 2 parameters: (i) depletion rate and (ii) travel time. The depletion rate determines the rate at which earned apples decrease with subsequent harvest decisions at a given tree. It is a multiplicative decay *κ*, such that if a participant harvests 8 apples in the current trial, the number of apples to be offered/harvested in the next trial will be the depletion rate multiplied by 8. By manipulating the depletion rate, we created one environment with fast depletion (steep, where *κ* was ~0.88) and one with a slower depletion (shallow, *κ* was ~0.94). Additionally, we created 2 more types of environments—long (~9 s) and short (~6 s)—by manipulating the travel time, which is the time it takes to travel to a new tree. Based on this procedure, the harvest time (which was jittered around 3 s) or the travel time (which was jittered around 6 or 9 s) served as the inter-trial interval. The manipulation of these 2 variables (i.e. travel time and depletion rate) resulted in the 4 environments that participants visited during the task: LoSh, LoSt , ShSh, and ShSt. In previous studies using a similar paradigm, participants exhibited higher exit thresholds in the short orchards than in the long orchards and in the shallow orchards than in the steep ones (Constantino and Daw 2015; Zacharopoulos et al. 2018). This occurs because “a longer travel delay or a steeper depletion rate reduces the rate at which apples can be earned,” which, in turn, “reduces the opportunity cost of time spent harvesting, which as a consequence leads an ideal forager to harvest a tree down to a lower number of apples” (Constantino and Daw 2015). In other words, participants switched earlier (higher patch-switching threshold) for short (vs long) orchards and shallow (vs steep) orchards (Constantino and Daw 2015; Zacharopoulos et al. 2018), and we replicated this set of findings in the current investigation (see Results section).

Apart from the depletion rate and travel time, all the other environmental parameters remained the same across the 4 environments. Participants experienced one environment per each run and were notified when the environment was about to change (i) by an introductory message (e.g. “You are now entering a new orchard”) and (ii) by a background color change, even though participants were not aware which parameters of the environment were changed (i.e. depletion rate or travel time) or by how much, but had to empirically experience the changes. New trees were drawn from a Gaussian distribution and the environmental richness or opportunity cost of time was varied across the runs, as mentioned, by changing the travel time and/or the reward depletion rate. The initial value of a tree (see Fig. 1 for an example), depletion rate, and richness of the environment were unknown to the participants. The participants aimed to maximize reward (i.e. number of points/apples) for themselves (personal patch-switching) and/or a charity of their choice (social patch-switching), depending on the experimental condition. This was a within-subject design.

### Human values

Participants completed the Schwartz Value Survey (SVS; Schwartz 1992), a 56-item scale that is used to measure human value orientation. Participants were asked to rate the importance of each of the 56 values as a guiding principle in their lives, using a quasi-bipolar 9-point scale ranging from −1 (opposed to my values), 0 (not important), 4 (important) to 7 (of supreme importance). Examples of SVS items are as follows: “Equality: Equal opportunity for all” (Universalism), “Pleasure: Gratification of desires” (Hedonism), and “Obedient: Dutiful meeting obligations” (Conformity). The average score across the 56 items was calculated and subtracted from each of the 56 initial raw scores, before calculating the average of the value scores within each of the 10 value types. The initial raw scores were the original responses of the participant in response to each item recorded using a quasi-bipolar 9-point scale ranging from −1 (opposed to my values), 0 (not important), 4 (important) to 7 (of supreme importance). Schwartz recommends this procedure to help control for superfluous individual variations in rating styles (Schwartz 1992). Following his model, we created the self-focus score, by calculating the average score on self-direction, stimulation, hedonism, achievement, power, and security values. To calculate the social-focus score, we calculated the average score of universalism, benevolence, tradition, conformity, and security values.

### MRI data acquisition

MRI images were acquired with a General Electric 3T scanner equipped with an 8HR Brain parallel head coil for radio frequency transmission/reception. Anatomical high-resolution T1-weighted volume scans (1 mm^3^) were acquired using FSPGR sequence (TR = 7.796 ms; TE = 2.984 ms; voxel size = 1 × 1 × 1 mm, 200 slices). Functional images were acquired with an EPI sequence (TR = 2,000 ms, TE = 30 ms, flip angle = 85 degrees, slice thickness = 3 mm). Each volume consisted of 39 slices obtained in an ascending interleaved order.

### fMRI data preprocessing and analyses

Imaging data were preprocessed in CONN (Whitfield-Gabrieli and Nieto-Castanon 2012) (a toolbox in SPM12) using the default MNI-space direct normalization preprocessing pipeline, which performs several steps including realignment, slice-timing correction, structural segmentation and normalization, functional normalization, and smoothing (smoothing kernel was 8 mm). At the first-level analyses, we ran a single GLM (for an example of the parametric modulators, see Supporting Information 4) that featured 2 main predictors: (i) the event onsets during the personal patch-switching condition and (ii) the event onsets during the social patch-switching condition (convolved with the canonical HRF). For the correlations between the parametric modulators see Supporting Information 11. Each main predictor featured 8 parametric modulators that are clustered into 2 general categories: (i) parametric modulators that are associated with individual behavior (i.e. decision (1), reaction time (2), patch-switching threshold (3)) and (ii) parametric modulators that are associated with the foraging environment (harvest value (4), new patch (5), travel time learning (6), depletion rate learning (7), relative forage value (8)):

(1) Decision*:* this was a binary variable denoting whether participants have chosen to harvest (1) or switch (0) the tree on every trial.
(2) Reaction time: this was defined as the time in seconds that participants took to reach a decision in each trial. This was added as a proxy for task difficulty.
(3) Patch-switching threshold: this was defined as the average between the (i) harvest value of the tree at the exit and the (ii) harvest value during the most recent harvest of the same tree. Importantly, because the patch-switching threshold can only be changed when participants exit a tree, for the trials where the participant chose to harvest the tree, the value of the patch-switching threshold was defined as the most recently changed patch-switching threshold and stayed as such until participants exited the tree. Put simply, if in trials 1, 2, 3 the harvest value was 9, 8, 10, and the participant chose to harvest, exit, and harvest, then the parametric modulator patch-switching threshold would have been (9 + 8)/2 = 8.5 for trial 2 (changed because of exit decision), and stayed at 8.5 for trial 3 (not changed because of harvest decision).
(4) Harvest value: this was defined as the reward (i.e. the number of points/apples) offered on every trial.
(5) New patch: this was a binary variable denoting whether participants encountered a new tree (1) or not (0) on every trial. Although similar, this differs from the first parametric modulator in that (i) it is shifted in time (i.e. if a participant chooses to switch on trial i, the value of the first parametric modulator on trial i will be 0 and the value of this parametric modulator will be 0 but the value of this parametric modulator will be 1 when the new patch emerges, that is on trial i + 1), and (ii) there are instances where a novel patch appears (and thus this parametric modulator is set to 1) not because a participant had chosen to travel in the previous trial, but because of a changing condition (i.e. because the orchard or the recipient changed).
(6) Travel time learning: in trial ith, this was defined as the average between (i) the average of all previous travel times experienced from trial 1 to trial i − 1, and (ii) the travel time on trial i. This is a learning variable because it tracks the accumulated changes in travel time across orchards, whereas participants learn the properties of each orchard.
(7) Depletion rate learning: in trial i, this was defined as the average between (i) the average of all previous depletion rates experienced from trial 1 to trial i − 1, and (ii) the depletion rate in trial i. This is a learning variable because it tracks the accumulated changes in depletion rate across orchards, whereas participants learn the properties of each orchard.
(8) Relative forage value: this was defined as the ratio between the patch-switching threshold on trial i and the harvest value on trial i (i.e. patch-switching threshold/harvest value).

To identify the effects of interest while controlling for the other predictors irrespective of their added order in all GLMs, we set the orthogonalization to 0. To remove variations in signal because of movement artifacts, we additionally included in our GLM the movement parameters calculated during the realignment in the model as parameters of no interest. Moreover, we excluded all runs where there was beyond 2 mm movement in either of the 3 translations (*x*,*y*,*z*).

We created 16 contrasts during the first-level analyses as we examined the effects of each of the 8 parametric modulators (collapsing across personal and social conditions) as well as the corresponding 8 interactions of these parametric modulators with the reward recipient (i.e. personal vs social conditions). We then entered the contrast of parameter estimate images into a second-level group analysis. Examples of contrasts can be seen in Supporting Information 12, where the reader can also find a table featuring the order of the predictors in the design matrix, the name of the predictors, and the predictor type. Each parametric modulator was a separate regressor in the GLM. The interactions were computed as contrasts after the GLM were run. The parametric modulators were not demeaned as can be seen in Supporting Information 4. For control analyses featuring demeaned parametric modulators see Supporting Information 13. The imaging results, which were significant at a cluster-wise *P*_FWE_ < 0.05, were obtained using the SPM toolbox Statistical Non-Parametric Mapping (SnPM, http://warwick.ac.uk/snpm), which uses the GLM to construct statistic images, which are then assessed for significance using a standard nonparametric procedure based on randomization/permutation testing. The cluster-wise *P*_FWE_ that is reported for each significant cluster in the main text and the Supporting Information does not account for the correction across the different contrasts but it is the value generated from SPM for a given contrast. We also provide the FDR corrected *P*-value for each cluster after controlling for all significant clusters. In addition, we created pseudo-t maps that are computed by smoothing the variance before creating a *t*-ratio, as this approach eliminates the roughness of the activation cluster and effectively increases the degrees of freedom, increasing statistical power (SnPM, http://warwick.ac.uk/snpm). Our approach is consistent with current guidelines on the reporting of whole-brain MRI data (Roiser et al. 2016).

### Statistical analyses

For the behavioral analyses, the dependent variables were (i) the total reward obtained (i.e. the sum of numbers/apples) and (ii) the patch-switching threshold, which was calculated as the mean patch-switching threshold (see above) only based on the trials when participants chose to travel to a new tree. To assess the behavioral effects of our manipulation, we employed a 2 (*Travel time*: Short vs Long) ^*^2 (*Depletion rate*: Steep vs Shallow) ^*^2 (*Source*: Personal vs Social) repeated measures ANOVA predicting patch-switching threshold or total reward. To assess the extent to which the human value orientation predicted total reward, we employed bivariate correlations between the self-focus human value orientation (see above for details) and total reward obtained during the personal patch-switching condition. To assess the extent to which the human value orientation predicted the patch-switching threshold, we employed bivariate correlations between the self-focus human value orientation (see above for details) and the patch-switching threshold in the 4 orchards separately and we corrected for the 12 comparisons (denoted by *P*_FDR_). For completeness, we additionally present the analogous results using the social-focus human value orientation. Of note, we excluded the first 4 participants from the behavioral analyses because they only experienced one travel time (e.g. Long) across the experiment because of a technical error. The first 4 participants were pilot participants, which were included in the imaging analyses to increase the statistical power to detect the effects as all the conditions and parametric modulators were present in these cases.

## Results

### Behavioral results

**Fig. 2:**
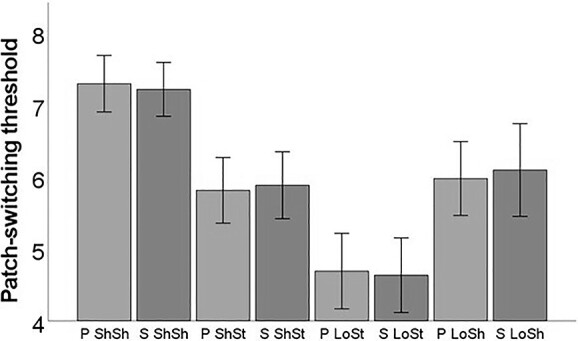
The mean patch-switching threshold in each of the 8 experimental conditions (P: personal patch-switching condition and S: social patch-switching condition). The error bars represent 95% confidence intervals. People switched earlier (higher patch-switching threshold) for short (vs long) environments and shallow (vs steep) environments replicating previous work (Constantino and Daw 2015; Zacharopoulos et al. 2018).

As a first step, we assessed the effect of travel time and depletion rate on the patch-switching threshold (for details of the full statistical model see Materials and Methods) and we fully replicated the results of the previous studies by others and us (Constantino and Daw 2015; Zacharopoulos et al. 2018). Specifically (see also Fig. 2), travel time (*F*(1, 16) = 32.73, *P* < 0.001) and depletion rate (*F*(1, 16) = 35.97, *P* < 0.001) were statistically significant in predicting patch-switching threshold but neither source (*F*(1, 16) = 0.036, *P* = 0.852) nor any of the interactions were significant (source^*^travel time: *F*(1, 16) = 0.096, *P* = 0.761, source^*^depletion rate: *F*(1, 16) = 0.010, *P* = 0.921, travel time^*^depletion rate: *F*(1, 16) = 0.017, *P* = 0.899, source^*^travel time^*^depletion rate: *F*(1, 16) = 1.598, *P* = 0.224). The same pattern of results emerged when the dependent variable was the total reward, where the main effects of travel time (*F*(1, 16) = 62.45, *P* < 0.001) and depletion rate (*F*(1, 16) = 131.17, *P* < 0.001) were statistically significant but neither source (*F*(1, 16) = 0.001, *P* = 0.973) nor any of the interactions were significant (source^*^travel time: *F*(1, 16) = 1.54, *P* = 0.232, source^*^depletion rate: *F*(1, 16) = 0.290, *P* = 0.598, travel time^*^depletion rate: *F*(1, 16) = 0.274, *P* = 0.608, source^*^travel time^*^depletion rate: *F*(1, 16) = 0.278, *P* = 0.605). For additional information from the ANOVA models including estimates and post hoc comparisons, see Supporting Information 7. Moreover, we found that self-focus was positively related (*r*(15) = 0.514, *P* = 0.035), whereas social focus was negatively related, albeit nonsignificantly (*r*(15) = −0.364, *P* = 0.151), to the number of points obtained during personal patch-switching, consistent with our previous findings (Zacharopoulos et al. 2018).

**Fig. 3:**
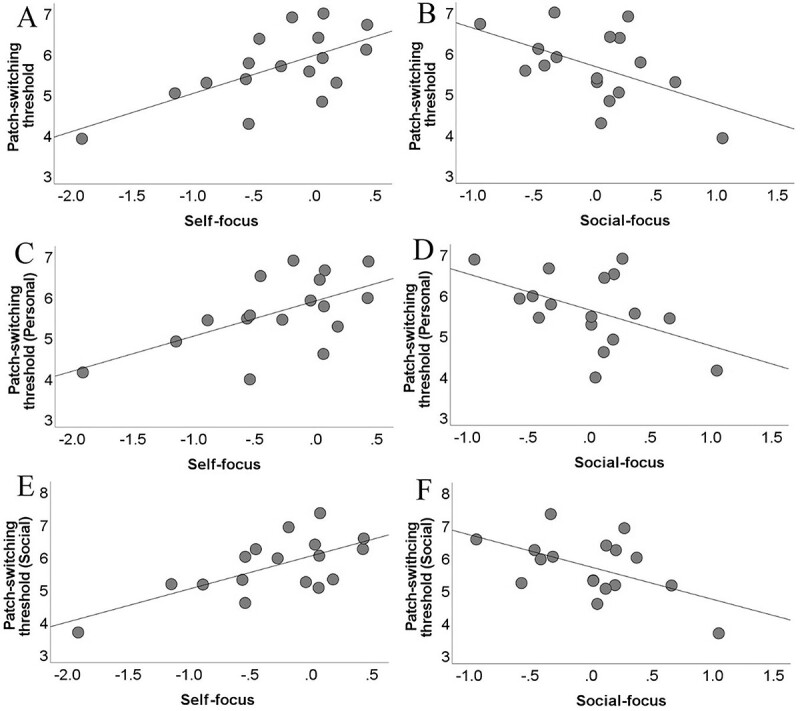
Scatterplots depicting positive (self-focus) or negative (social focus) associations between the human value orientation displayed on the *x*-axis (self-focus or social focus) and path-switching threshold (*y*-axis) in the ShSt environment during all of the trials A, B), only the personal condition C, D) or only the social condition E, F).

We additionally assessed the effect of human value orientation in predicting the patch-switching threshold in the 4 environments. We found that this was the case only for one of the 4 environments: the ShSt. Specifically, self-focus was consistently positively associated with the social (Fig. 3C, *r*(15) *= 0.673*, *P* = 0.003, *P*_FDR_ = 0.037), and average (Fig. 3E, *r*(15) = 0.646*, P* = 0.005, *P*_FDR_ = 0.031) mean patch-switching threshold, and showed a trend with the personal (Fig. 3A, *r*(15) *=* 0.585, *P* = 0.014, *P*_FDR_ = 0.05). Consistent with this, social focus was consistently negatively associated with the social (Fig. 3D, *r*(15) *=* −0.526, *P* = 0.030) and average (Fig. 3F, *r*(15) *=* −0.516, *P* = 0.034) mean patch-switching threshold and showed a trend with the personal (Fig. 3B, *r*(15) *=* −0.479*, P* = 0.05). Regarding ShSh, self-focus was not significantly associated to the personal (*r*(15) = 0.132*, P* = 0.613), social (*r*(15) = −0.048*, P* = 0.855), and average (*r*(15) = 0.047*, P* = 0.858) mean patch-switching threshold. Social focus was not significantly associated to the personal (*r*(15) *=* −0.186*, P* = 0.475), social (*r*(15) = 0.029*, P* = 0.911), and average (*r*(15) = −0.086*, P* = 0.744) mean patch-switching threshold.

Regarding LoSt, self-focus was not significantly associated with the personal (*r*(15) = 0.191*, P* = 0.463), social (*r*(15) = 0.355, *P* = 0.162), and average (*r*(15) = 0.281, *P* = 0.274) mean patch-switching threshold. Social focus was not significantly associated to the personal (*r*(15) *=* −0.218, *P* = 0.401), social (*r*(15) = −0.282, *P* = 0.273), and average (*r*(15) = −0.258, *P* = 0.318) mean patch-switching threshold.

Regarding LoSh, self-focus was not significantly associated with the personal (*r*(15) = −0.159, *P* = 0.542), social (*r*(15) *=* −0.231, *P* = 0.371), and average (*r*(15) *=* −0.207, *P* = 0.425) mean patch-switching threshold. Social focus was not significantly associated to the personal (*r*(15) = 0.065*, P* = 0.805), social (*r*(15) *=* 0.110*, P* = 0.674), and average (*r*(15) = 0.094*, P* = 0.721) mean patch-switching threshold.

### Imaging results

After establishing that the experimental manipulation induced robust behavioral effects, and discerning how the patch-switching threshold and total reward were predicted by the human value orientation, we assessed the neurobiological computations of the 8 parametric modulators (and for a full description of the significant clusters, see Supporting Information 1), which are clustered into 2 general categories: (i) parametric modulators that are associated with individual behavior (i.e. decision (1), reaction time (2), patch-switching threshold (3)) and (ii) parametric modulators that are associated with the foraging environment (harvest value (4), new patch (5), travel time learning (6), depletion rate learning (7), relative forage value (8)). Here we present the results from 4 parametric modulators because 2 of the parametric modulators were essentially confounding variables we wanted to account for (i.e. new patch and reaction time) and 2 parametric modulators of interest (i.e. travel time learning and depletion rate learning) did not yield significant results.

#### Parametric modulators that are associated with individual behavior

##### Patch-switching threshold

**Fig. 4:**
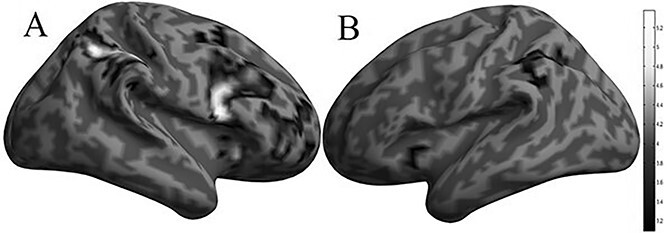
Imaging results depicting the effect of the parametric modulator patch-switching threshold on bilateral (A): right: angular gyrus, superior frontal gyrus, precentral gyrus; B): left: angular gyrus) frontoparietal regions (negative association).

The patch-switching threshold was tracked by activity within frontoparietal regions (for a full description of the significant clusters, see Supporting Information 1) including the bilateral angular gyrus (right: *P*_FWE_ = 0.003, *k* = 2,399, *x* = 36, *y* = −66, *z* = 46, and left: *P*_FWE_ = 0.008, *k* = 1,251, *x* = −34, *y* = −60, *z* = 40, Fig. 4A-B), the right superior frontal gyrus (*P*_FWE_ = 0.021, *k* = 546, *x* = 4, *y* = 28, *z* = 44, Fig. 4A), and the right precentral gyrus (*P*_FWE_ = 0.001, *k* = 3,813, *x* = 52, *y* = 10, *z* = 18, Fig. 4A). Activity within all of these regions was negatively associated with the patch-switching threshold.

##### Decision

**Fig. 5:**
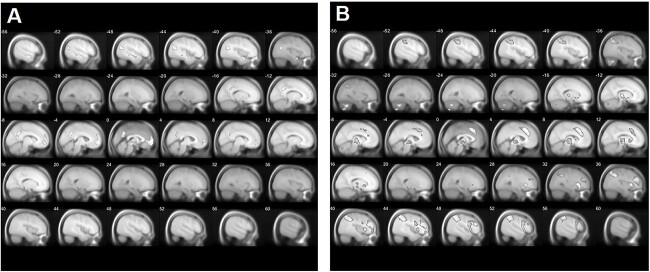
Imaging results (A): positive association and B): negative association) in response to the parametric modulator of decision.

The decision whether to harvest or to leave the current patch was encoded mainly by frontoparietal regions (for additional details on the significant clusters, see Supporting Information 1) where the decision to harvest vs to leave engaged activity within the right superior frontal gyrus (*P*_FWE_ = 0.008, *k* = 1,221, *x* = 0, *y* = 58, *z* = 2, Fig. 5), whereas the decision to leave vs harvest elicited activity within the dACC/paracingulate gyrus (*P*_FWE_ = 0.003, *k* = 1,823, *x* = 4, *y* = 24, *z* = 42, Fig. 5), the right angular gyrus (*P*_FWE_ = 0.002, *k* = 2,130, *x* = 46, *y* = −56, *z* = 52, Fig. 5), and the left supramarginal gyrus (*P*_FWE_ = 0.004, *k* = 1,536, *x* = −46, *y* = −34, *z* = 38, Fig. 5).

#### Parametric modulators that are associated with the foraging environment

##### Harvest value

**Fig. 6:**
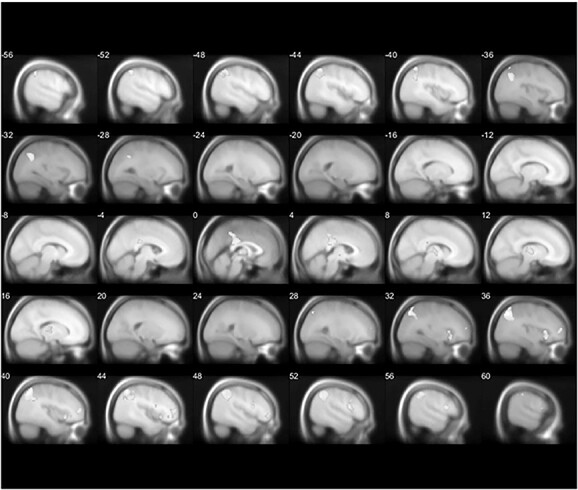
Imaging results in response to the parametric modulator of harvest value.

The parametric modulator harvest value was positively associated with activity within frontoparietal regions (for additional details on the significant clusters, see Supporting Information 1**)**, including the angular gyrus (right: *P*_FWE_ = 0.003, *k* = 1,581, *x* = 36, *y* = −68, *z* = 46, and left: *P*_FWE_ = 0.004, *k* = 896, *x* = −52, *y* = −48, *z* = 48, Fig. 6), the frontal gyrus (right inferior: *P*_FWE_ = 0.036, *k* = 317, *x* = 54, *y* = 12, *z* = 16, and right middle/frontal pole: *P*_FWE_ = 0.033, *k* = 323, *x* = 42, *y* = 50, *z* = 8, Fig. 6), and a region encompassing PCC/dACC (*P*_FWE_ = 0.017, *k* = 511, *x* = 0, *y* = −28, *z* = 26, Fig. 6).

##### Relative forage value

**Fig. 7:**
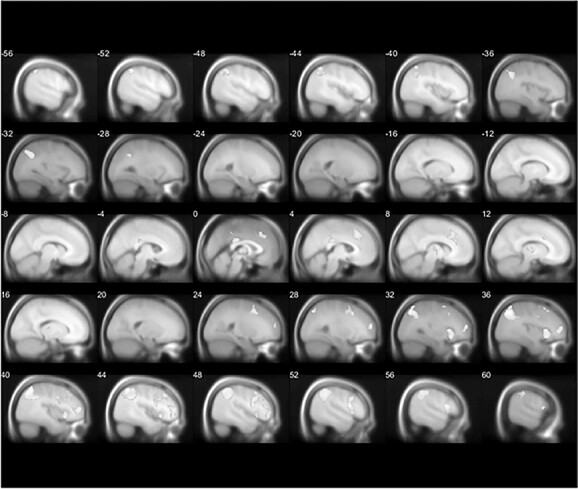
Imaging results in response to the parametric modulator of relative forage value.

The parametric modulator foraging value was positively associated with activity within frontoparietal regions (for additional details on the significant clusters, see Supporting Information 1), including the angular gyrus (right: *P*_FWE_ = 0.001, *k* = 2,103, *x* = 36, *y* = −68, *z* = 46, and left: *P*_FWE_ = 0.003, *k* = 862, *x* = −52, *y* = −48, *z* = 48, Fig. 7), the frontal gyrus (right inferior: *P*_FWE_ = 0.001, *k* = 1,678, *x* = 56, *y* = 14, *z* = 14, right middle/frontal pole: *P*_FWE_ = 0.005, *k* = 699, *x* = 42, *y* = 50, *z* = 8, right superior frontal: *P*_FWE_ = 0.013, *k* = 492, *x* = 4, *y* = 28, *z* = 42, and another right middle region: *P*_FWE_ = 0.03, *k* = 264, *x* = 24, *y* = 10, *z* = 50, Fig. 7), and a region encompassing PCC/dACC (*P*_FWE_ = 0.029, *k* = 253, *x* = 2, *y* = −28, *z* = 26, Fig. 7).

## Discussion

The present fMRI study assessed the neurocomputational basis of patch-switching by focusing on computational metrics such as patch-switching threshold and relative forage value elicited in response to a novel learning paradigm inspired by behavioral ecology. Four main findings emerged from our study: (i) the patch-switching threshold was tracked by activity within frontoparietal regions including the angular and superior frontal gyrus, which possibly inhibits switching propensity, (ii) several frontoparietal regions (angular and frontal gyrus) tracked several patch-switching properties, (iii) the absence of activity differences in these computational metrics between the personal and social conditions, and (iv) the identification that human value orientation predicted patch-switching threshold in a specific foraging environment.

The present investigation primarily focused on the examination of the patch-switching threshold, a computational metric that determines when to leave collecting rewards in a current patch and travel somewhere else. Behaviorally, we consistently replicated the previously documented effects of the experimental manipulation (Constantino and Daw 2015; Zacharopoulos et al. 2018). Namely, participants exhibited a higher patch-switching threshold (they switch earlier) in shallow vs steep environments, and in the short vs long environments, both across personal and social conditions. Apart from replicating these findings, we also revealed a psychological determinant that predicted individual variation in the total reward obtained and patch-switching threshold: the human value orientations of self-focus and social focus. We replicated our previous finding that the personal-focus value score was positively related (*r*(15) = 0.514, *P* = 0.035) to the number of points during personal patch-switching (Zacharopoulos et al. 2018), and we also found that self-focus was positively, and social focus was negatively associated with the patch-switching threshold both during the personal and the social conditions. This identification of a novel determinant of the patch-switching threshold is a clear demonstration of how social psychology research (Schwartz 1992) can help inform behavioral ecology.

Crucially, these consistent patterns of association were obtained only for one of the 4 environments, the ShSt environment. This suggests that the relevance of human value orientation in shaping decision-making depends on the environmental properties even though participants performed the same computations (i.e. deciding when to leave collecting rewards in a current tree and travel somewhere else) across the 4 environments.

The present study was designed to assess the neurobiological mechanisms that track patch-switching threshold learning. This was done by identifying the extent to which single-trial brain activity correlated with the trial-wise fluctuation in the patch-switching threshold. Our analyses revealed such brain activity within the frontoparietal system. We would infer that frontoparietal activation possibly inhibits switching propensity as brain activity within frontal and parietal regions was associated with a lower patch-switching threshold. Specifically, the bilateral angular gyrus and right frontal gyrus activities were negatively associated with the patch-switching threshold. The angular gyrus has been implicated in a constellation of functions that can be related to the decision process involved in the tracking of the patch-switching threshold such as the manipulation of different numerical operations (Roland and Friberg 1985; Dehaene et al. 1998, 2003), memory retrieval (Ciaramelli et al. 2008; Vilberg and Rugg 2008), and conflict resolution (Wager et al. 2005; Nee et al. 2007). Notably, diffusion tensor imaging and tractography analysis showed that the angular gyrus is connected to the inferior, middle, and superior frontal gyrus, and the frontal gyrus was the other region that was found to track the patch-switching threshold in the present work. Specifically, the angular gyrus is connected to the inferior frontal gyrus via the third branch of the superior longitudinal fascicle (Frey et al. 2008), to the middle frontal gyrus via the second branch of the superior longitudinal fascicle (Makris et al. 2005), and the superior frontal gyrus via the occipitofrontal fascicle (Makris et al. 2007). Both the angular gyrus and the superior frontal gyrus tracked the patch-switching threshold and future studies could examine whether functional coupling between these regions is important for learning-induced changes in this decision variable. This working model can be assessed in future investigations that transiently experimentally disrupt this documented frontoparietal connectivity, for example, with the use of brain stimulation techniques such as transcranial direct current or magnetic stimulation as was done previously for other aspects of cognition such as working memory (Jones et al. 2017; Zacharopoulos et al. 2020). Moreover, such future investigations can examine whether the impact of this experimentally induced disruption is also dependent on self and other values, particularly in the ShSt environment. Another possibility for why brain activity within frontal and parietal regions was associated with a lower patch-switching threshold could also be related to differences in control demands between environments. Specifically, additional analyses (Supporting Information 14) revealed that participants take more harvest actions before exiting in environments with lower patch-leaving thresholds. Thus, participants might need increased attentional control to leave at the right time, as compared with an environment with a high leaving threshold, where fewer harvest actions are needed before exit.

This study was designed for discerning the extent to which the activity of these frontoparietal regions was uniquely associated with certain patch-switching computations. We found that the contribution of frontoparietal regions went well beyond the computation of the patch-switching threshold to include the relative forage value, reaction time/task difficulty, and harvest value. Similar to the prior work (Cohen Kadosh et al. 2005; Kolling et al. 2012), we might assume that the engagement of these regions across several patch-switching computations (e.g. harvest value, decision) depends on the entrenched property of the frontoparietal network in encoding magnitude, which was both essential and pervasive in guiding optimal behavior in the current behavioral ecology task. For example, a classic effect in the literature that applies in the context of the frontoparietal network is the distance effect, which states that the closer 2 compared magnitudes (e.g. 2 numbers such as the harvest value vs the patch-switching threshold in the present study), the more difficult the comparison, and the greater the activity of this frontoparietal network, and this finding was shown both for nonsocial and social processing (such as beauty comparisons) (Cohen Kadosh et al. 2005; Nieder and Dehaene 2009; Kedia et al. 2014). In our task, the computational parameter that is most related to the distance effect is the relative forage value as it encodes the numerical relationship between the individual's threshold (patch-switching threshold) and the reward value (harvest value), which we found was negatively related to reaction time (Supporting Information 5) indicating that the higher the distance the faster the reaction time.

Another important conclusion from the present study is that the behavioral and brain findings detailed above were similar in the personal and social conditions. An explanation of this may be that, across evolution, the need to protect loved ones equipped the foraging brain with biologically entrenched patch-switching mechanisms that operate for both direct and indirect rewards. In the present study, the “other” reward recipient was chosen by the participants and thus we could infer an underlying familiarity and associated effort to do well also in the “other” conditions. Indeed, the level of social focus on values within all of our participants was overall higher than the mean level of self-focus, consistent with cross-cultural research showing a relative dominance of social-focus values (Schwartz and Bardi 2001; Hanel et al. 2019). This dominance of social-focus values is consistent with the idea that there may be a default integration of others’ needs as a part of the basic motivational goal hierarchy within humans. Despite our finding that the behavioral and brain findings detailed above were similar in the personal and social conditions, we do acknowledge that our study’s sample size may have prevented the detection of potential smaller effect sizes that can be captured in future studies employing larger samples.

Nonetheless, the associations between self- and social-focus values and the task-switching thresholds in this research indicate that the relative personal priority attached to these values matters in the prediction of foraging behavior. It would be interesting to introduce measures of these values in paradigms with a wider range of social meanings attached to the foraging behavior. For example, future studies could test whether the behavioral and neurobiological isomorphism across the personal and social conditions is disrupted when participants are asked to forage on behalf of an unfamiliar, and unrelated “other,” or even a perceived adversary (e.g. a member of a different political party), and whether this disruption is dependent on personal levels of self- and social-focused values.

Of note, one potential limitation of the current study stems from the fact that choice uncertainty typically increases up to the patch-switching threshold. As a result, variables that correlate with a greater likelihood of exiting (or that code for the exit decision itself) are typically conflated with increasing levels of choice uncertainty. Therefore, some of the activations in response to exit decisions and relative forage value could reflect increases in choice uncertainty and associated monitoring/control functions encoded in regions such as the dACC (Shenhav et al. 2014). However, this is not the case in designs where there are many trials where participants substantially exceed their current exit threshold (Fontanesi et al. 2022). To this end, our paradigm was designed in a way that featured a great number of trials where participants substantially exceeded their current exit threshold. This is illustrated by the fact that the empirical patch-switching threshold in the present study was not static but changed robustly and predictably (Fig. 2) between orchards in response to our manipulation.

Another potential limitation of this study is the presence of a certain amount of collinearity between the parametric modulator pair relative forage value and harvest value. However, our choice for utilizing a single GLM that even included related predictors was to allow us to identify the unique variance in brain activity of certain predictors over and above that of other predictors. This may also explain the seemingly contradictory findings between parametric modulators that are negatively related to each other. Specifically, similar frontoparietal regions were positively associated with both the relative forage value and the harvest value, which seems to track the unique (but not shared) variance between these 2 negatively related regressors. Moreover, to reduce the possibility that the imaging results were conflated by choice uncertainty, we statistically controlled for reaction time, which is typically strongly associated with choice uncertainty/task difficulty. However, we acknowledge that reaction time measures can be noisy and that sequential choices can be complicated by the fact that participants may have partly prepared responses based on the previous trials. Another point of consideration is that there are different possible ways to conceptualize and, in turn, quantify the concept of the patch-switching threshold. For example, it can be quantified as the mean (objective) experienced reward rate across the environment or the mean (subjective) reward rate for the environment (e.g. estimated using a delta learning rule). However, here we aimed at using a more direct and intuitive measure that best captures learning-related changes directly derived from the participants’ behavior (for details, see Materials and Methods), which is also the standard way of measuring patch-switching threshold in the prior behavioral work (Constantino and Daw 2015; Zacharopoulos et al. 2018). Moreover, as can be seen behaviorally (Fig. 2), the patch-switching threshold was empirically modulated as expected in response to the experimental manipulation (Supporting Information 8). Furthermore, additional analyses (Supporting Information 15) showed that variability in the patch-switching threshold significantly reduced with time (*P* < 0.01), and these data provide additional evidence that our patch-switching measure is an adequate measure to capture learning-induced changes.

Future work could expand the current findings in a number of ways. First, future studies can employ more detailed behavioral modeling, inspired by well-established theoretical frameworks such as the marginal value theorem and the temporal-difference learning as was done previously (Constantino and Daw 2015), to elucidate more explicitly how variables are learned, and provide different computational fits for the personal and social patch-switching behavior. Second, and relatedly, future work building on the current findings could identify formal learning markers such as decreasing variance in the patch-switching threshold or reductions in the deviations from optimal switching behavior and assess how these are modulated depending on the reward recipient, the human value orientation, and the foraging orchard. Third, such information provided from the above 2 points can be used to generate novel regressors that are specific to the neurobiological mechanisms of patch-switching learning and updating, as opposed to merely tracking the patch-switching threshold as was done here. Taken together, such expansions of the current work have the potential to motivate greater specificity in behavioral and neural analyses allowing a more detailed understanding of the neurobiological mechanisms that shape foraging behavior.

In our complementary analyses, the harvest value predictor (Supporting Information 16) yielded frontoparietal positive brain activity even when the relative forage value was not featured in the same GLM model, and the same predictor (harvest value) yielded frontoparietal negative brain activity only when the relative forage value was not featured in the same GLM model. The patch-switching threshold was associated with frontoparietal negative brain activity even when the harvest value was not featured (but the relative forage value was featured) in the same GLM model, but the same predictor (patch-switching threshold) did not yield suprathreshold brain activity when the harvest value was featured (but the relative forage value was not featured) in the same GLM model. Indeed, one of the main aims of the present study was to bring together all the different parameters that influence foraging decisions and tease apart their unique contributions in modulating brain activity. Our additional GLM analyses (Supporting Information 16) show the relevance of close attention to specific designs and combinations of predictors in a design because they may influence the effects associated with the different predictors and consequently the interpretation of results.

In sum, the present research yielded preliminary evidence (i) that the patch-switching threshold is tracked by activity within frontoparietal regions including the angular gyrus and frontal areas whose activation possibly inhibits switching propensity, (ii) that overlapping frontoparietal regions (angular and frontal gyrus) track several patch-switching properties, (iii) for an isomorphism of behavioral and neural effects for personal and social patch-switching, and (iv) that human value orientation can be related to the patch-switching threshold in specific foraging environments. These findings expand on the decision-making literature, by illuminating a novel neurobiological understanding of how learning in switching tasks emerges neurocomputationally with implications for diverse real-life tasks.

## Supplementary Material

SM_bhad088

## Data Availability

The data underlying this article will be shared on reasonable request to the corresponding author.
