# Supplementary material for: Dissecting the neurocomputational bases of patch-switching
Source: Cereb Cortex. 2023 Mar 16;33(12):7930–40. doi: 10.1093/cercor/bhad088 (PMC10267616; doi:10.1093/cercor/bhad088)

Supporting Information

Supporting Information 1. A list of significant brain activations in response to each parametric modulator (first column: name of the region and the direction of the association is indicated by a positive or negative sign for positive and negative associations respectively, second column: the cluster-wise P_{FWE} derived from non-parametric methods, third column: cluster size, the next three columns show the x, y, and z coordinates of each region respectively, the last column shows the multiple comparisons FDR corrected P value across all significant clusters).

Region (+=Positive or -=Negative association)	P_{FWE}	k	x	y	z	P_{FDR}
**Decision**						
R superior frontal gyrus (+)	.008	1221	0	58	2	0.018667
L middle occipital/angular gyrus (+)	.032	700	-40	-60	20	0.043313
L middle temporal gyrus (+)	.026	811	-58	-12	-16	0.042
L precuneus (+)	.011	1138	-10	-52	32	0.0231
dACC/paracingulate gyrus (-)	.003	1823	4	24	42	0.0126
R insula (-)	.001	3765	32	18	4	0.0084
R angular gyrus (-)	.002	2130	46	-56	52	0.0126
L thalamus (-)	.004	1545	-12	-18	4	0.012923
L cerebellum (-)	.008	1325	-32	-58	-38	0.018667
L supramarginal gyrus (-)	.004	1536	-46	-34	38	0.012923
**Reaction time/Task difficulty**						
R superior frontal gyrus (+)	.001	55741	2	-4	54	0.0084
R middle frontal gyrus (+)	.035	559	28	38	26	0.0444
L middle frontal gyrus (+)	.024	673	-32	38	34	0.04032

L middle occipital gyrus (-)	.048	502	-50	-68	36	0.048
R angular gyrus (-)	.041	564	44	-62	50	0.0462
R corpus callosum (-)	.043	345	18	-24	26	0.0462
L superior frontal gyrus (-)	.04	386	-14	32	56	0.0462
**Patch-switching threshold**						
R angular gyrus (-)	.003	2399	36	-66	46	0.0126
R precentral gyrus (-)	.001	3813	52	10	16	0.0084
R superior frontal gyrus (-)	.021	546	4	28	44	0.038348
R superior occipital gyrus (-)	.029	361	12	-68	32	0.042
L angular gyrus (-)	.008	1251	-34	-60	40	0.018667
R thalamus (-)	.029	411	10	-14	6	0.042
L insula (-)	.043	288	-34	18	0	0.0462
**Harvest value**						
R angular gyrus (+)	.003	1581	36	-68	46	0.0126
L angular gyrus (+)	.004	896	-52	-48	48	0.012923
PCC/dACC (+)	.017	511	0	-28	26	0.032455
R inferior frontal gyrus (+)	.036	317	54	12	16	0.0444
R insula (+)	.044	245	34	18	4	0.0462
R frontal pole (+)	.033	323	42	50	8	0.043313
R thalamus (+)	.037	312	12	-14	6	0.0444
**New patch**						
L cerebellum (-)	.006	1418	-4	-42	-2	0.0168
L ventricle/dACC (-)	.011	891	-6	-4	24	0.0231
L hippocampus (-)	.048	359	-22	-40	6	0.048
**Relative forage value**						

R angular gyrus (+)	.001	2103	36	-68	46	0.0084
R inferior frontal gyrus (+)	.001	1678	56	14	14	0.0084
L angular gyrus (+)	.003	862	-52	-48	48	0.0126
R frontal pole (+)	.005	699	42	50	8	0.015
R superior frontal gyrus (+)	.012	489	4	28	40	0.024
PCC/dACC (+)	.029	253	2	-28	26	0.042
R middle frontal gyrus (+)	.03	250	24	10	50	0.042
**Travel time*source**						
L insula (+)	.023	382	-38	6	0	0.04025

Supporting Information 2. Imaging results in response to the parametric modulator of decision- positive association (**A**, **B**), decision- negative association (**C**, **D**), harvest value- positive association (**E**, **F**), and relative forage value- positive association (**G**, **H**).

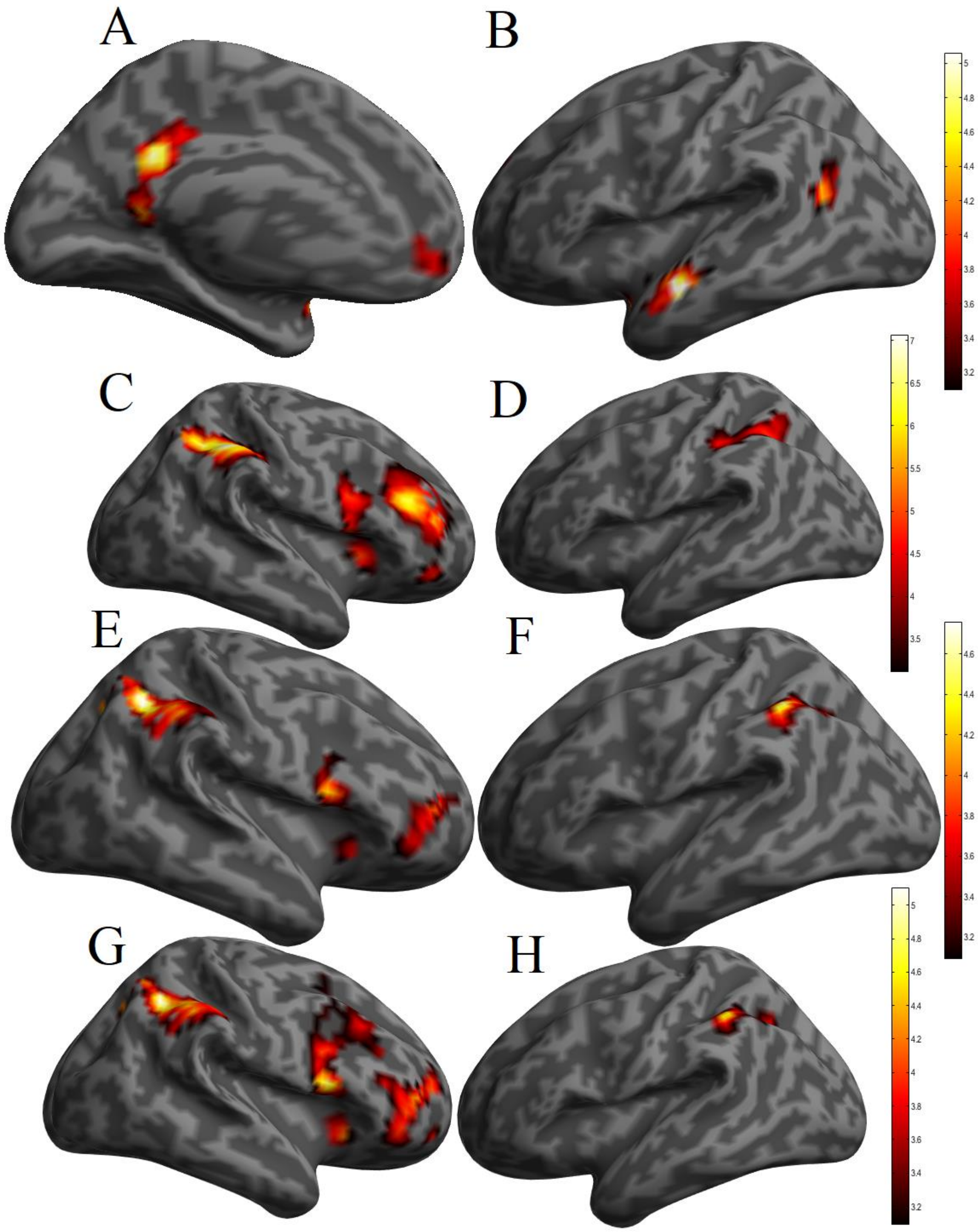

Supporting Information 3. Lateral and medial view imaging results in response to the parametric modulator of decision- positive association (**A**), decision- negative association

(**B**), harvest value- positive association (**C**), new patch- negative association (**D**), patch-switching threshold- negative association, (**E**) relative forage value- positive association (**F**).

A) Decision (Positive association)

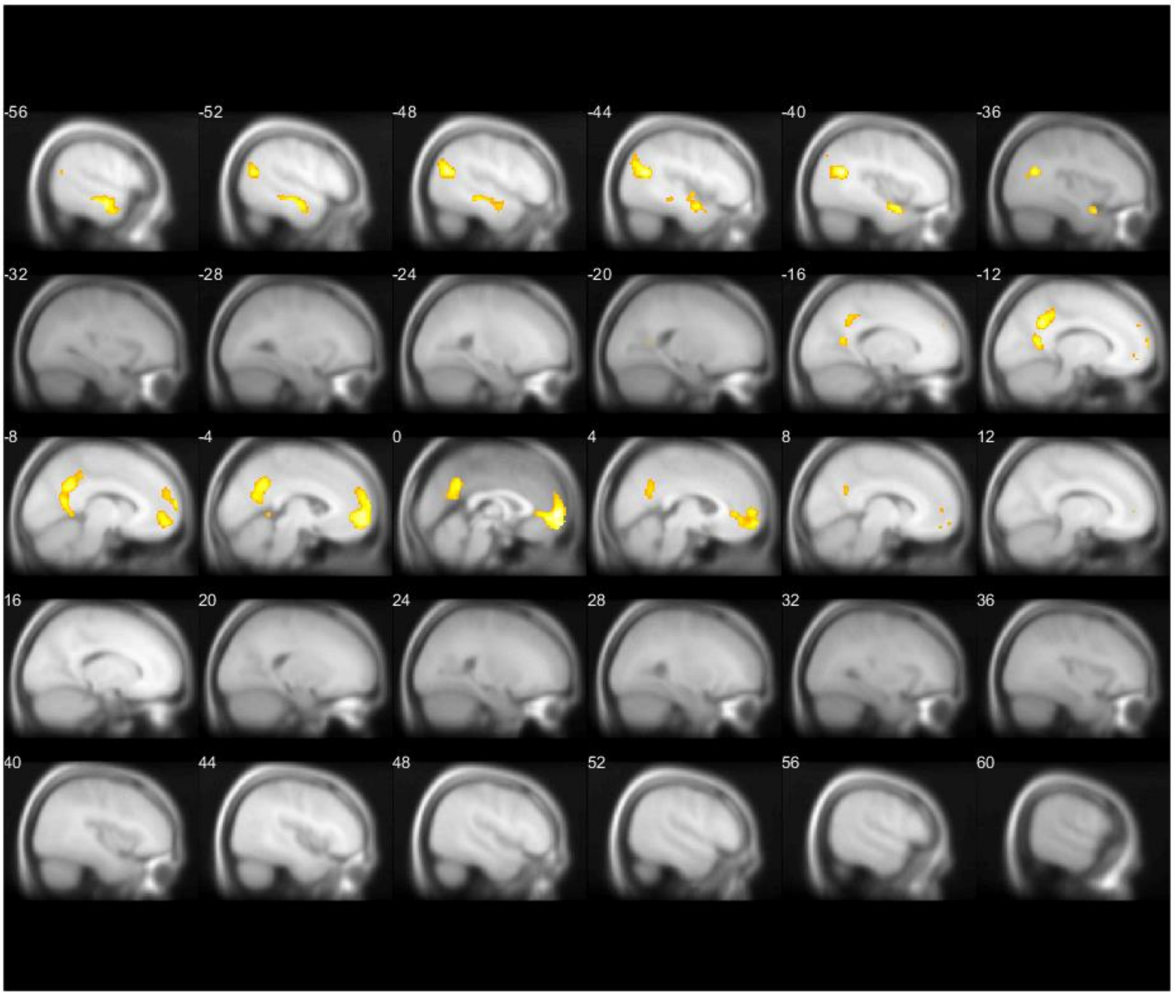

B) Decision (Negative association)

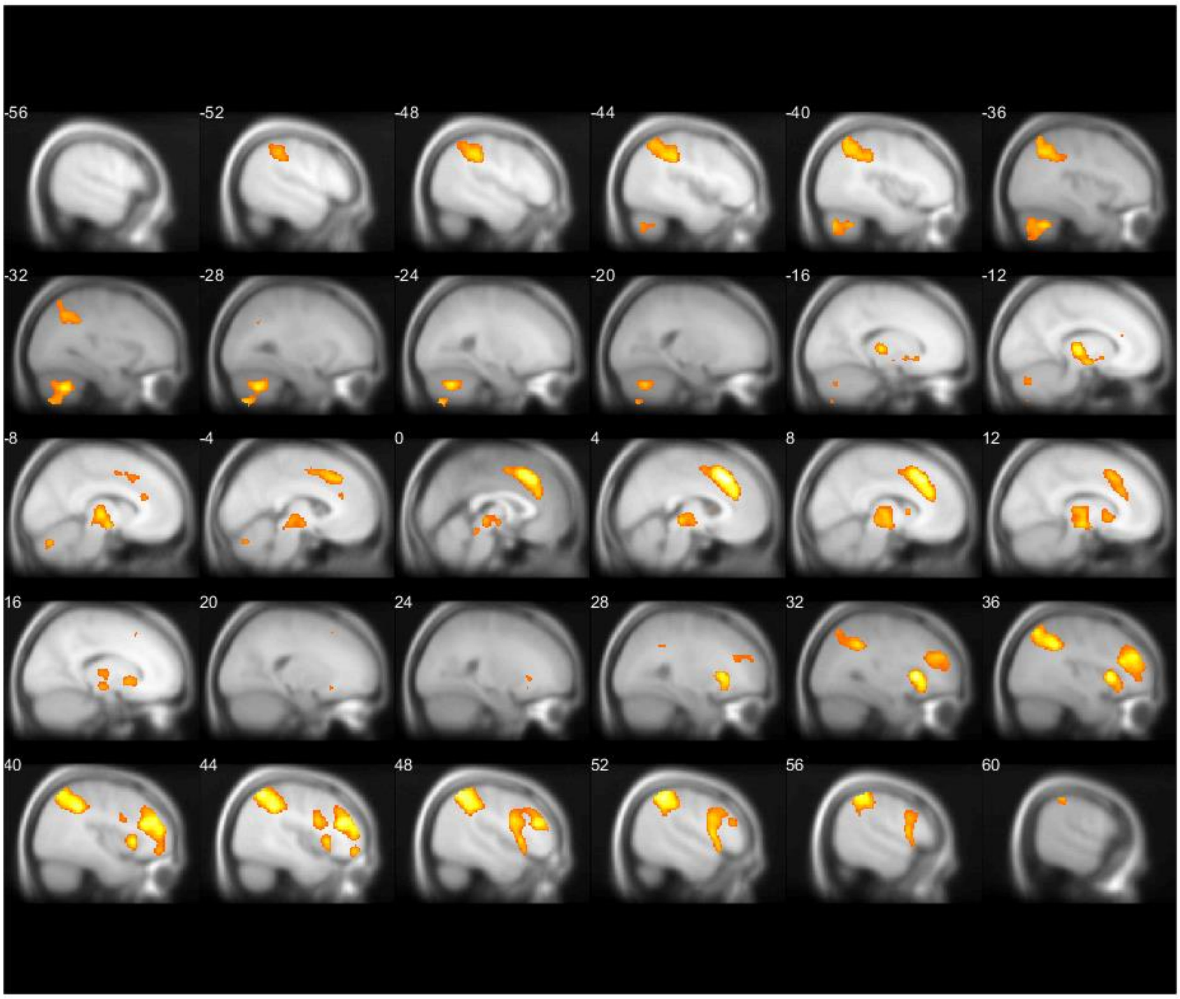
-56
-52
-48
-44
-40
-36
-32
-28
-24
-20
-16
-12
-8
-4
0
4
8
12
16
20
24
28
32
36
40
44
48
52
56
60

C) Harvest value (Positive association)

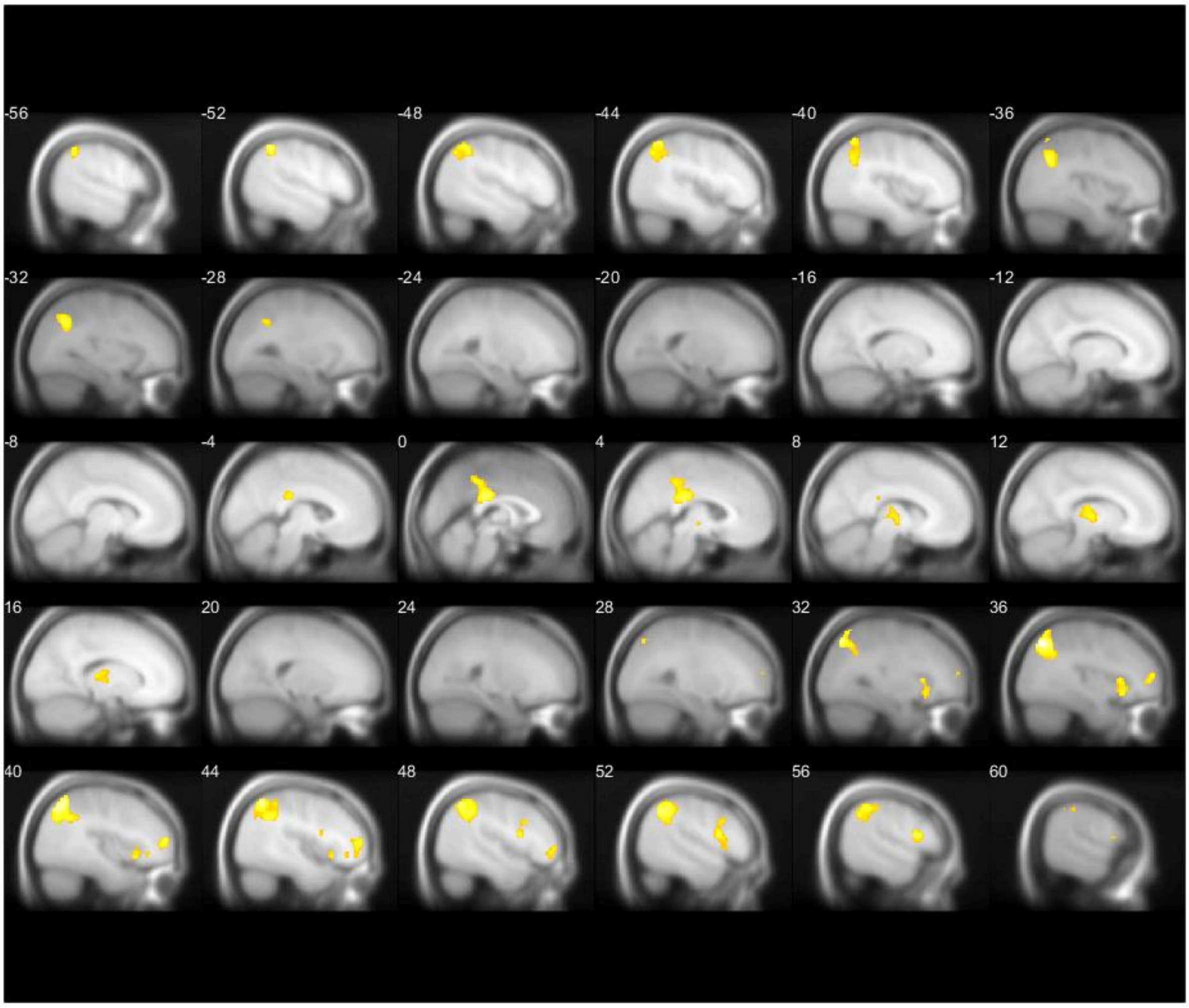

D) New patch (Negative association)

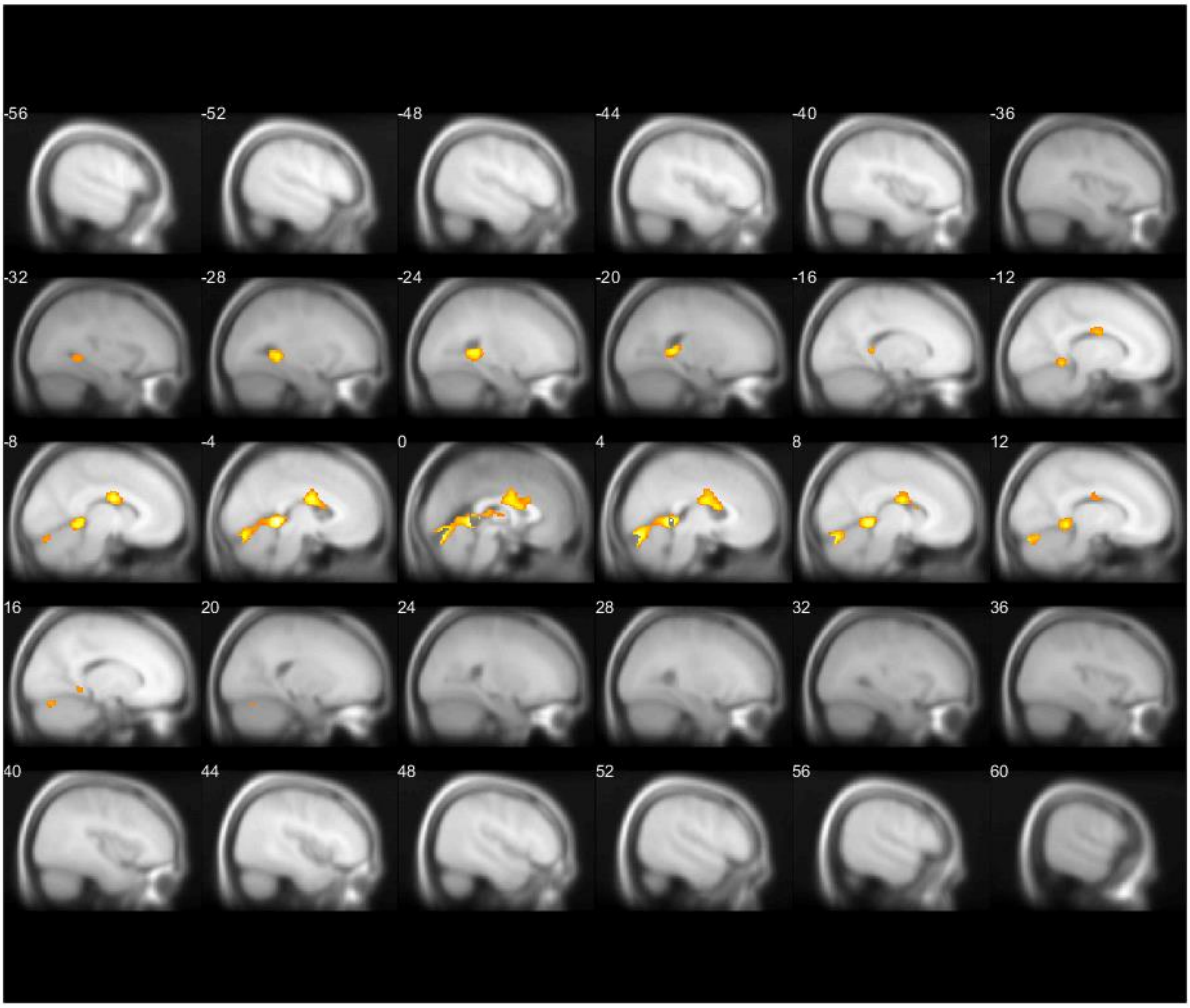

E) Patch-switching threshold (Negative association)

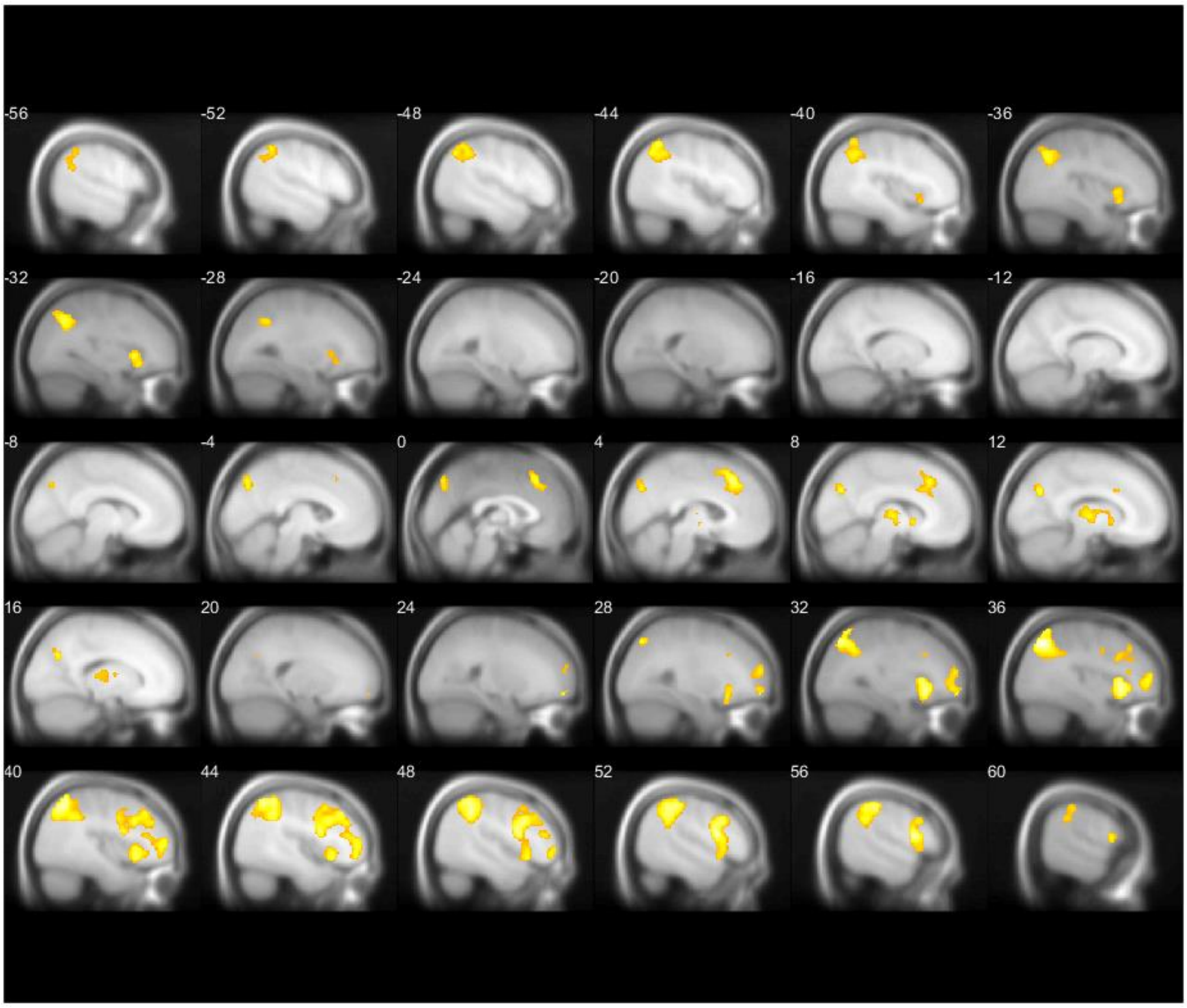

F) Relative forage value (Positive association)

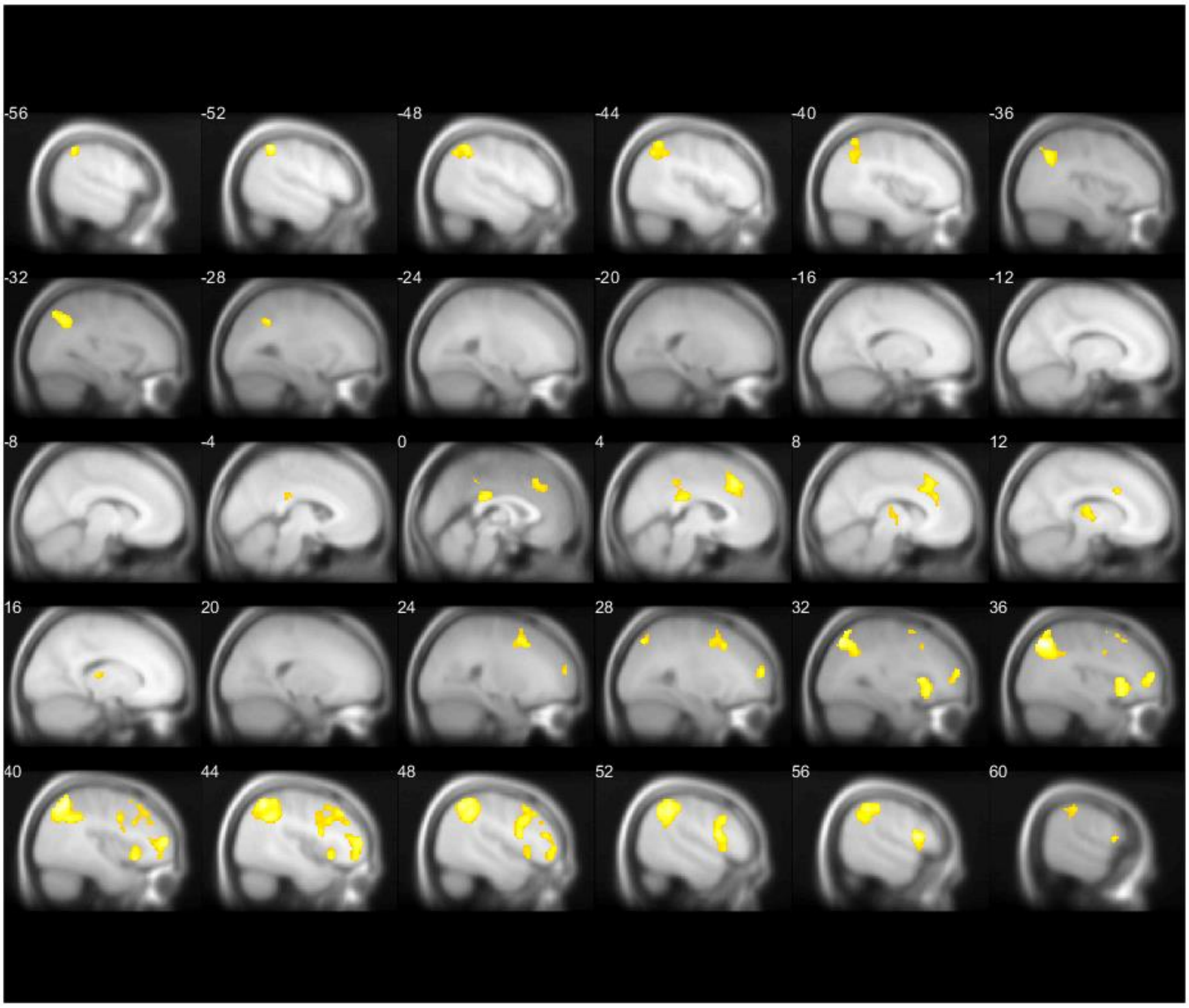
-56
-52
-48
-44
-40
-36
-32
-28
-24
-20
-16
-12
-8
-4
0
4
8
12
16
20
24
28
32
36
40
44
48
52
56
60

Supporting Information 4. The predictors of the GLM. This example is from a subsection of a single run from one participant.

Supporting Information 4A. Raw values are displayed on the left-hand side, and the corresponding convolved HRF signal is displayed on the right-hand side. The x-axis units on the left-hand side are expressed in the number of trials while the x-axis units on the right-hand side are expressed in scans.

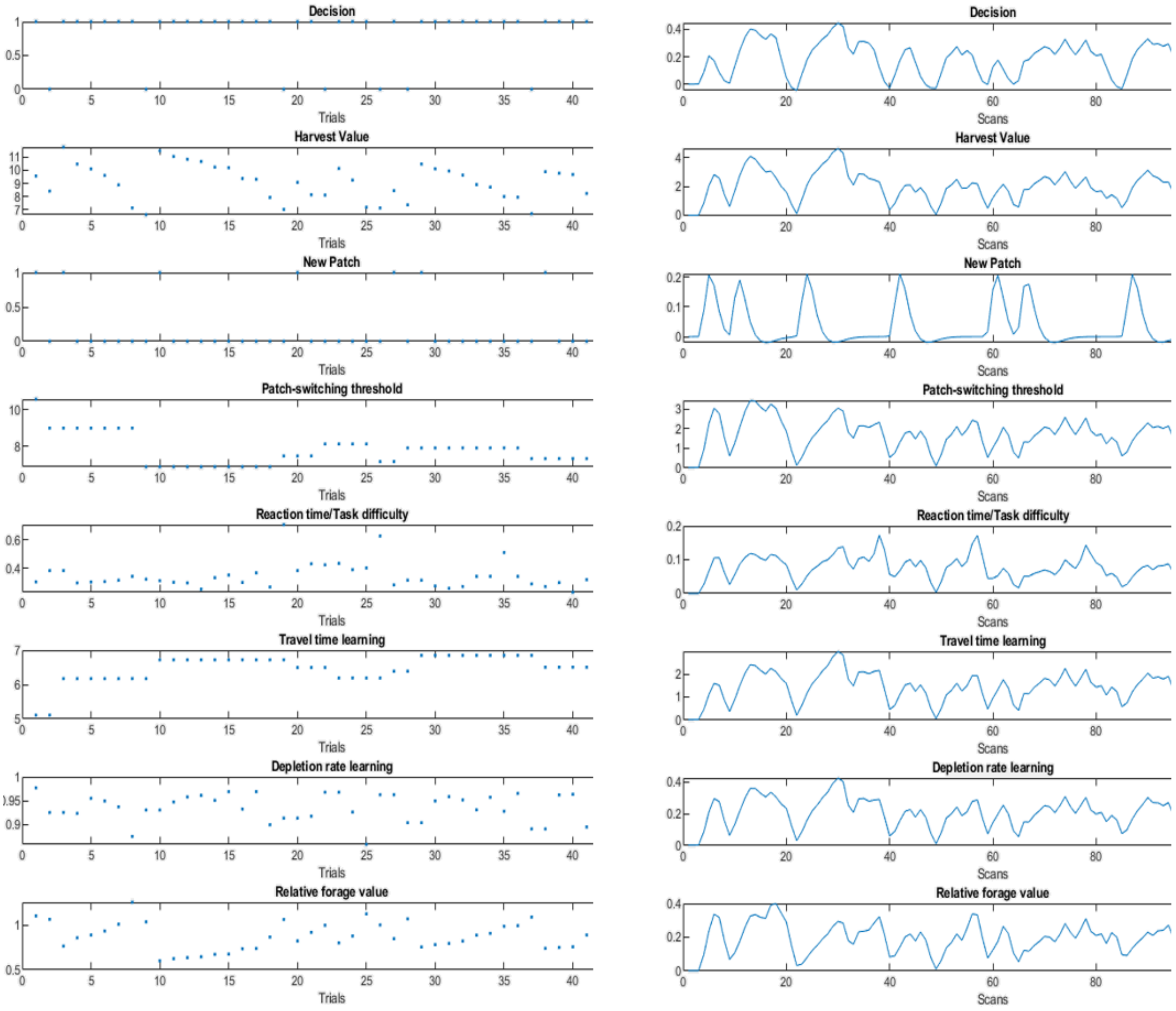

Supporting Information 4B. Demeaned values are displayed on the left-hand side, and the corresponding convolved HRF signal is displayed on the right-hand side. The x-axis units on

the left-hand side are expressed in the number of trials while the x-axis units on the right-hand side are expressed in scans.

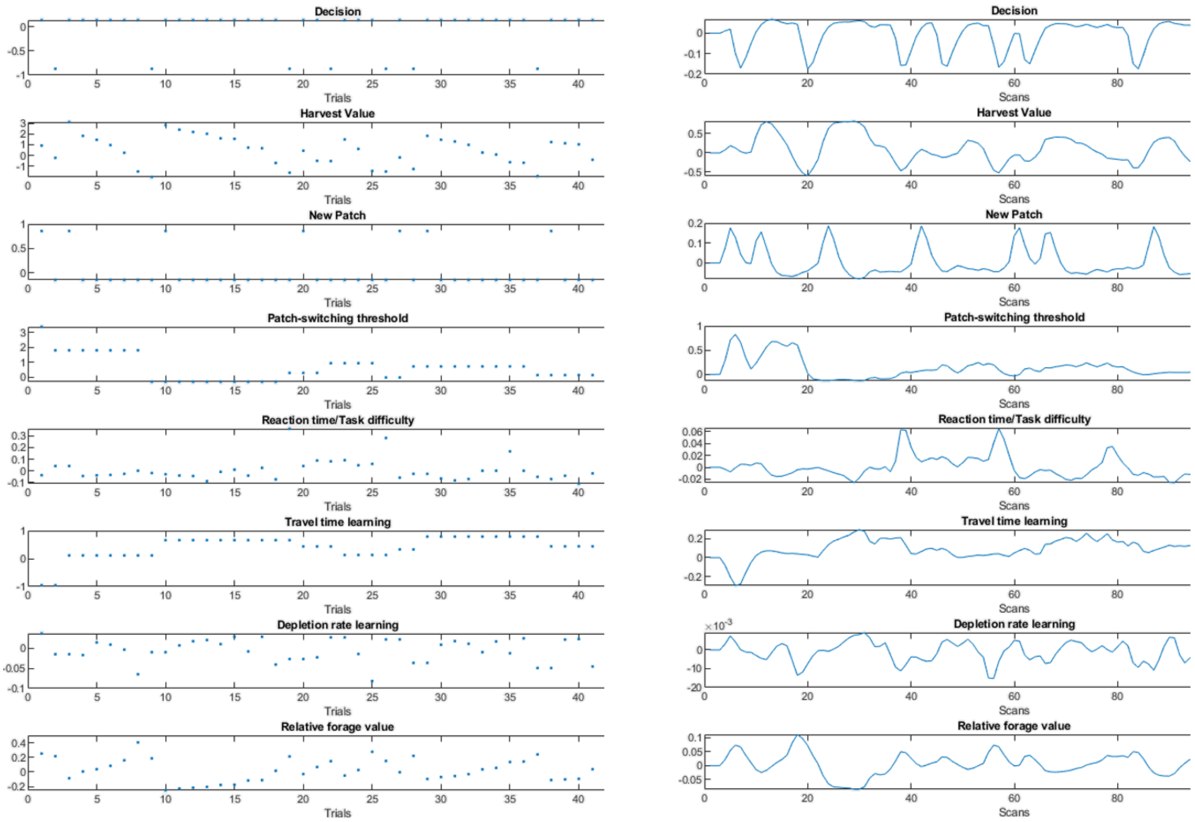

Supporting Information 5.

By utilizing a mixed-effects model, with participants as a random effect, we showed that the distance between the patch-switching threshold and harvest value computed as the absolute value of the (relative forage value-1) was negatively related to reaction time (B=-0.07, SE= 0.01, DF=11973, T=-8.59, P<.0001) indicating that the higher the distance the faster the reaction time.

Supporting Information 6.

Additional information depicting how several parameters changed per participant, run, and block. (P=Participant, Run=run number, Block=block number, Personal=whether the block was personal (=1) or social (=0), Long=whether the block was long (=1) or short (=0), Steep=whether the block was shallow (=1) or steep (=0)).

P	Run	Block	Personal	Long	Shallow
1	1	1	1	1	1
1	1	2	0	1	1
1	1	3	0	1	1
1	1	4	1	1	1
1	2	5	0	1	0
1	2	6	1	1	0
1	2	7	1	1	0
1	2	8	0	1	0
1	3	9	0	1	0
1	3	10	1	1	0
1	3	11	1	1	0
1	3	12	0	1	0
1	4	13	1	1	1
1	4	14	0	1	1
1	4	15	0	1	1
1	4	16	1	1	1
2	1	1	1	1	1
2	1	2	0	1	1
2	1	3	0	1	1
2	1	4	1	1	1
2	2	5	0	1	0
2	2	6	1	1	0
2	2	7	1	1	0
2	2	8	0	1	0
2	3	9	0	1	0
2	3	10	1	1	0
2	3	11	1	1	0
2	3	12	0	1	0
2	4	13	1	1	1
2	4	14	0	1	1
2	4	15	0	1	1
2	4	16	1	1	1
3	1	1	1	1	1
3	1	2	0	1	1
3	1	3	0	1	1
3	1	4	1	1	1
3	2	5	0	1	0
3	2	6	1	1	0
3	2	7	1	1	0
3	2	8	0	1	0
3	3	9	0	1	0
3	3	10	1	1	0
3	3	11	1	1	0
3	3	12	0	1	0
3	4	13	1	1	1

3	4	14	0	1	1
3	4	15	0	1	1
3	4	16	1	1	1
4	1	1	1	1	1
4	1	2	0	1	1
4	1	3	0	1	1
4	1	4	1	1	1
4	2	5	0	1	0
4	2	6	1	1	0
4	2	7	1	1	0
4	2	8	0	1	0
4	3	9	0	1	0
4	3	10	1	1	0
4	3	11	1	1	0
4	3	12	0	1	0
4	4	13	1	1	1
4	4	14	0	1	1
4	4	15	0	1	1
4	4	16	1	1	1
5	1	1	1	0	1
5	1	2	0	0	1
5	1	3	0	0	1
5	1	4	1	0	1
5	2	5	0	0	0
5	2	6	1	0	0
5	2	7	1	0	0
5	2	8	0	0	0
5	3	9	0	1	0
5	3	10	1	1	0
5	3	11	1	1	0
5	3	12	0	1	0
5	4	13	1	1	1
5	4	14	0	1	1
5	4	15	0	1	1
5	4	16	1	1	1
6	1	1	1	0	1
6	1	2	0	0	1
6	1	3	0	0	1
6	1	4	1	0	1
6	2	5	0	0	0
6	2	6	1	0	0
6	2	7	1	0	0
6	2	8	0	0	0
6	3	9	0	1	0
6	3	10	1	1	0
6	3	11	1	1	0

6	3	12	0	1	0
6	4	13	1	1	1
6	4	14	0	1	0
6	4	15	0	1	1
6	4	16	1	1	1
7	1	1	1	0	1
7	1	2	0	0	1
7	1	3	0	0	1
7	1	4	1	0	1
7	2	5	0	0	0
7	2	6	1	0	0
7	2	7	1	0	0
7	2	8	0	0	0
7	3	9	0	1	0
7	3	10	1	1	0
7	3	11	1	1	0
7	3	12	0	1	0
7	4	13	1	1	1
7	4	14	0	1	1
7	4	15	0	1	1
7	4	16	1	1	1
8	1	1	1	0	1
8	1	2	0	0	1
8	1	3	0	0	1
8	1	4	1	0	1
8	2	5	0	0	0
8	2	6	1	0	0
8	2	7	1	0	0
8	2	8	0	0	0
8	3	9	0	1	0
8	3	10	1	1	0
8	3	11	1	1	0
8	3	12	0	1	0
8	4	13	1	1	1
8	4	14	0	1	1
8	4	15	0	1	1
8	4	16	1	1	1
9	1	1	1	0	1
9	1	2	0	0	1
9	1	3	0	0	1
9	1	4	1	0	1
9	2	5	0	0	0
9	2	6	1	0	0
9	2	7	1	0	0
9	2	8	0	0	0
9	3	9	0	1	0

9	3	10	1	1	0
9	3	11	1	1	0
9	3	12	0	1	0
9	4	13	1	1	1
9	4	14	0	1	1
9	4	15	0	1	1
9	4	16	1	1	1
10	1	1	1	0	1
10	1	2	0	0	1
10	1	3	0	0	1
10	1	4	1	0	1
10	2	5	0	0	0
10	2	6	1	0	0
10	2	7	1	0	0
10	2	8	0	0	0
10	3	9	0	1	0
10	3	10	1	1	0
10	3	11	1	1	0
10	3	12	0	1	0
10	4	13	1	1	1
10	4	14	0	1	1
10	4	15	0	1	1
10	4	16	1	1	1
11	1	1	1	0	1
11	1	2	0	0	1
11	1	3	0	0	1
11	1	4	1	0	1
11	2	5	0	0	0
11	2	6	1	0	0
11	2	7	1	0	0
11	2	8	0	0	0
11	3	9	0	1	0
11	3	10	1	1	0
11	3	11	1	1	0
11	3	12	0	1	0
11	4	13	1	1	1
11	4	14	0	1	1
11	4	15	0	1	1
11	4	16	1	1	1
12	1	1	1	0	1
12	1	2	0	0	1
12	1	3	0	0	1
12	1	4	1	0	1
12	2	5	0	0	0
12	2	6	1	0	0
12	2	7	1	0	0

12	2	8	0	0	0
12	3	9	0	1	0
12	3	10	1	1	0
12	3	11	1	1	0
12	3	12	0	1	0
12	4	13	1	1	1
12	4	14	0	1	1
12	4	15	0	1	1
12	4	16	1	1	1
13	1	1	1	0	1
13	1	2	0	0	1
13	1	3	0	0	1
13	1	4	1	0	1
13	2	5	0	0	0
13	2	6	1	0	0
13	2	7	1	0	0
13	2	8	0	0	0
13	3	9	0	1	0
13	3	10	1	1	0
13	3	11	1	1	0
13	3	12	0	1	0
13	4	13	1	1	1
13	4	14	0	1	1
13	4	15	0	1	1
13	4	16	1	1	1
14	1	1	1	0	1
14	1	2	0	0	1
14	1	3	0	0	1
14	1	4	1	0	1
14	2	5	0	0	0
14	2	6	1	0	0
14	2	7	1	0	0
14	2	8	0	0	0
14	3	9	0	1	0
14	3	10	1	1	0
14	3	11	1	1	0
14	3	12	0	1	1
14	4	13	1	1	1
14	4	14	0	1	1
14	4	15	0	1	1
14	4	16	1	1	1
15	1	1	1	0	1
15	1	2	0	0	1
15	1	3	0	0	1
15	1	4	1	0	1
15	2	5	0	0	0

15	2	6	1	0	0
15	2	7	1	0	0
15	2	8	0	0	0
15	3	9	0	1	0
15	3	10	1	1	0
15	3	11	1	1	0
15	3	12	0	1	0
15	4	13	1	1	1
15	4	14	0	1	1
15	4	15	0	1	1
15	4	16	1	1	1
16	1	1	1	0	1
16	1	2	0	0	1
16	1	3	0	0	1
16	1	4	1	0	1
16	2	5	0	0	0
16	2	6	1	0	0
16	2	7	1	0	0
16	2	8	0	0	0
16	3	9	0	1	0
16	3	10	1	1	0
16	3	11	1	1	0
16	3	12	0	1	0
16	4	13	1	1	1
16	4	14	0	1	1
16	4	15	0	1	1
16	4	16	1	1	1
17	1	1	1	0	1
17	1	2	0	0	1
17	1	3	0	0	1
17	1	4	1	0	1
17	2	5	0	0	0
17	2	6	1	0	0
17	2	7	1	0	0
17	2	8	0	0	0
17	3	9	0	1	0
17	3	10	1	1	0
17	3	11	1	1	0
17	3	12	0	1	0
17	4	13	1	1	1
17	4	14	0	1	1
17	4	15	0	1	1
17	4	16	1	1	1
18	1	1	1	0	1
18	1	2	0	0	1
18	1	3	0	0	1

18	1	4	1	0	1
18	2	5	0	0	0
18	2	6	1	0	0
18	2	7	1	0	0
18	2	8	0	0	0
18	3	9	0	1	0
18	3	10	1	1	0
18	3	11	1	1	0
18	3	12	0	1	0
18	4	13	1	1	1
18	4	14	0	1	1
18	4	15	0	1	1
18	4	16	1	1	1
19	1	1	1	0	1
19	1	2	0	0	1
19	1	3	0	0	1
19	1	4	1	0	1
19	2	5	0	0	0
19	2	6	1	0	0
19	2	7	1	0	0
19	2	8	0	0	0
19	3	9	0	1	0
19	3	10	1	1	0
19	3	11	1	1	0
19	3	12	0	1	0
19	4	13	1	1	1
19	4	14	0	1	1
19	4	15	0	1	1
19	4	16	1	1	1
20	1	1	1	0	1
20	1	2	0	0	1
20	1	3	0	0	1
20	1	4	1	0	1
20	2	5	0	0	0
20	2	6	1	0	0
20	2	7	1	0	0
20	2	8	0	0	0
20	3	9	0	1	0
20	3	10	1	1	0
20	3	11	1	1	0
20	3	12	0	1	0
20	4	13	1	1	1
20	4	14	0	1	1
20	4	15	0	1	1
20	4	16	1	1	1
21	1	1	1	0	1

21	1	2	0	0	1
21	1	3	0	0	1
21	1	4	1	0	1
21	2	5	0	0	0
21	2	6	1	0	0
21	2	7	1	0	0
21	2	8	0	0	0
21	3	9	0	1	0
21	3	10	1	1	0
21	3	11	1	1	0
21	3	12	0	1	0
21	4	13	1	1	1
21	4	14	0	1	1
21	4	15	0	1	1
21	4	16	1	1	1

Supporting Information 7.

Additional information from the 2*2*2 ANOVA which was reported in the main text, including estimates where the dependent variable is the patch-switching threshold (**Supporting Information 7.1.**) or the number of points (**Supporting Information 7.3.**). CI_L=95% confidence interval lower bound, CI_U=95% confidence interval upper bound, and posthoc comparisons where the dependent variable is the patch-switching threshold (**Supporting Information 7.2.**) or the number of points (**Supporting Information 7.4.**).

Supporting Information 7.1.

TravelTime	DepletionRate	Source	Mean	Std. Error	CI_L	CI_U
Short	Shallow	Personal	7.094	0.185	6.701	7.486
		Social	7.015	0.176	6.641	7.389
	Steep	Personal	5.613	0.215	5.157	6.069
		Social	5.683	0.219	5.218	6.148
Long	Shallow	Personal	5.777	0.242	5.265	6.29
		Social	5.895	0.304	5.251	6.539
	Steep	Personal	4.491	0.247	3.966	5.015
		Social	4.434	0.245	3.915	4.953

Supporting Information 7.2.

DepletionRate	Source	(I) TravelTime	(J) TravelTime	Mean Difference (I-J)	Std. Error	Sig	CI_L	CI_U
Shallow	Personal	Short	Long	1.316*	0.278	<.001	0.727	1.905
	Social	Short	Long	1.120*	0.308	0.002	0.466	1.773
Steep	Personal	Short	Long	1.122*	0.223	<.001	0.65	1.594
	Social	Short	Long	1.249*	0.219	<.001	0.785	1.712
**TravelTime**	**Source**	**(I) DepletionRate**	**(J) DepletionRate**	**Mean Difference (I-J)**	**Std. Error**	**Sig**	**CI_L**	**CI_U**
Short	Personal	Shallow	Steep	1.481*	0.268	<.001	0.913	2.049
	Social	Shallow	Steep	1.332*	0.277	<.001	0.745	1.919
Long	Personal	Shallow	Steep	1.287*	0.279	<.001	0.696	1.878
	Social	Shallow	Steep	1.461*	0.292	<.001	0.843	2.079
**TravelTime**	**DepletionRate**	**(I) Source**	**(J) Source**	**Mean Difference (I-J)**	**Std. Error**	**Sig**	**CI_L**	**CI_U**
Short	Shallow	Personal	Social	0.079	0.116	0.509	-0.168	0.325
	Steep	Personal	Social	-0.07	0.098	0.482	-0.277	0.137
Long	Shallow	Personal	Social	-0.118	0.162	0.479	-0.462	0.226
	Steep	Personal	Social	0.056	0.122	0.649	-0.201	0.314

Supporting Information 7.3.

TravelTime	DepletionRate	Source	Mean	Std. Error	CI_L	CI_U
Short	Shallow	Personal	752.048	16.730	716.581	787.514
		Social	751.877	13.141	724.018	779.735
	Steep	Personal	648.460	9.301	628.742	668.178
		Social	662.266	9.149	642.872	681.660
Long	Shallow	Personal	693.351	17.099	657.103	729.600
		Social	685.306	11.854	660.176	710.436
	Steep	Personal	588.316	7.563	572.284	604.348
		Social	581.906	10.609	559.417	604.395

Supporting Information 7.4.

DepletionRate	Source	(I) TravelTime	(J) TravelTime	Mean Difference (I-J)	Std. Error	Sig	CI_L	CI_U
Shallow	Personal	Short	Long	58.696	17.926	.005	20.695	96.698
	Social	Short	Long	66.571	14.169	<.001	36.534	96.607
Steep	Personal	Short	Long	60.144	9.557	<.001	39.885	80.403
	Social	Short	Long	80.360	12.087	<.001	54.737	105.983
**TravelTime**	**Source**	**(I) DepletionRate**	**(J) DepletionRate**	**Mean Difference (I-J)**	**Std. Error**	**Sig**	**CI_L**	**CI_U**
Short	Personal	Shallow	Steep	103.588	15.608	<.001	70.500	136.675
	Social	Shallow	Steep	89.611	11.245	<.001	65.771	113.450
Long	Personal	Shallow	Steep	105.035	16.365	<.001	70.344	139.727
	Social	Shallow	Steep	103.400	15.122	<.001	71.343	135.457
**TravelTime**	**DepletionRate**	**(I) Source**	**(J) Source**	**Mean Difference (I-J)**	**Std. Error**	**Sig**	**CI_L**	**CI_U**
Short	Shallow	Personal	Social	.171	13.791	.990	-29.064	29.406

	Steep	Personal	Social	-13.806	10.589	.211	-36.254	8.642
Long	Shallow	Personal	Social	8.045	15.772	.617	-25.389	41.480
	Steep	Personal	Social	6.410	8.117	.441	-10.796	23.616

Supporting Information 8.

Time course (in blue) depicting how the patch-switching threshold changes over time per participant, per environment (the corresponding mean patch-switching threshold is indicated by the dashed horizontal red line).

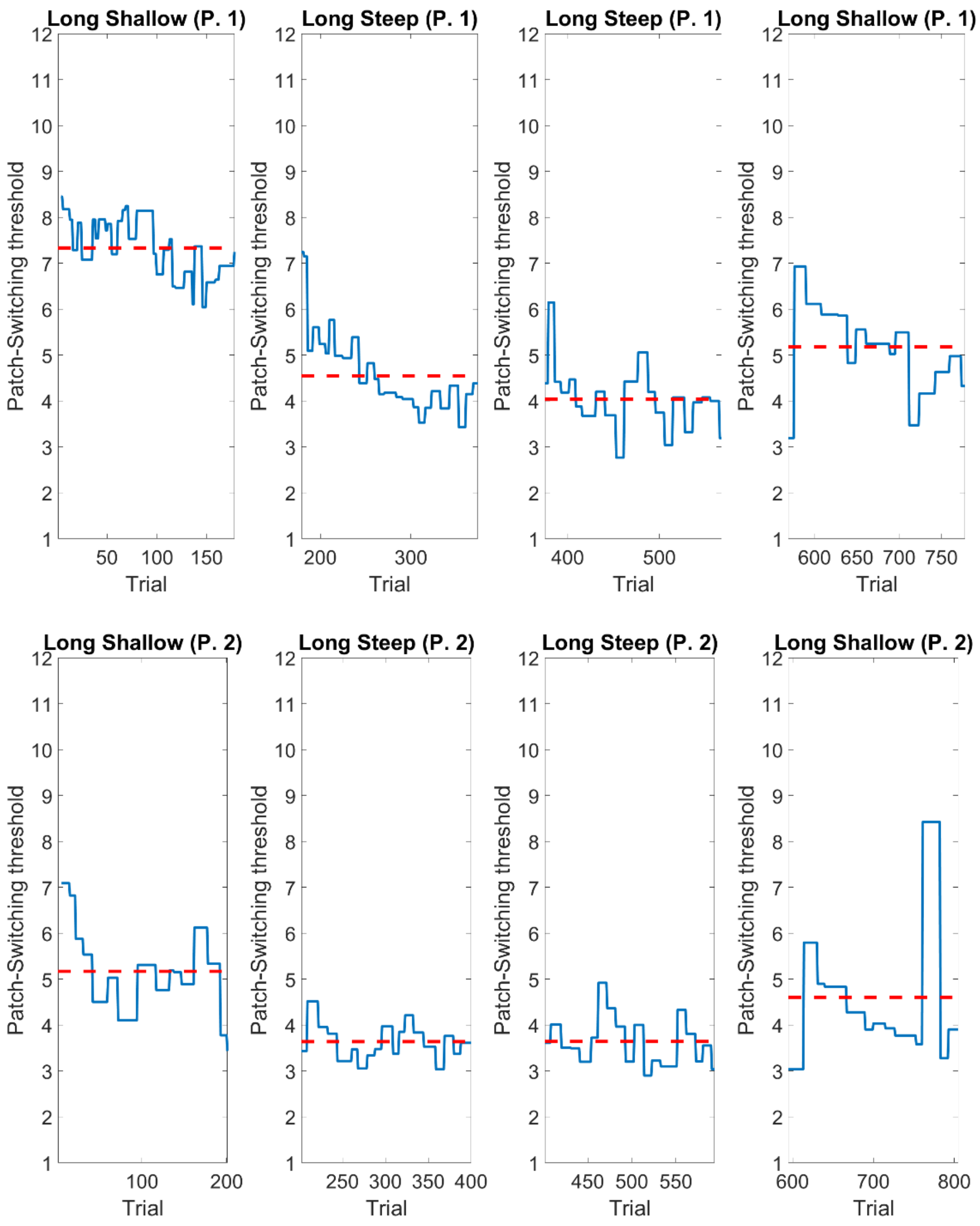

Long Shallow (P. 1)
Long Steep (P. 1)
Long Steep (P. 1)
Long Shallow (P. 1)
Long Shallow (P. 2)
Long Steep (P. 2)
Long Steep (P. 2)
Long Shallow (P. 2)
Patch-Switching threshold
Trial

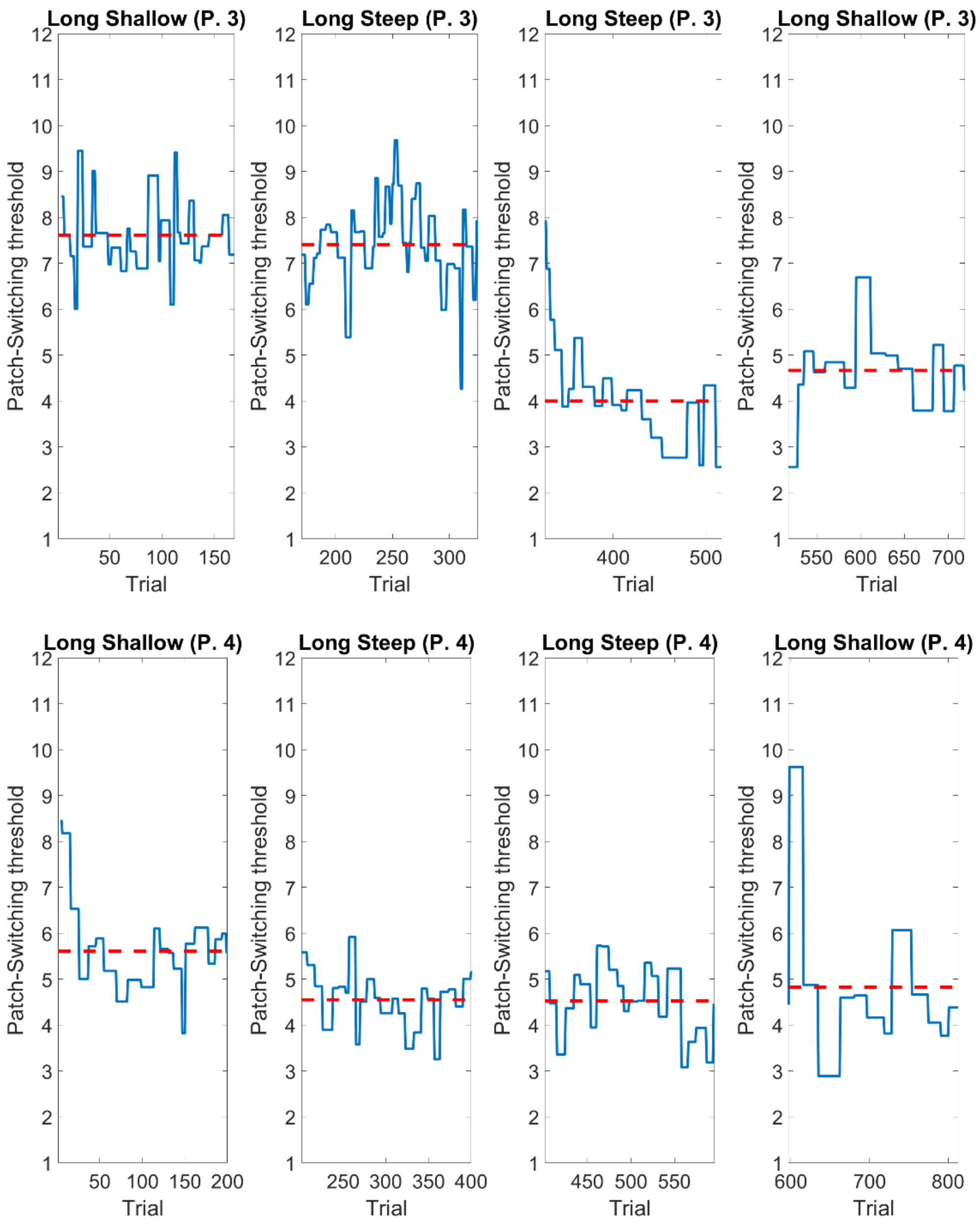
Long Shallow (P. 3)
Long Steep (P. 3)
Long Steep (P. 3)
Long Shallow (P. 3)
Long Shallow (P. 4)
Long Steep (P. 4)
Long Steep (P. 4)
Long Shallow (P. 4)
Patch-Switching threshold
Trial

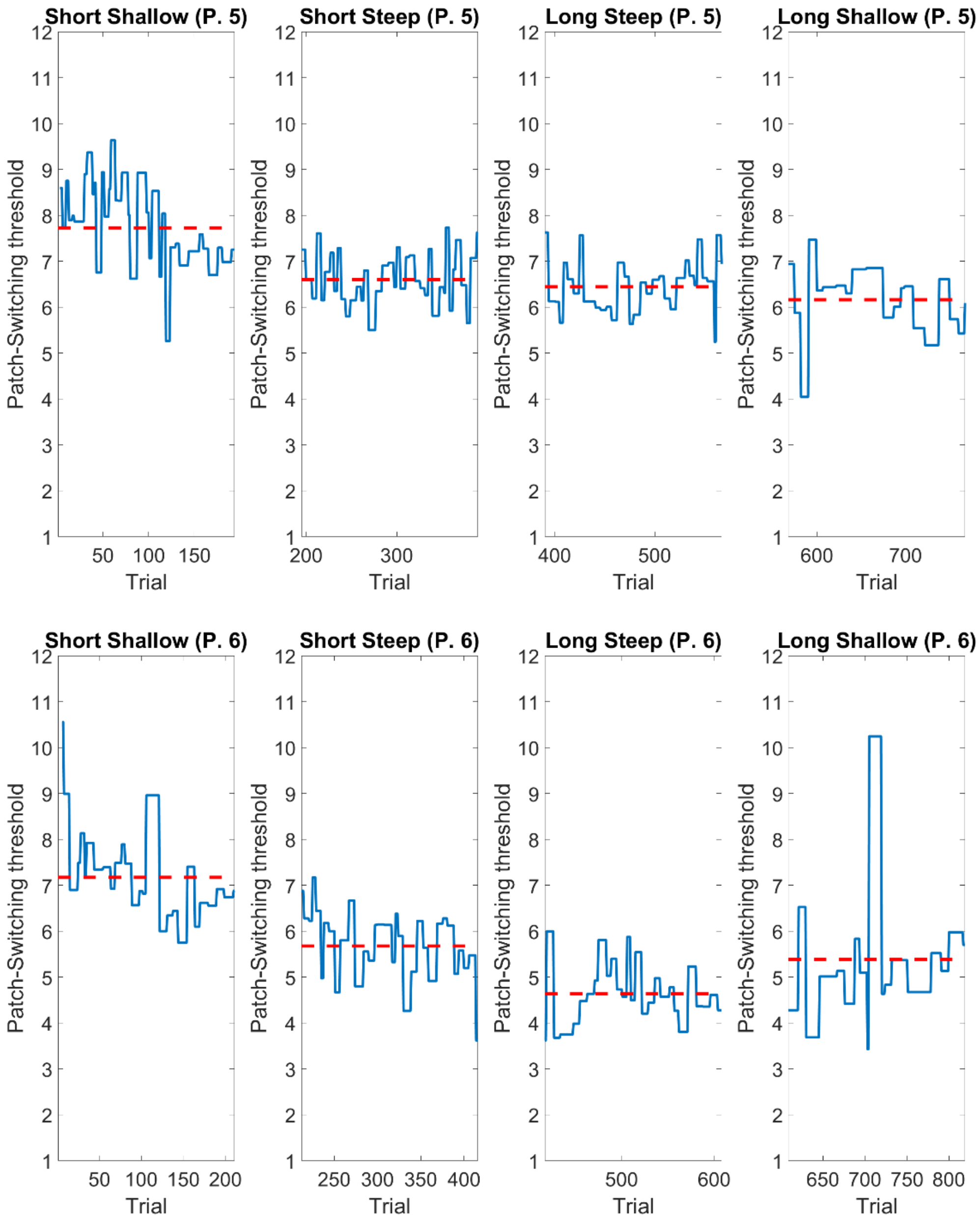
Short Shallow (P. 5)
Short Steep (P. 5)
Long Steep (P. 5)
Long Shallow (P. 5)
Short Shallow (P. 6)
Short Steep (P. 6)
Long Steep (P. 6)
Long Shallow (P. 6)
Patch-Switching threshold
Trial

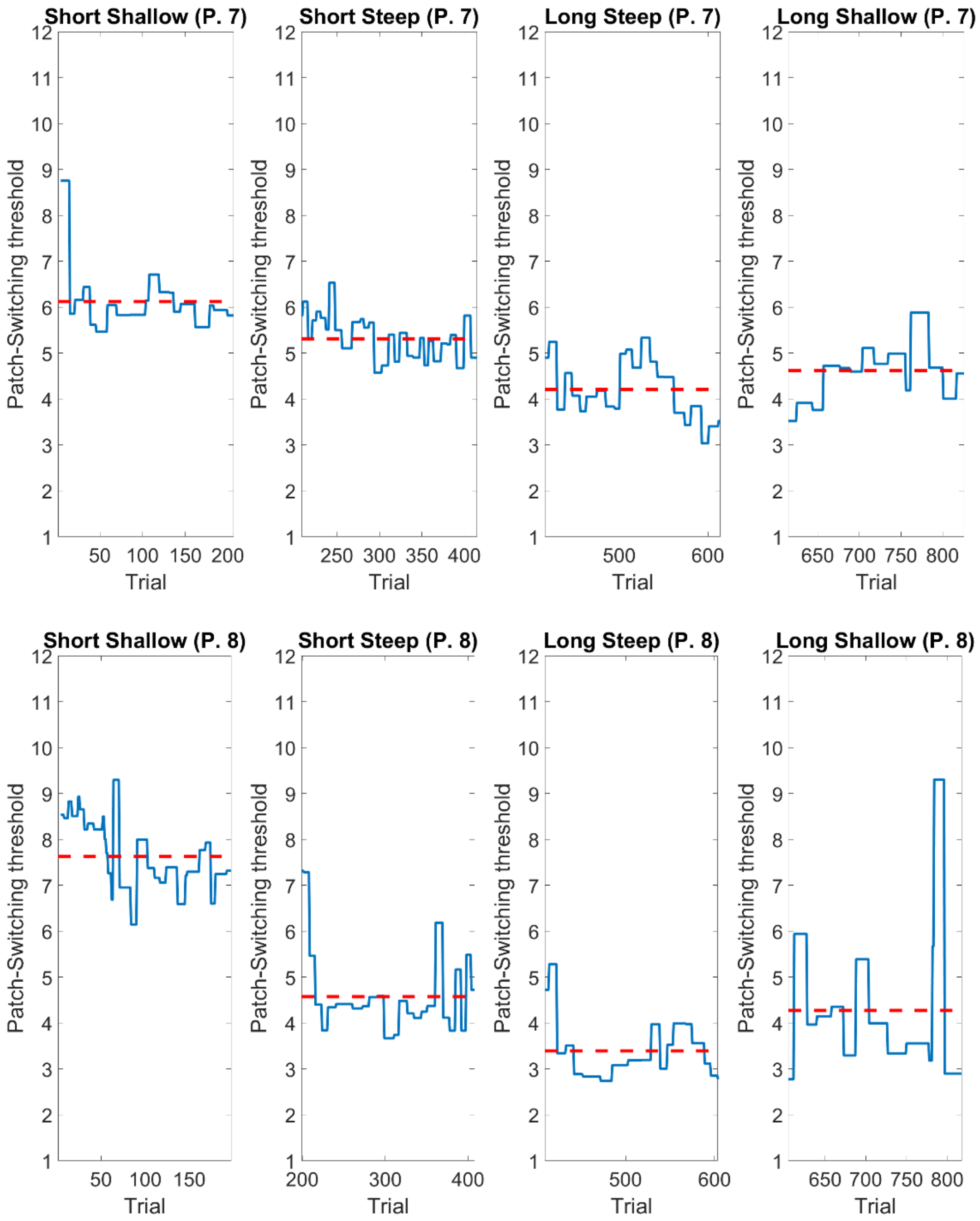

Short Shallow (P. 7)
Short Steep (P. 7)
Long Steep (P. 7)
Long Shallow (P. 7)
Short Shallow (P. 8)
Short Steep (P. 8)
Long Steep (P. 8)
Long Shallow (P. 8)
Patch-Switching threshold
Trial

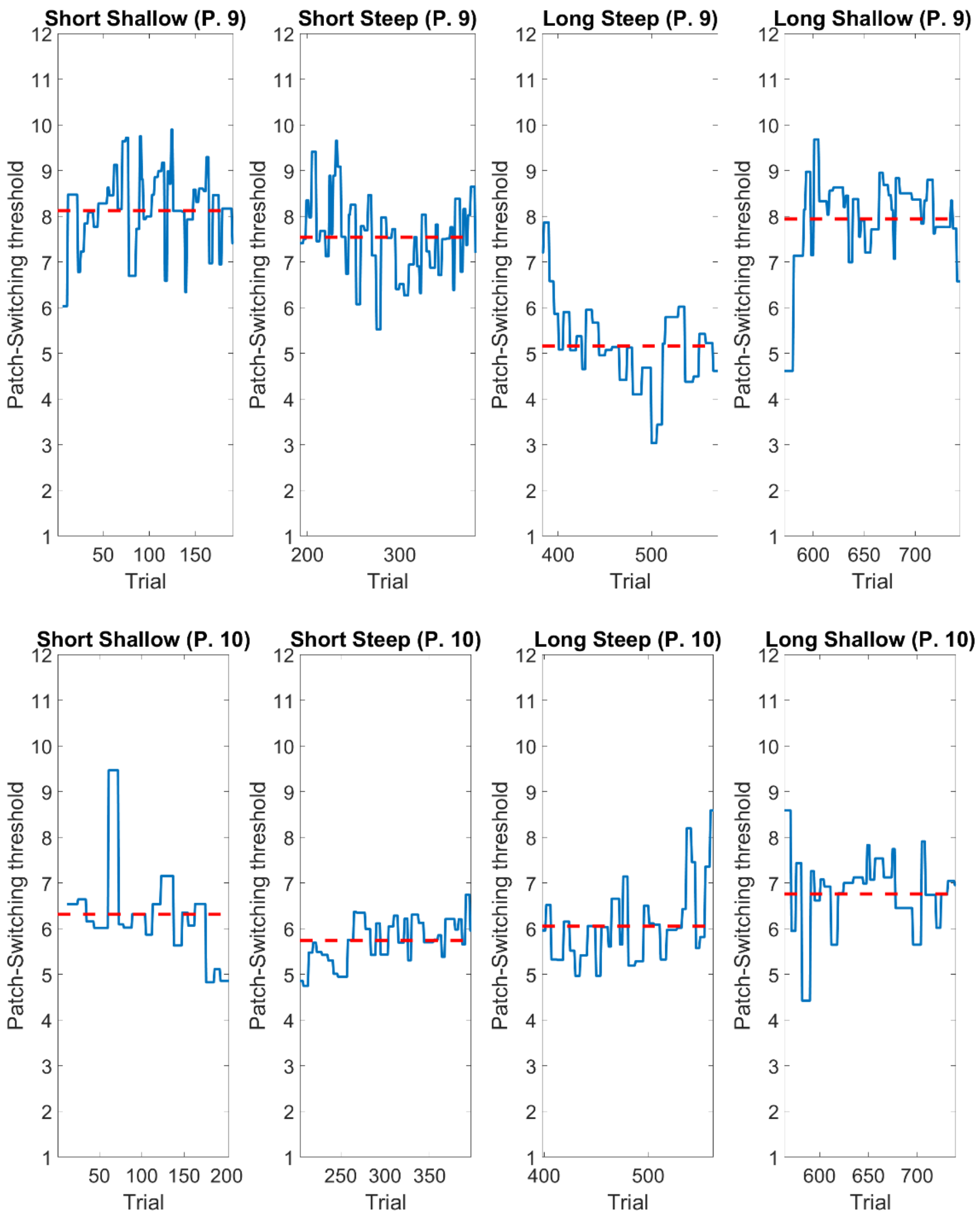
Short Shallow (P. 9)
Short Steep (P. 9)
Long Steep (P. 9)
Long Shallow (P. 9)
Short Shallow (P. 10)
Short Steep (P. 10)
Long Steep (P. 10)
Long Shallow (P. 10)
Patch-Switching threshold
Trial

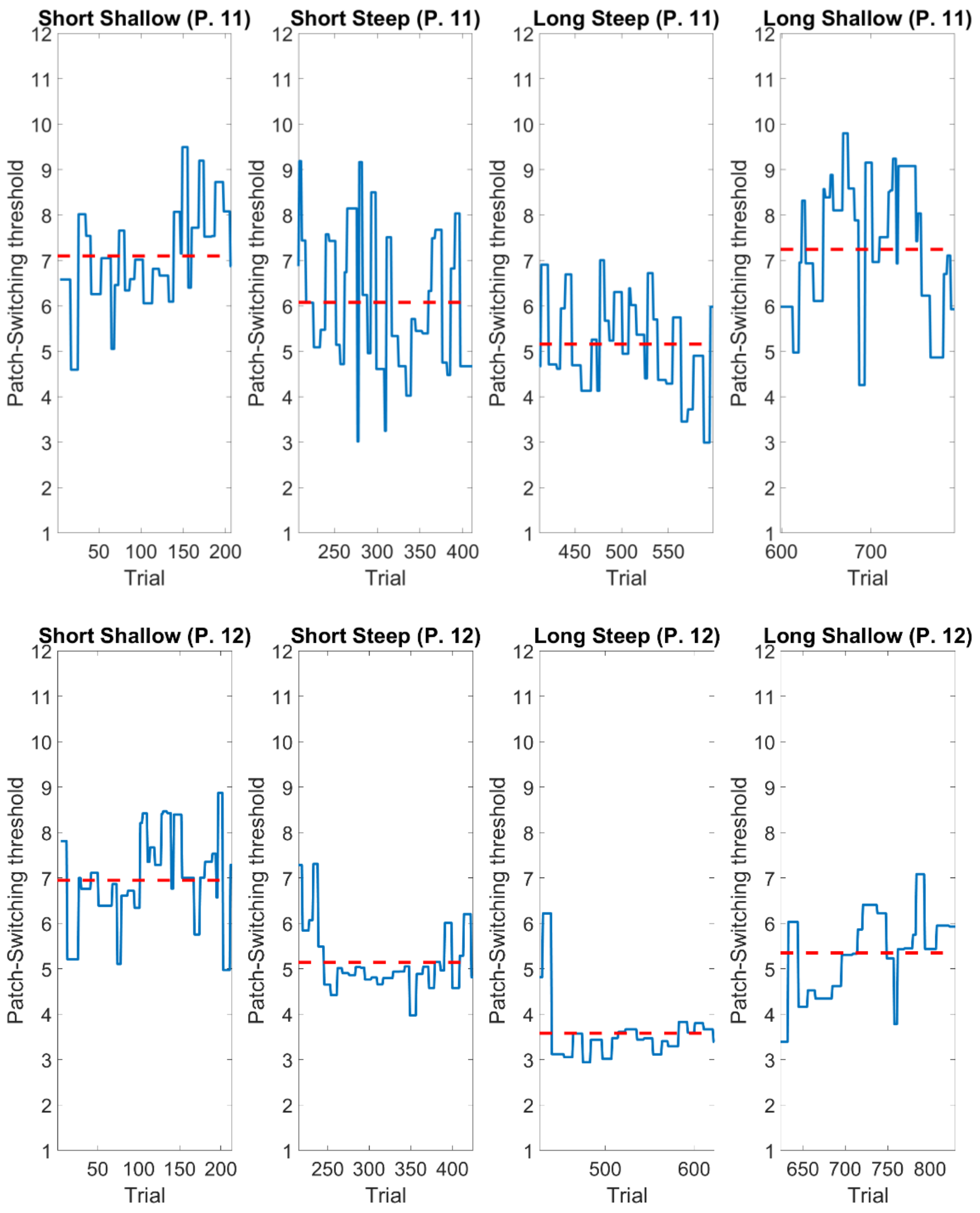

Short Shallow (P. 11)
Short Steep (P. 11)
Long Steep (P. 11)
Long Shallow (P. 11)
Short Shallow (P. 12)
Short Steep (P. 12)
Long Steep (P. 12)
Long Shallow (P. 12)
Patch-Switching threshold
Trial

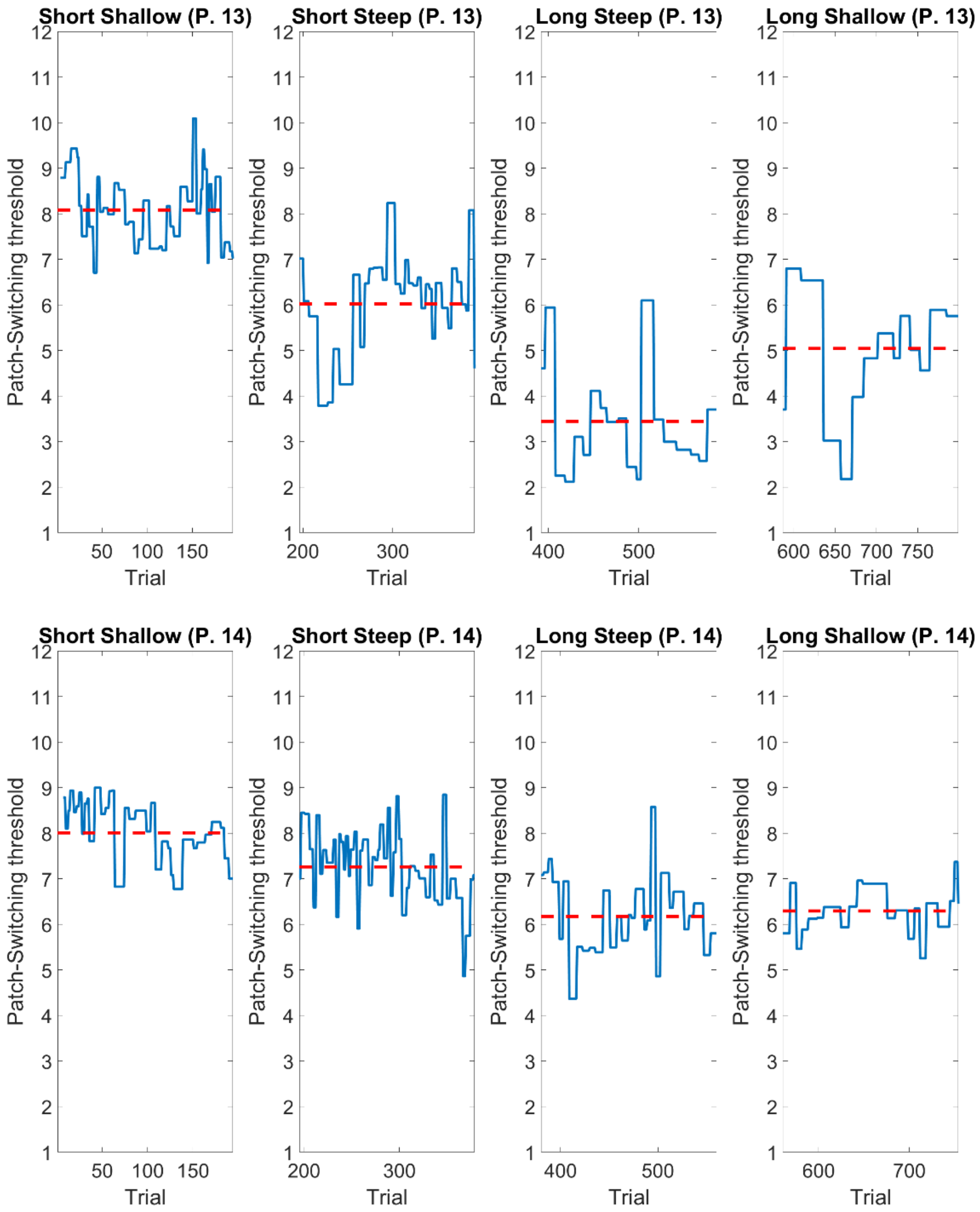

Short Shallow (P. 13)
Short Steep (P. 13)
Long Steep (P. 13)
Long Shallow (P. 13)
Patch-Switching threshold
Trial
Short Shallow (P. 14)
Short Steep (P. 14)
Long Steep (P. 14)
Long Shallow (P. 14)

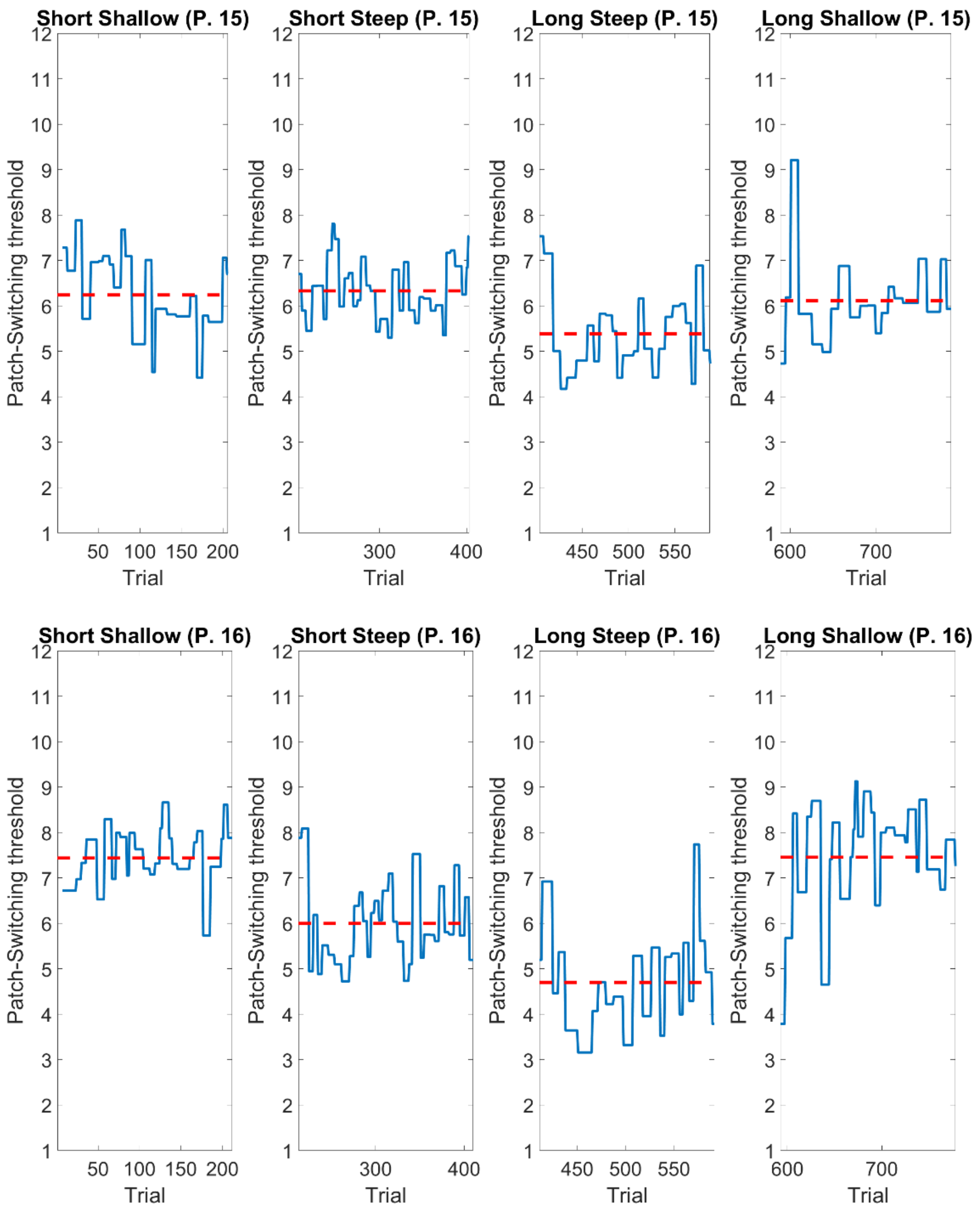

Short Shallow (P. 15)
Short Steep (P. 15)
Long Steep (P. 15)
Long Shallow (P. 15)
Short Shallow (P. 16)
Short Steep (P. 16)
Long Steep (P. 16)
Long Shallow (P. 16)
Patch-Switching threshold
Trial
1 2 3 4 5 6 7 8 9 10 11 12
50 100 150 200
300 400
450 500 550
600 700

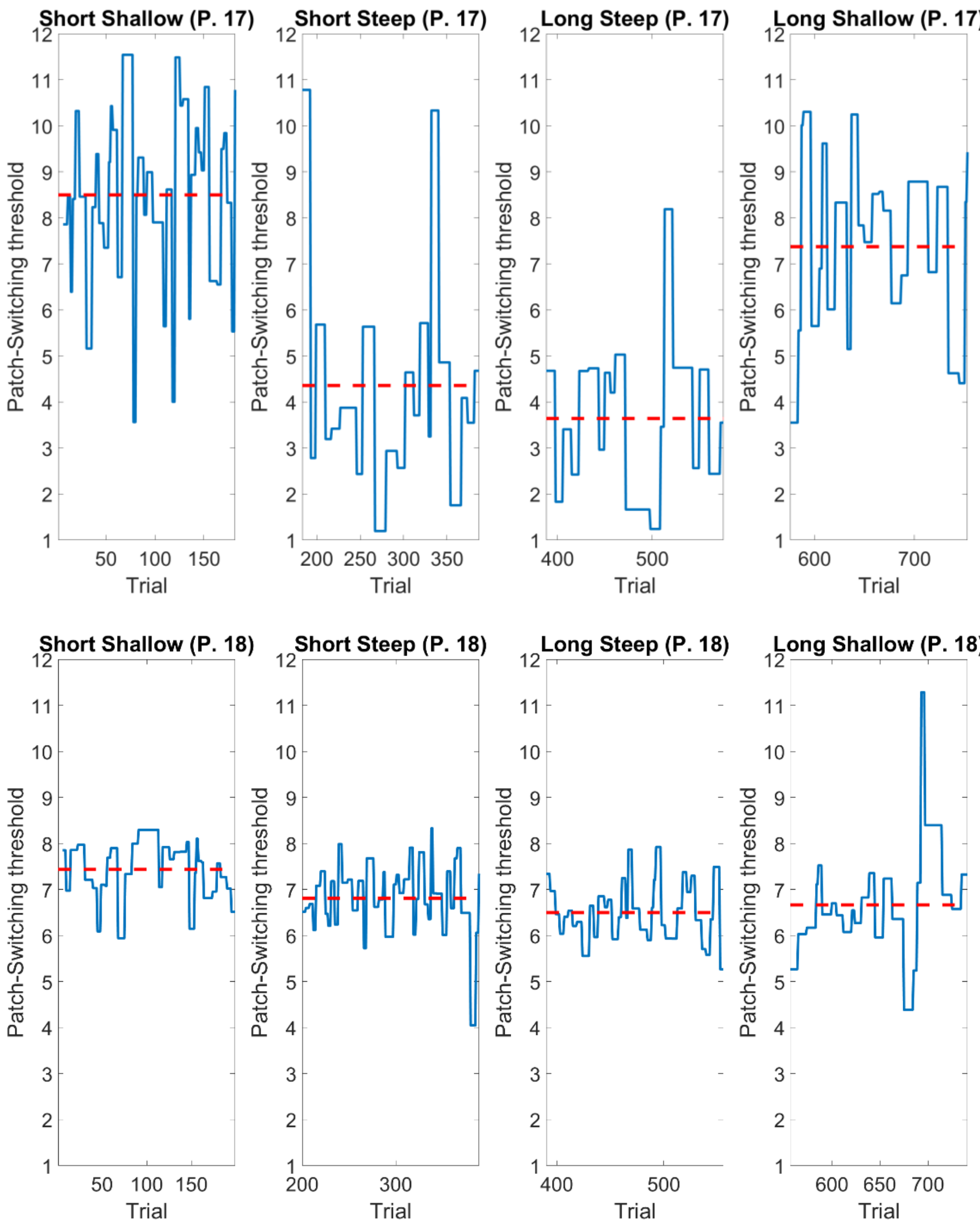

Short Shallow (P. 17)
Short Steep (P. 17)
Long Steep (P. 17)
Long Shallow (P. 17)
Short Shallow (P. 18)
Short Steep (P. 18)
Long Steep (P. 18)
Long Shallow (P. 18)
Patch-Switching threshold
Trial

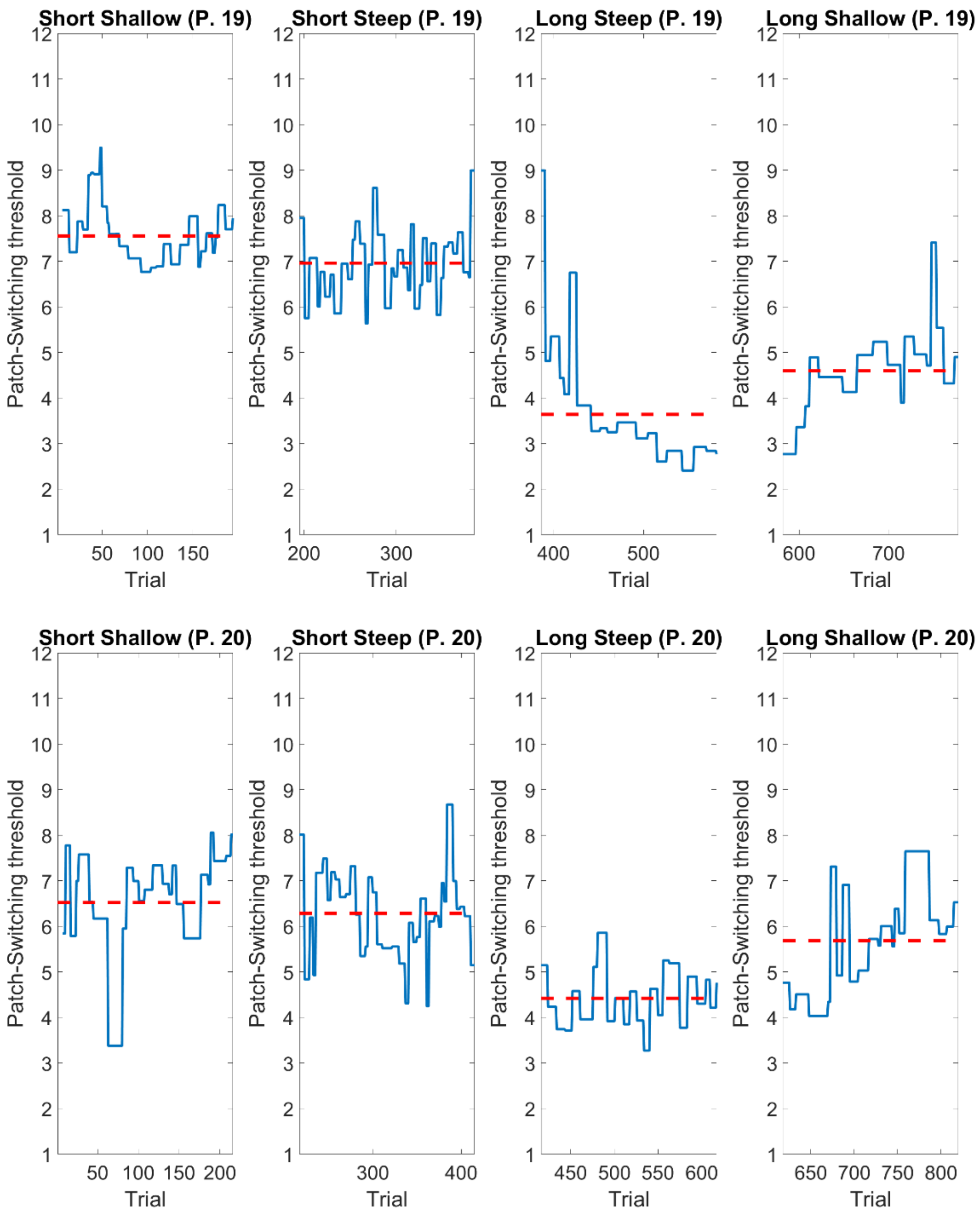

Short Shallow (P. 19)
Short Steep (P. 19)
Long Steep (P. 19)
Long Shallow (P. 19)
Short Shallow (P. 20)
Short Steep (P. 20)
Long Steep (P. 20)
Long Shallow (P. 20)
Patch-Switching threshold
Trial

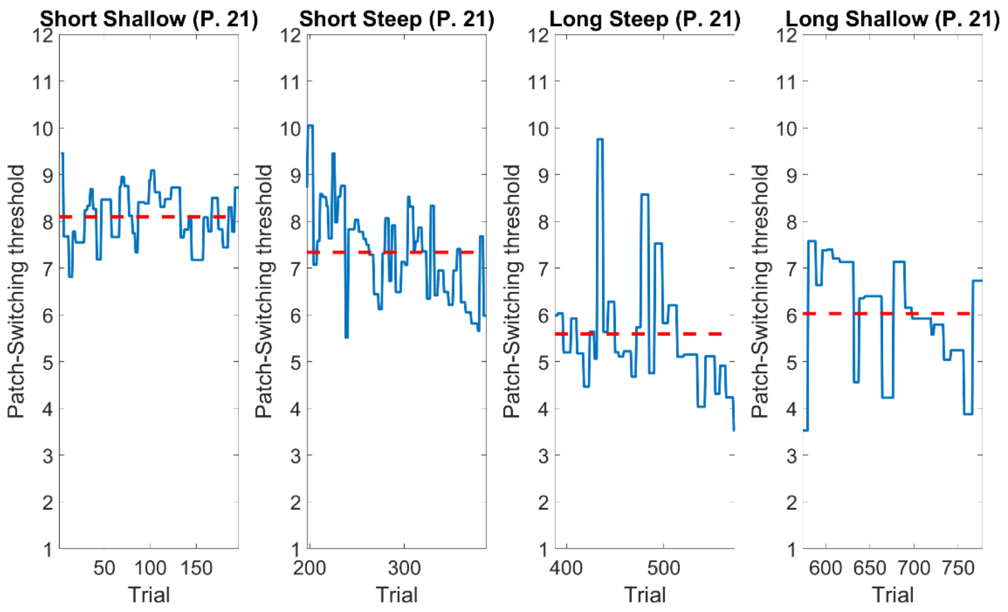

Supporting Information 9. Table depicting % of the trials where the relative forage value (patch-switching threshold/harvest value) in the ith trial was lower (higher or equal) to 1 in each participant separately (and across all participants).

Participant Number	Lower	Higher	Equal
1	82.32258	17.67742	0
2	82.64669	17.35331	0
3	74.44134	25.55866	0
4	80.8642	19.1358	0
5	73.85621	26.14379	0
6	78.99263	21.00737	0
7	83.83961	16.16039	0
8	80.8589	19.1411	0
9	70.23004	29.76996	0
10	75.99451	24.00549	0
11	68.39444	31.60556	0
12	81.477	18.523	0
13	75.12563	24.87437	0
14	74.19786	25.80214	0
15	78.51662	21.48338	0
16	74.6114	25.3886	0
17	58.42246	41.57754	0
18	73.2337	26.7663	0
19	78.52523	21.47477	0
20	77.51843	22.48157	0
21	72.29381	27.70619	0

All	76.017299	23.982701	0

Supporting Information 10. Histograms (for every participant) showing the distribution of relative forage value (patch-switching threshold/harvest value), the dashed red line centred at 1 (x-axis) is the point when the relative forage value is 1, that is the patch-switching threshold and harvest value are equal.

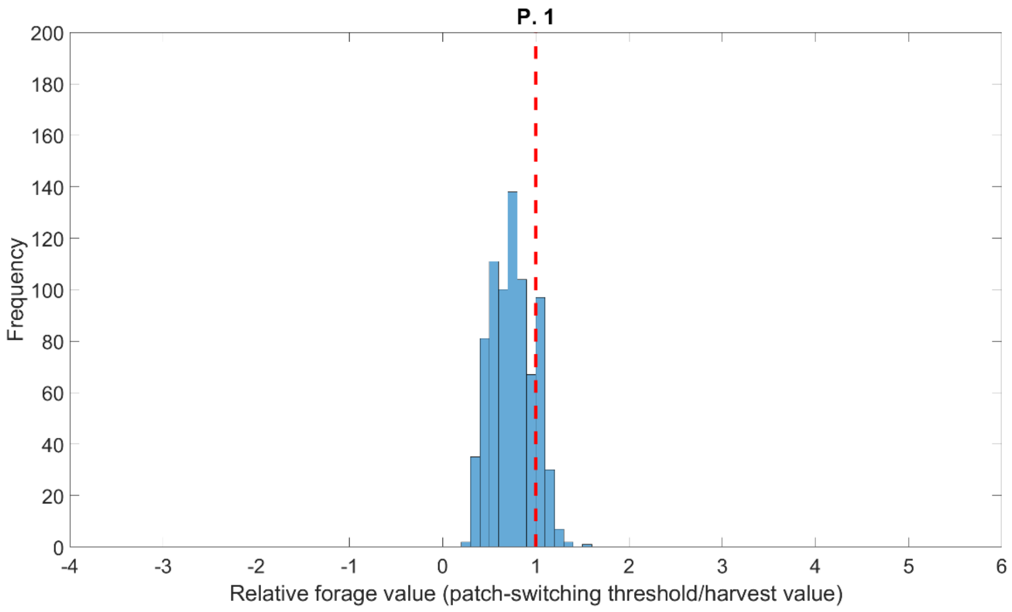

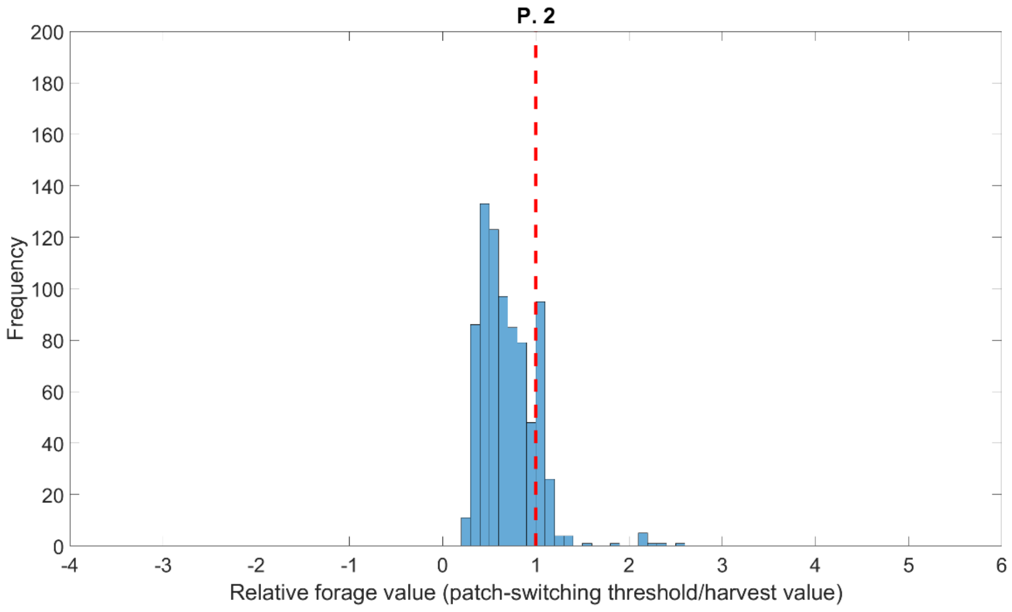

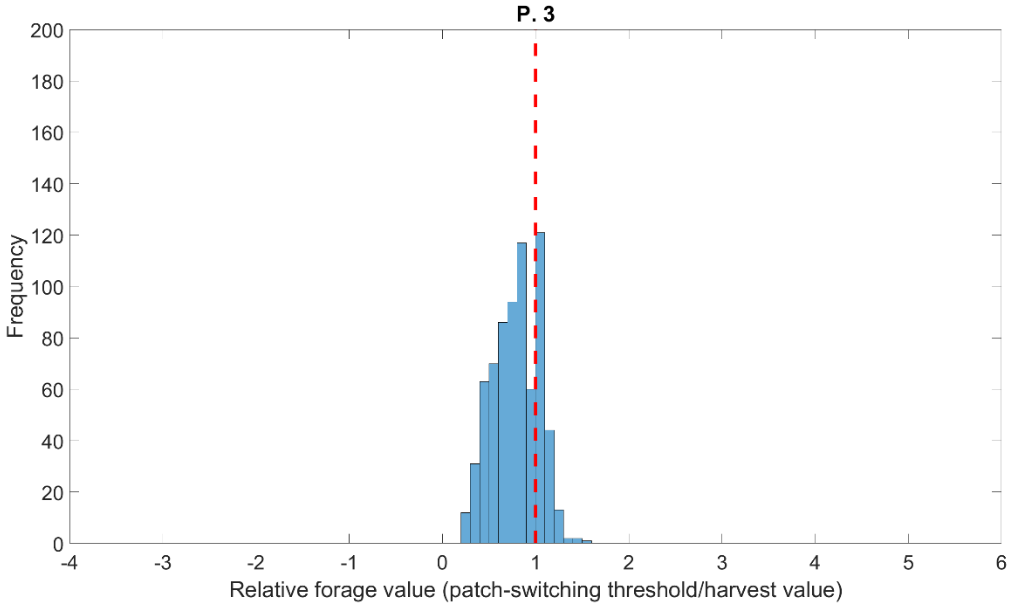
P. 3
Frequency
Relative forage value (patch-switching threshold/harvest value)
200
180
160
140
120
100
80
60
40
20
0
-4
-3
-2
-1
0
1
2
3
4
5
6

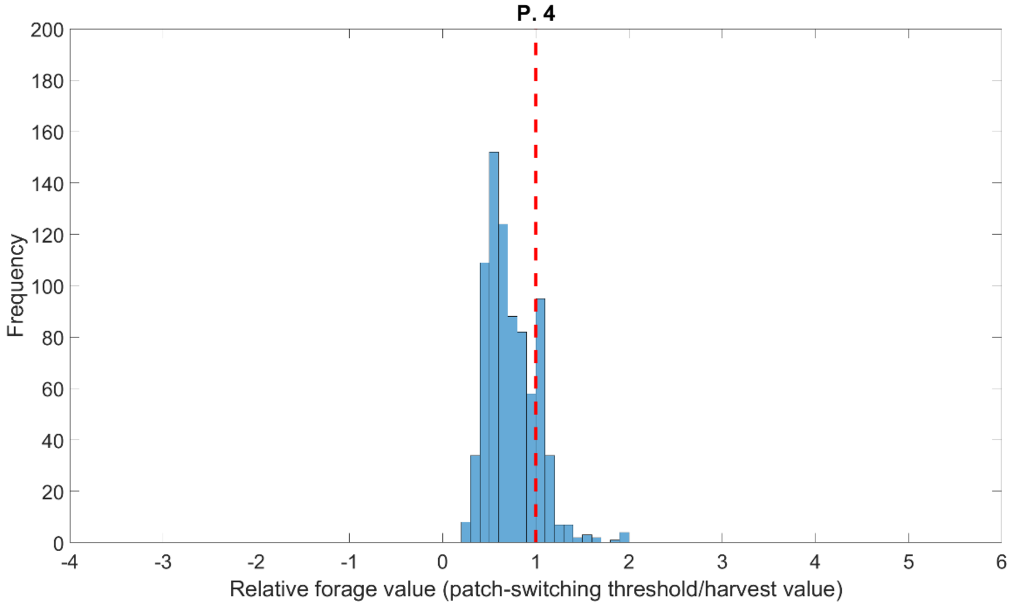
P. 4
Frequency
Relative forage value (patch-switching threshold/harvest value)
200
180
160
140
120
100
80
60
40
20
0
-4
-3
-2
-1
0
1
2
3
4
5
6

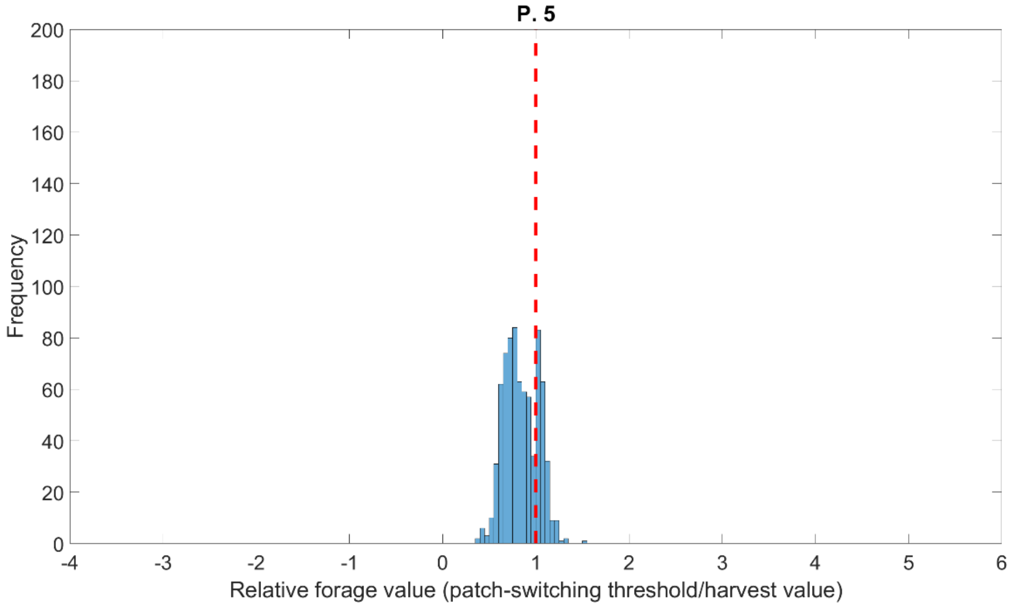
P. 5
Frequency
Relative forage value (patch-switching threshold/harvest value)

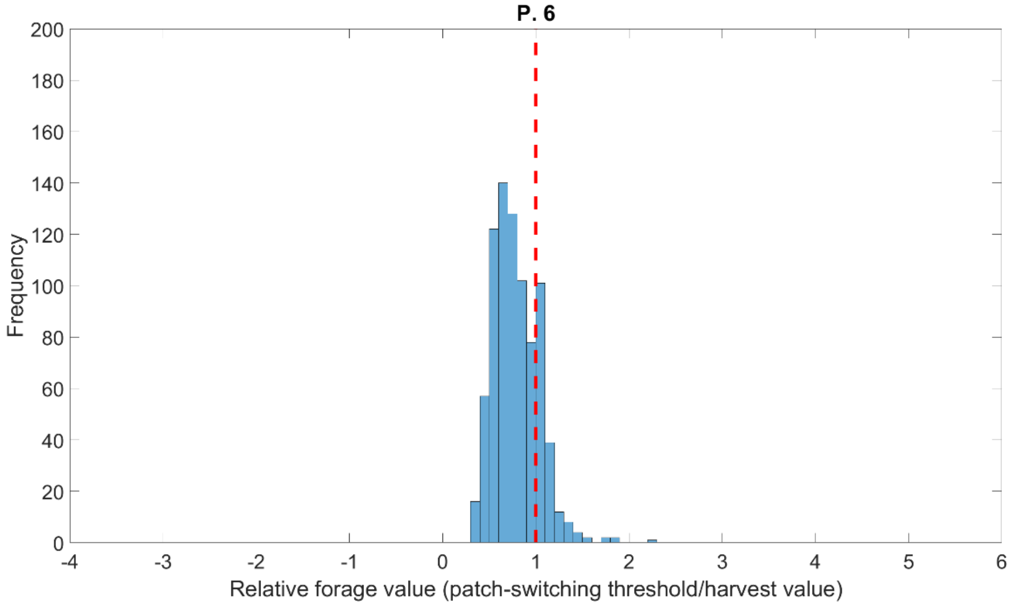
P. 6
Frequency
Relative forage value (patch-switching threshold/harvest value)

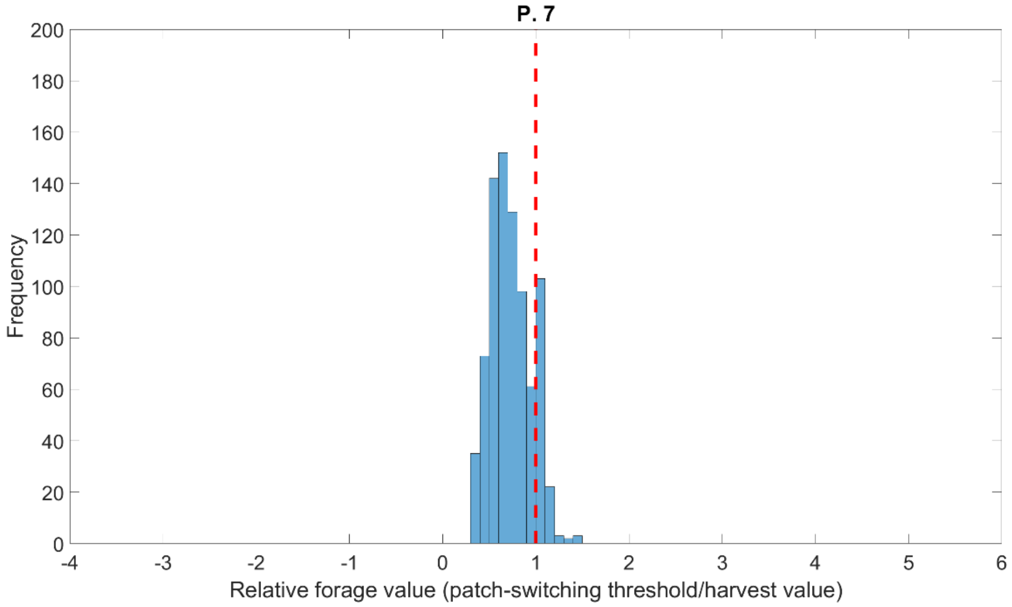
P. 7
200
180
160
140
120
100
80
60
40
20
0
Frequency
-4
-3
-2
-1
0
1
2
3
4
5
6
Relative forage value (patch-switching threshold/harvest value)

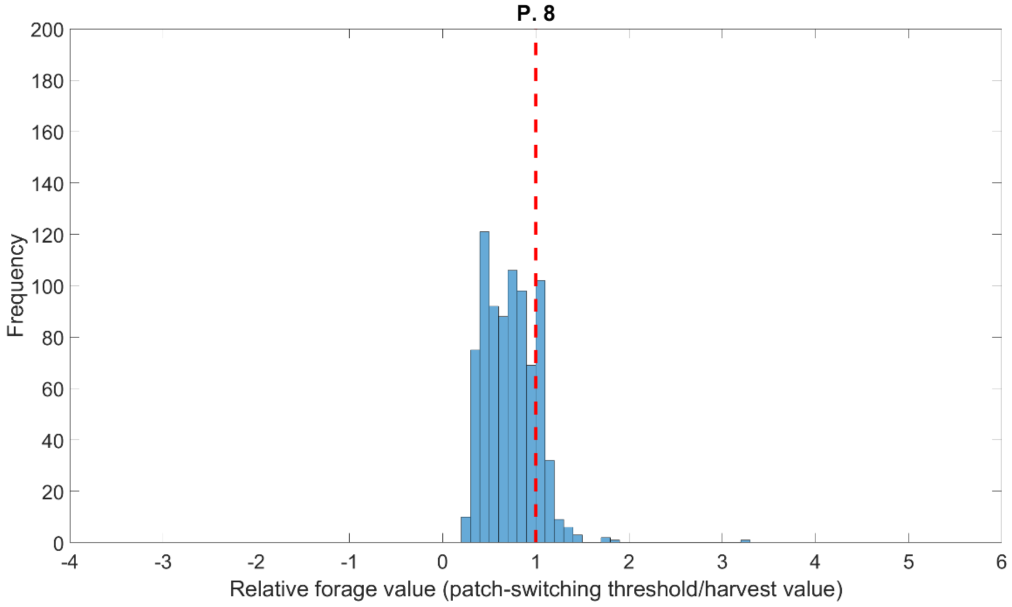
P. 8
200
180
160
140
120
100
80
60
40
20
0
Frequency
-4
-3
-2
-1
0
1
2
3
4
5
6
Relative forage value (patch-switching threshold/harvest value)

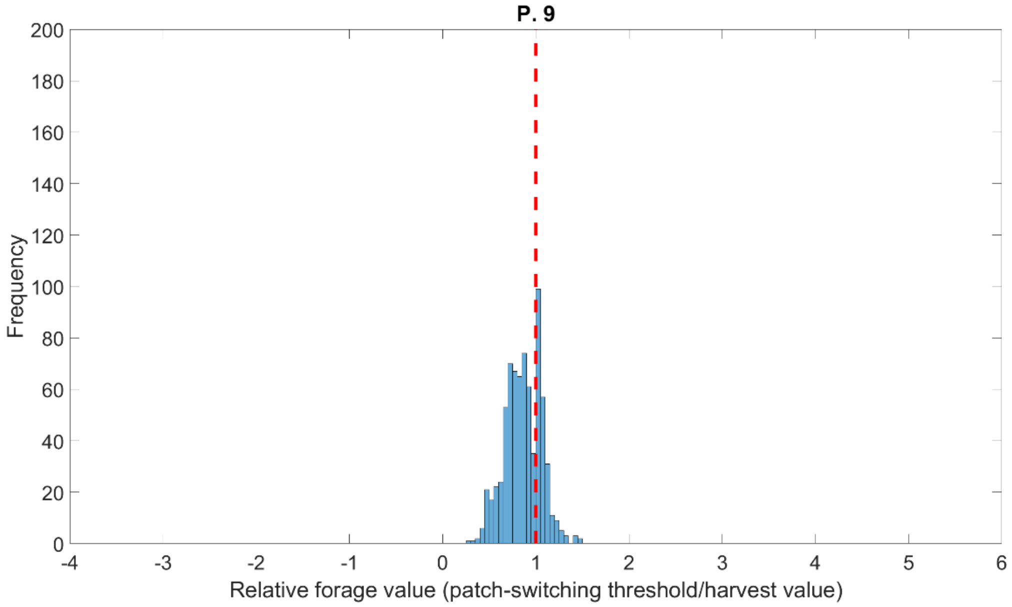
P. 9
Frequency
Relative forage value (patch-switching threshold/harvest value)

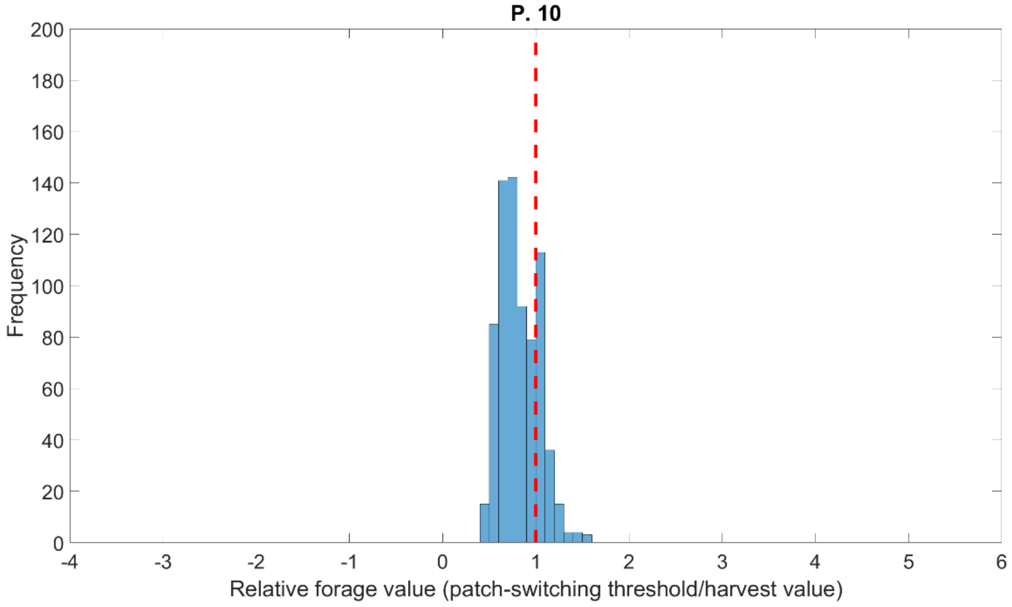
P. 10
Frequency
Relative forage value (patch-switching threshold/harvest value)

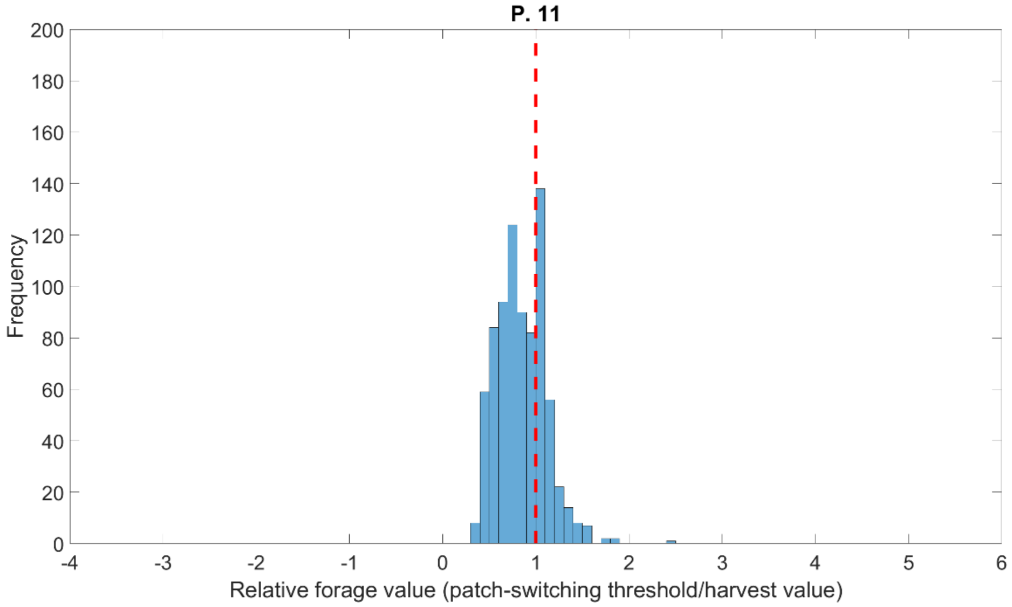
P. 11
200
180
160
140
120
100
80
60
40
20
0
Frequency
-4
-3
-2
-1
0
1
2
3
4
5
6
Relative forage value (patch-switching threshold/harvest value)

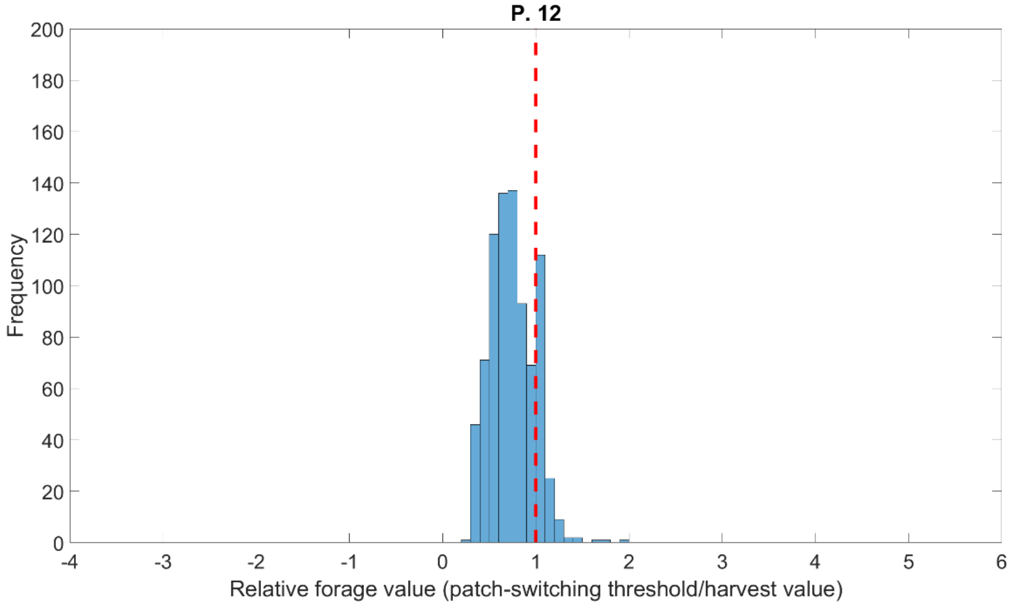
P. 12
200
180
160
140
120
100
80
60
40
20
0
Frequency
-4
-3
-2
-1
0
1
2
3
4
5
6
Relative forage value (patch-switching threshold/harvest value)

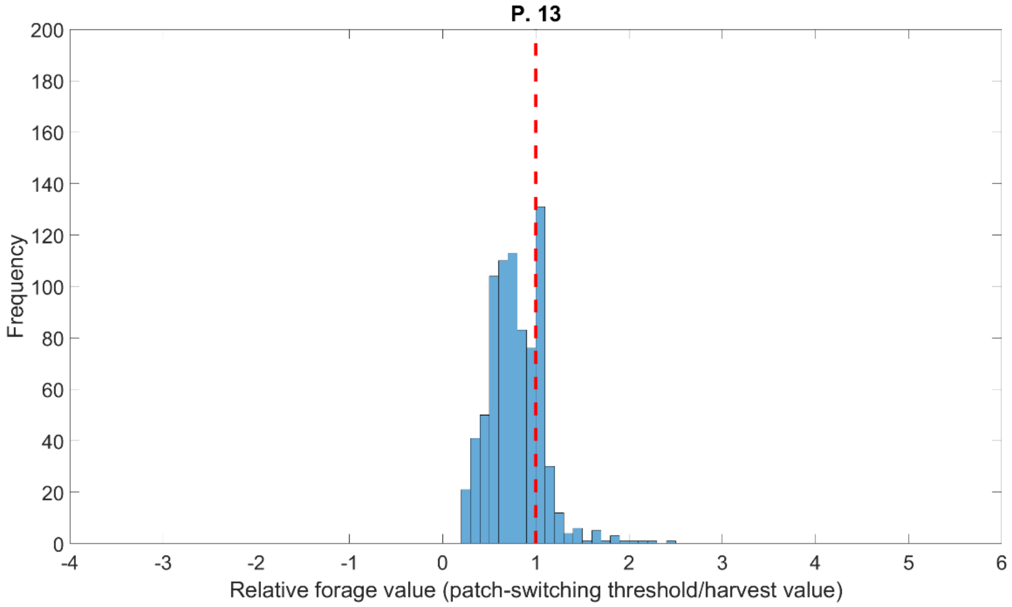
P. 13
Frequency
Relative forage value (patch-switching threshold/harvest value)
200
180
160
140
120
100
80
60
40
20
0
-4
-3
-2
-1
0
1
2
3
4
5
6

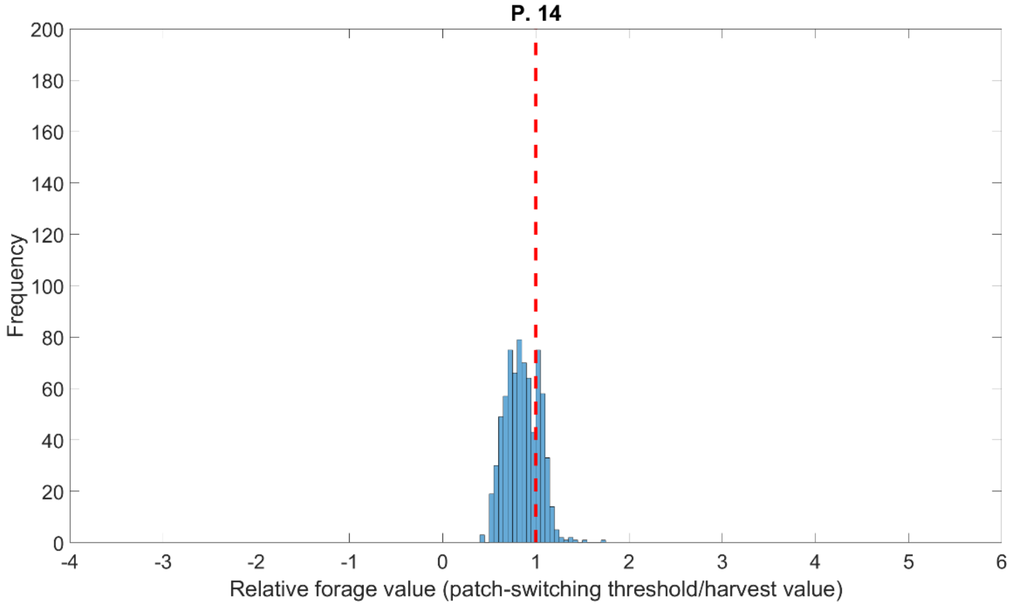
P. 14
Frequency
Relative forage value (patch-switching threshold/harvest value)
200
180
160
140
120
100
80
60
40
20
0
-4
-3
-2
-1
0
1
2
3
4
5
6

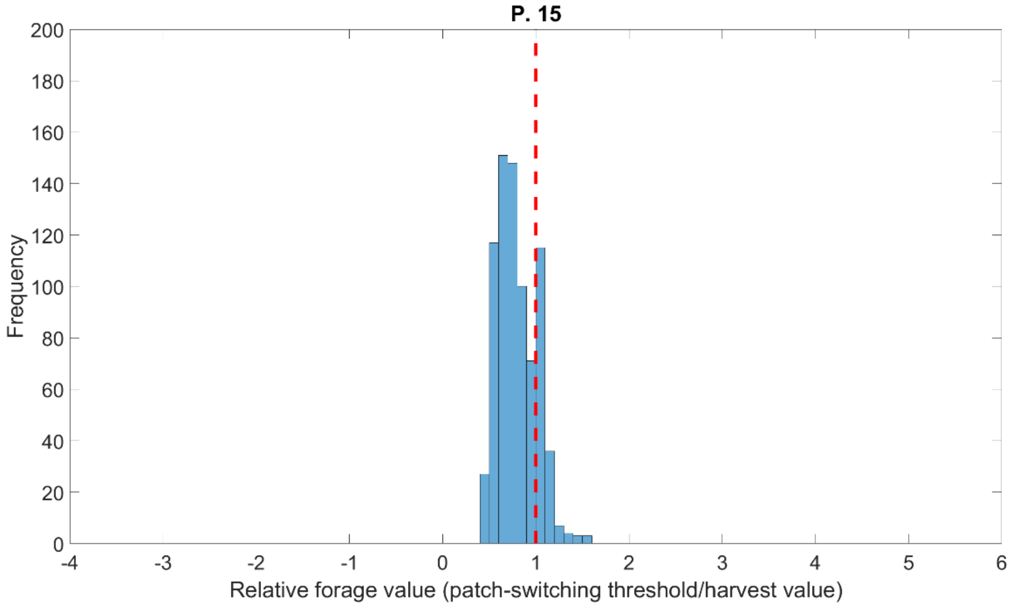
P. 15
Frequency
Relative forage value (patch-switching threshold/harvest value)

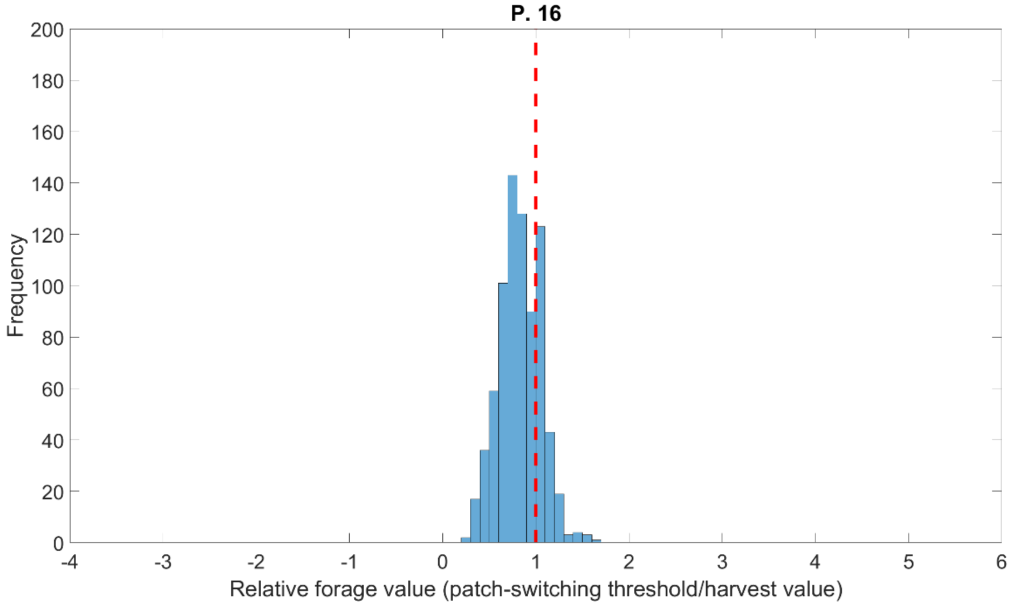
P. 16
Frequency
Relative forage value (patch-switching threshold/harvest value)

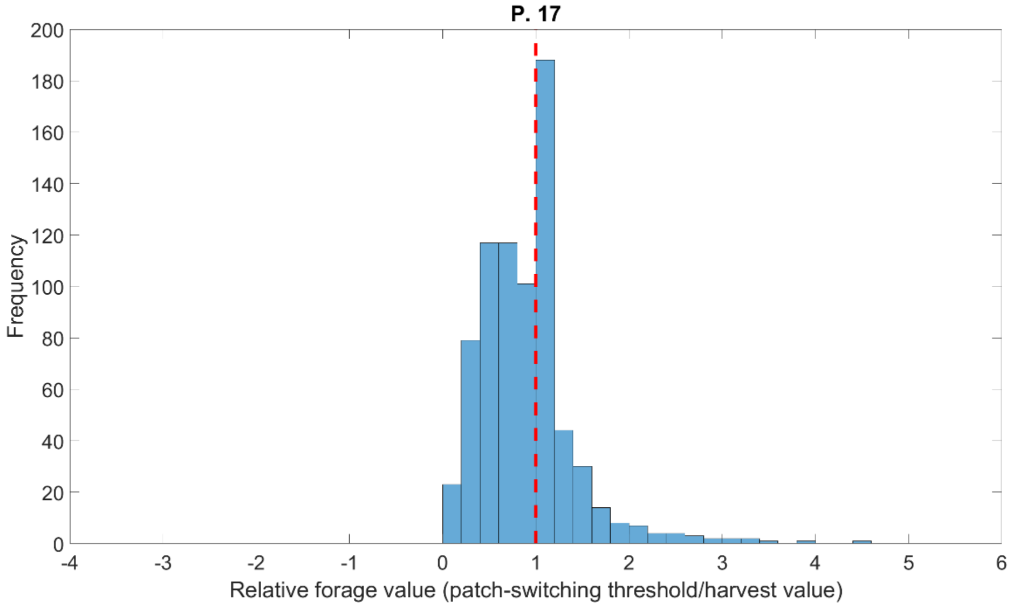
P. 17
Frequency
Relative forage value (patch-switching threshold/harvest value)
200
180
160
140
120
100
80
60
40
20
0
-4
-3
-2
-1
0
1
2
3
4
5
6

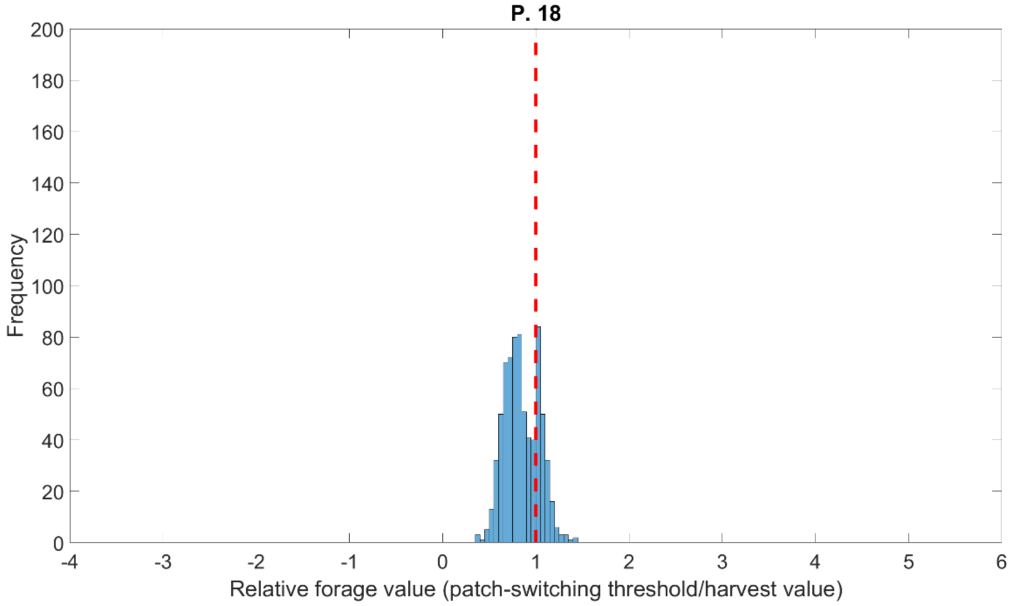
P. 18
Frequency
Relative forage value (patch-switching threshold/harvest value)
200
180
160
140
120
100
80
60
40
20
0
-4
-3
-2
-1
0
1
2
3
4
5
6

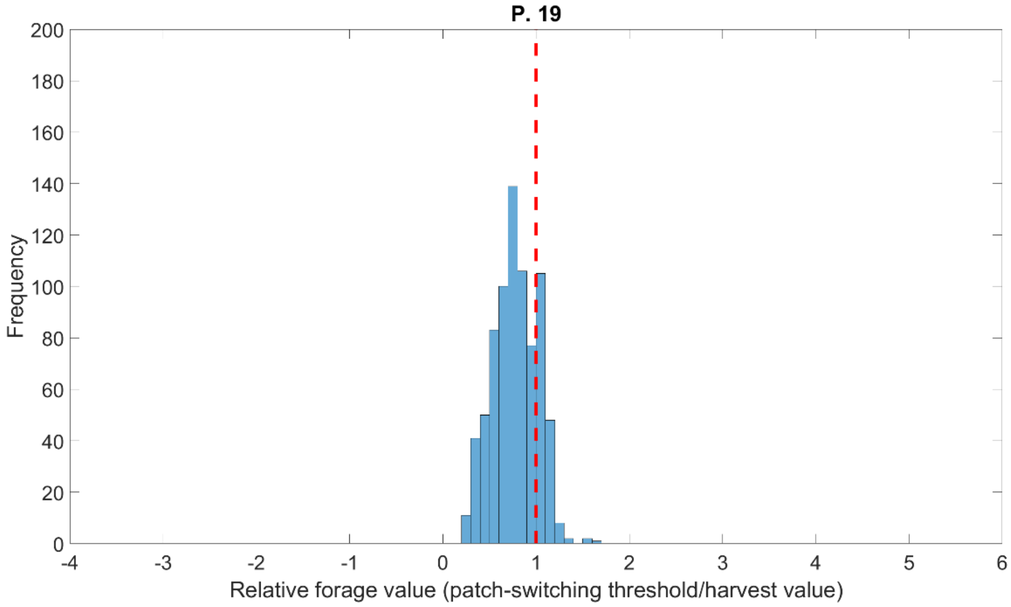
P. 19
Frequency
Relative forage value (patch-switching threshold/harvest value)
200
180
160
140
120
100
80
60
40
20
0
-4
-3
-2
-1
0
1
2
3
4
5
6

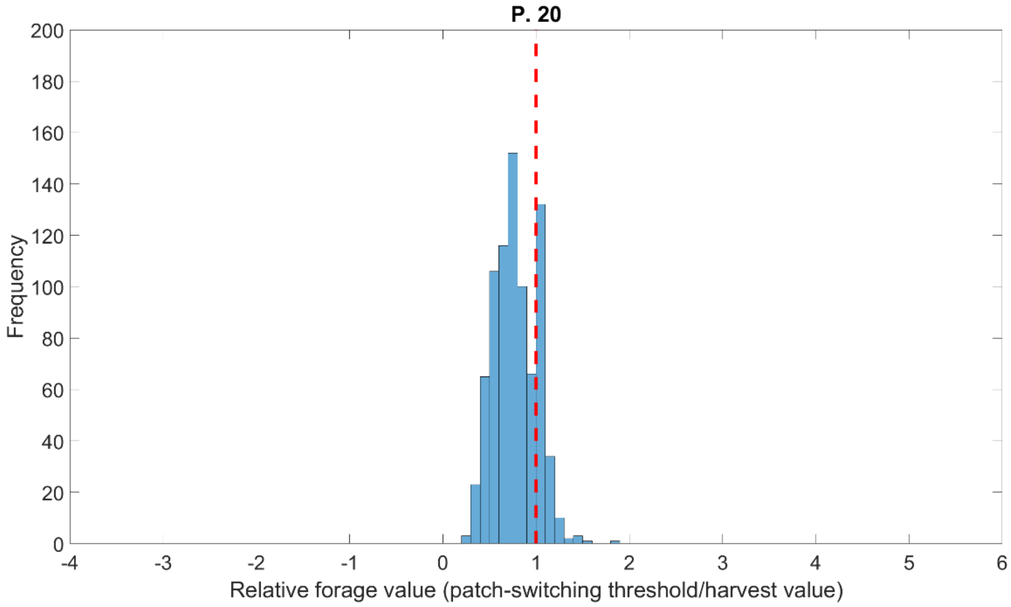
P. 20
Frequency
Relative forage value (patch-switching threshold/harvest value)
200
180
160
140
120
100
80
60
40
20
0
-4
-3
-2
-1
0
1
2
3
4
5
6

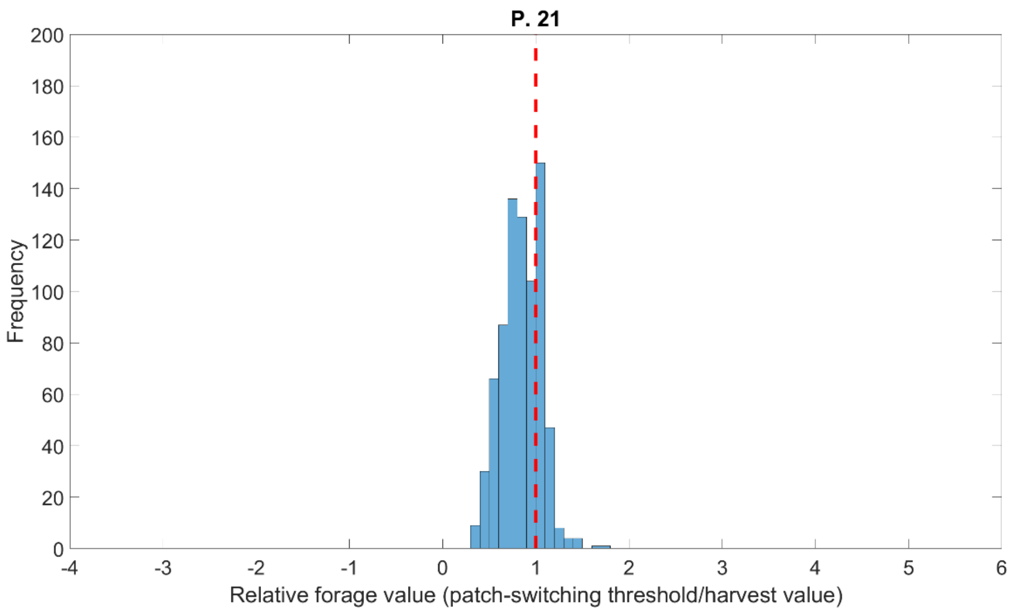

Supporting Information 11.

Correlation coefficients (**Supporting Information 11.1**) between the parametric modulators included in the GLM (collapsing across the personal and social conditions), and plots (**Supporting Information 11.2**) of the regressors (convolved) in every run and in each participant.

Supporting Information 11.1. Correlation coefficients between the (demeaned) parametric modulators (demeaned scores before convolution) included in the GLM. D=decision, HV=harvest value, NP=new patch, PST=patch-switching threshold, RT=reaction time, TTL=travel time learning, RFV=relative forage value.

	**D**	**HV**	**NP**	**PST**	**RT**	**TTL**	**DRL**	**RFV**
**D**	1.00	0.33	-0.26	-0.03	-0.06	0.00	0.08	-0.33
**HV**	0.33	1.00	0.32	0.05	-0.11	-0.02	0.21	-0.83
**NP**	-0.26	0.32	1.00	0.02	0.04	0.00	-0.08	-0.27
**PST**	-0.03	0.05	0.02	1.00	0.05	0.00	0.03	0.43
**RT**	-0.06	-0.11	0.04	0.05	1.00	0.01	-0.07	0.11
**TTL**	0.00	-0.02	0.00	0.00	0.01	1.00	-0.01	0.02
**DRL**	0.08	0.21	-0.08	0.03	-0.07	-0.01	1.00	-0.18
**RFV**	-0.33	-0.83	-0.27	0.43	0.11	0.02	-0.18	1.00

Supporting Information 11.2. Correlation coefficients between the (demeaned) parametric modulators (after HRF convolution) included in the GLM. D=decision, HV=harvest value, NP=new patch, PST=patch-switching threshold, RT=reaction time, TTL=travel time learning, RFV=relative forage value.

	Personal									Charity							
	**D**	**HV**	**NP**	**PST**	**RT**	**TTL**	**DRL**	**RFV**		**D**	**HV**	**NP**	**PST**	**RT**	**TTL**	**DRL**	**RFV**
**D**	1.00	0.40	-0.11	-0.07	-0.23	0.02	0.22	-0.50	**D**	1.00	0.42	-0.09	-0.04	-0.22	0.04	0.22	-0.50
**HV**	0.40	1.00	0.47	0.00	-0.14	-0.01	0.14	-0.76	**HV**	0.42	1.00	0.48	0.06	-0.11	0.00	0.18	-0.77
**NP**	-0.11	0.47	1.00	0.05	0.10	-0.01	-0.15	-0.31	**NP**	-0.09	0.48	1.00	0.04	0.09	-0.02	-0.12	-0.34
**PST**	-0.07	0.00	0.05	1.00	0.04	0.05	0.02	0.35	**PST**	-0.04	0.06	0.04	1.00	0.06	-0.06	0.04	0.30
**RT**	-0.23	-0.14	0.10	0.04	1.00	0.00	-0.11	0.15	**RT**	-0.22	-0.11	0.09	0.06	1.00	-0.01	-0.08	0.13
**TTL**	0.02	-0.01	-0.01	0.05	0.00	1.00	-0.01	0.04	**TTL**	0.04	0.00	-0.02	-0.06	-0.01	1.00	0.01	-0.03
**DRL**	0.22	0.14	-0.15	0.02	-0.11	-0.01	1.00	-0.17	**DRL**	0.22	0.18	-0.12	0.04	-0.08	0.01	1.00	-0.18
**RFV**	-0.50	-0.76	-0.31	0.35	0.15	0.04	-0.17	1.00	**RFV**	-0.50	-0.77	-0.34	0.30	0.13	-0.03	-0.18	1.00

Supporting Information 12. Additional information regarding the Design Matrix.

In the following table (assuming only one run was examined) we showed the order of the predictors in the design matrix (first column), the name of the predictors (second column, these predictors were convolved with the canonical HRF), the type of the predictor (third column), the contrast that was used to define the effect of the parametric modulator patch-switching threshold across the self and charity trials (fourth column), the contrast that was used to define the effect of the interaction of the parametric modulator patch-switching threshold across the self vs charity trials (fifth column). As can be seen, each parametric modulator was a separate regressor in the GLM. The interactions were computed as contrast after the GLM was run. Apart from the 18 predictors described in the table below, the design matrix also featured seven additional columns (6 motion parameters calculated during the realignment+1 global) not shown in the table.

Order	Name	Type	CO	IN
1	Onset times (personal trials only)	Main predictor	0	0
2	Decision (personal trials only)	Parametric modulator	0	0
3	Harvest value (personal trials only)	Parametric modulator	0	0
4	New patch (personal trials only)	Parametric modulator	0	0
5	Patch-switching threshold (personal trials only)	Parametric modulator	.5	1
6	Reaction time (personal trials only)	Parametric modulator	0	0
7	Travel time learning (personal trials only)	Parametric modulator	0	0
8	Depletion rate learning (personal trials only)	Parametric modulator	0	0
9	Relative forage value (personal trials only)	Parametric modulator	0	0
10	Onset times (charity trials only)	Main predictor	0	0
11	Decision (charity trials only)	Parametric modulator	0	0
12	Harvest value (charity trials only)	Parametric modulator	0	0
13	New patch (charity trials only)	Parametric modulator	0	0
14	Patch-switching threshold (charity trials only)	Parametric modulator	.5	-1
15	Reaction time (charity trials only)	Parametric modulator	0	0
16	Travel time learning (charity trials only)	Parametric modulator	0	0
17	Depletion rate learning (charity trials only)	Parametric modulator	0	0
18	Relative forage value (charity trials only)	Parametric modulator	0	0

Below there is an example of the GLM from one participant featuring four functional runs.

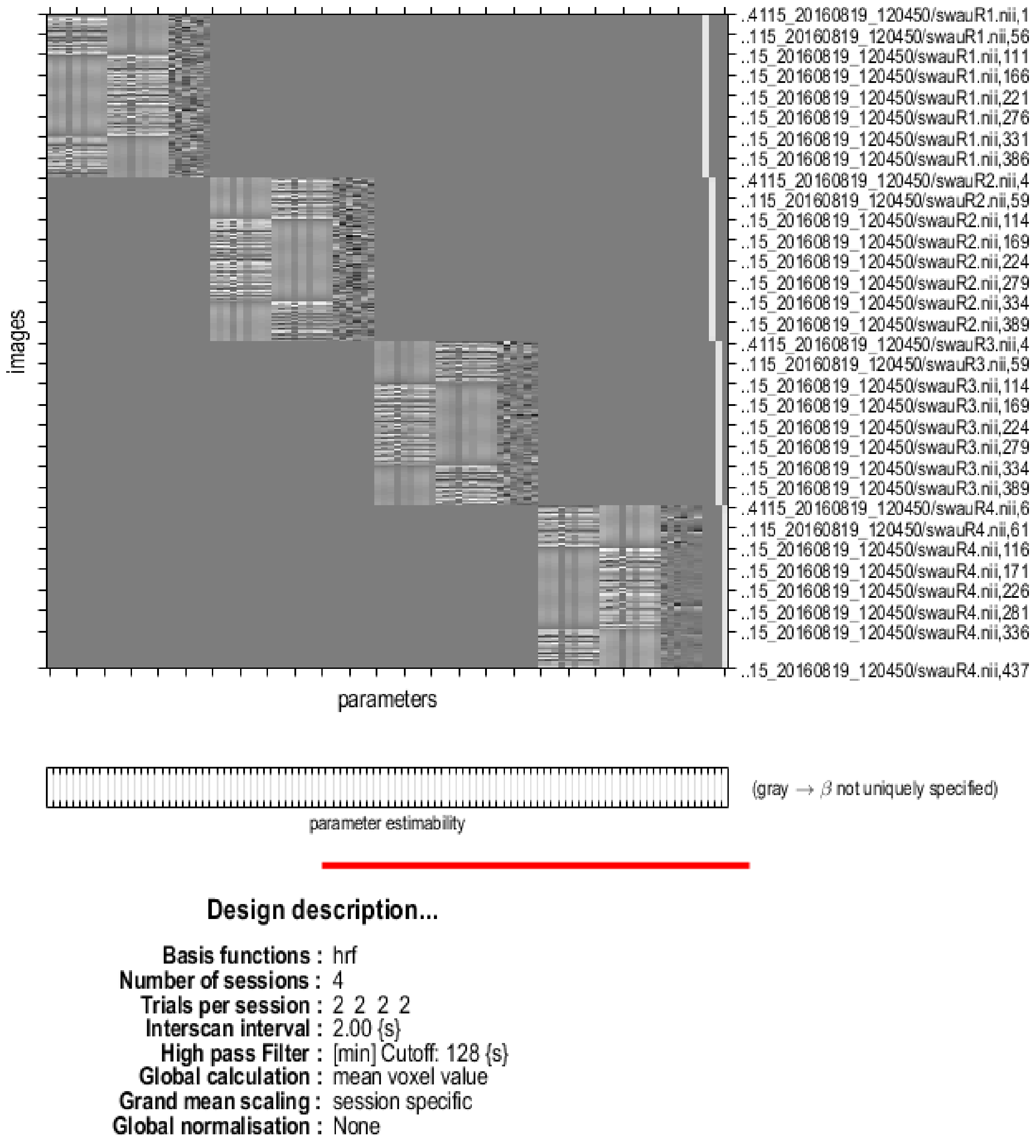

Supporting Information 13. Control analyses where demeaned (per run) trial-wise numerical values for decision, harvest value, new-patch, patch-switching threshold, travel time learning, depletion rate learning, and relative forage value were entered into the GLM. Lateral and medial view imaging results in response to the parametric modulator of decision-positive association (**A**), decision- negative association (**B**), harvest value- positive association (**C**), new patch- negative association (**D**), patch-switching threshold- negative association, (**E**) relative forage value- positive association (**F**).

A) Decision (Positive association)

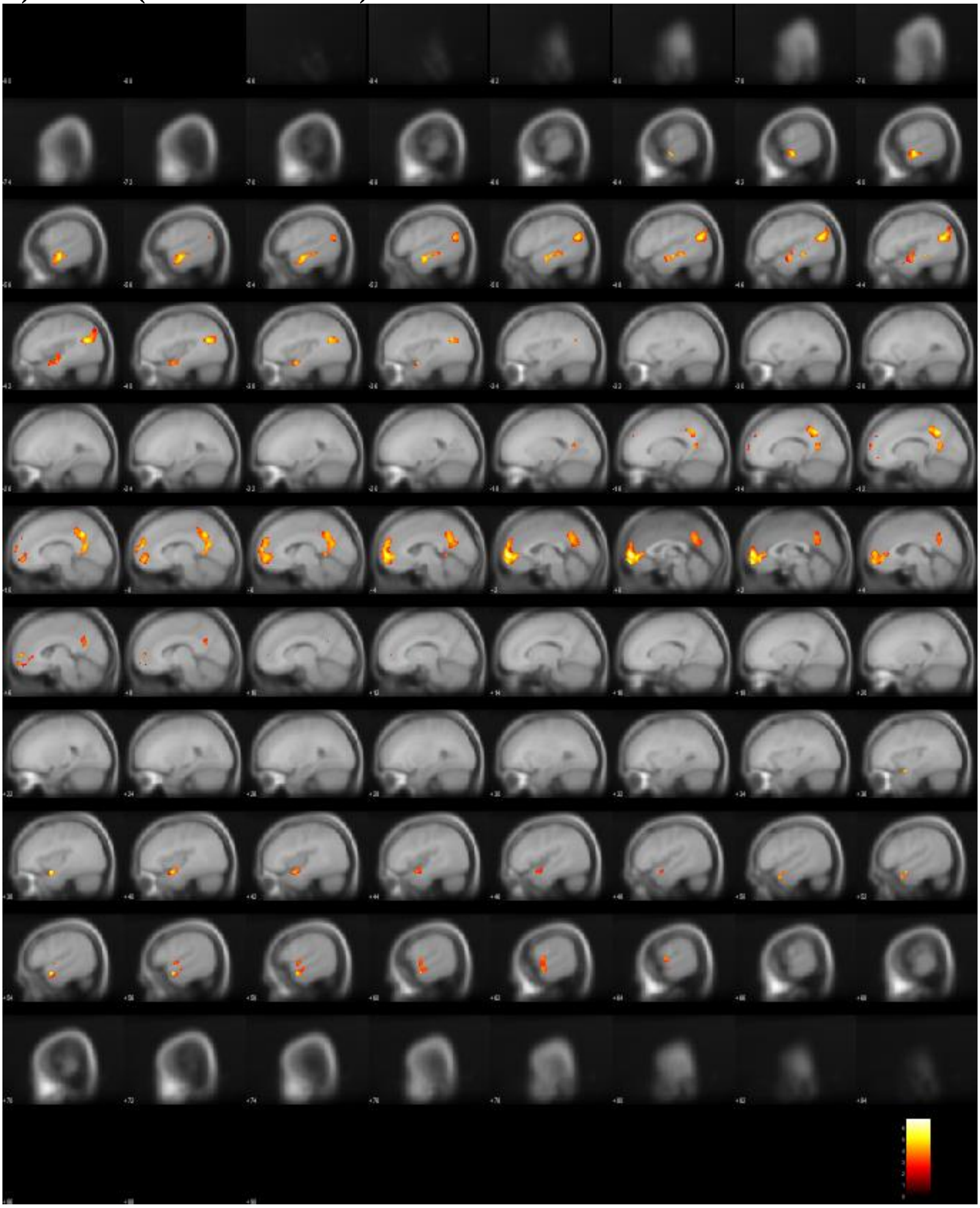

B) Decision (Negative association)

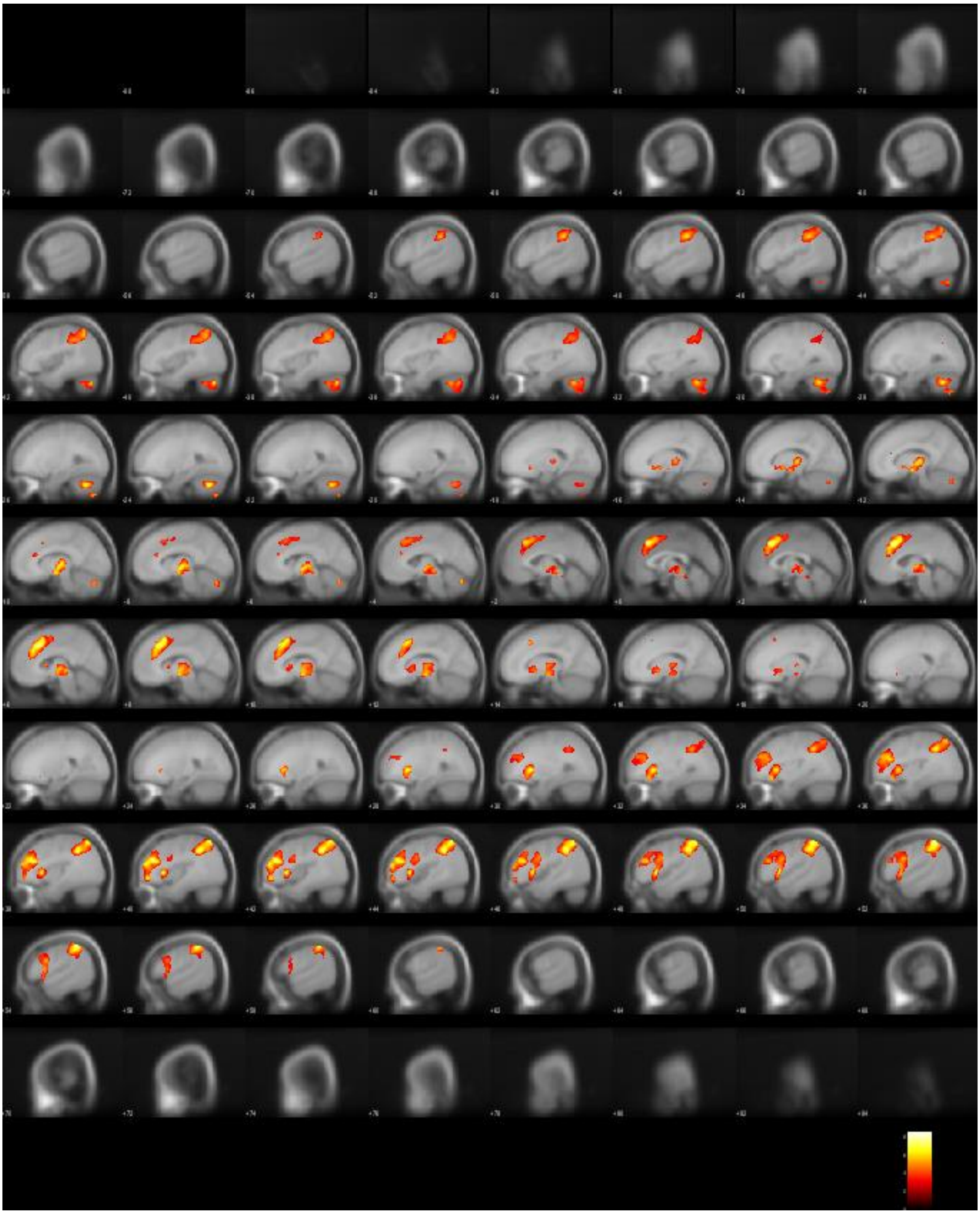

C) Harvest value (Positive association)

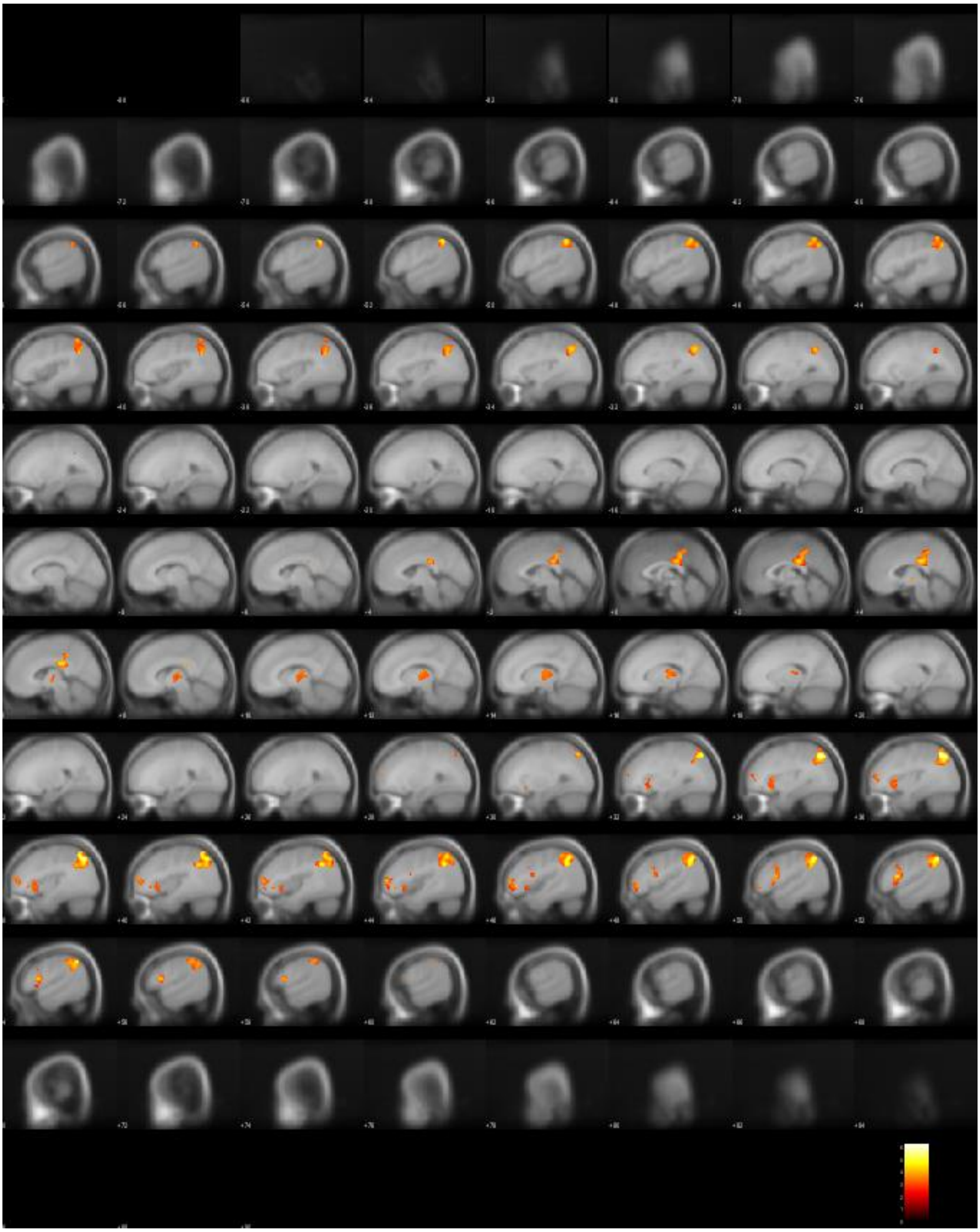

D) New patch (Negative association)

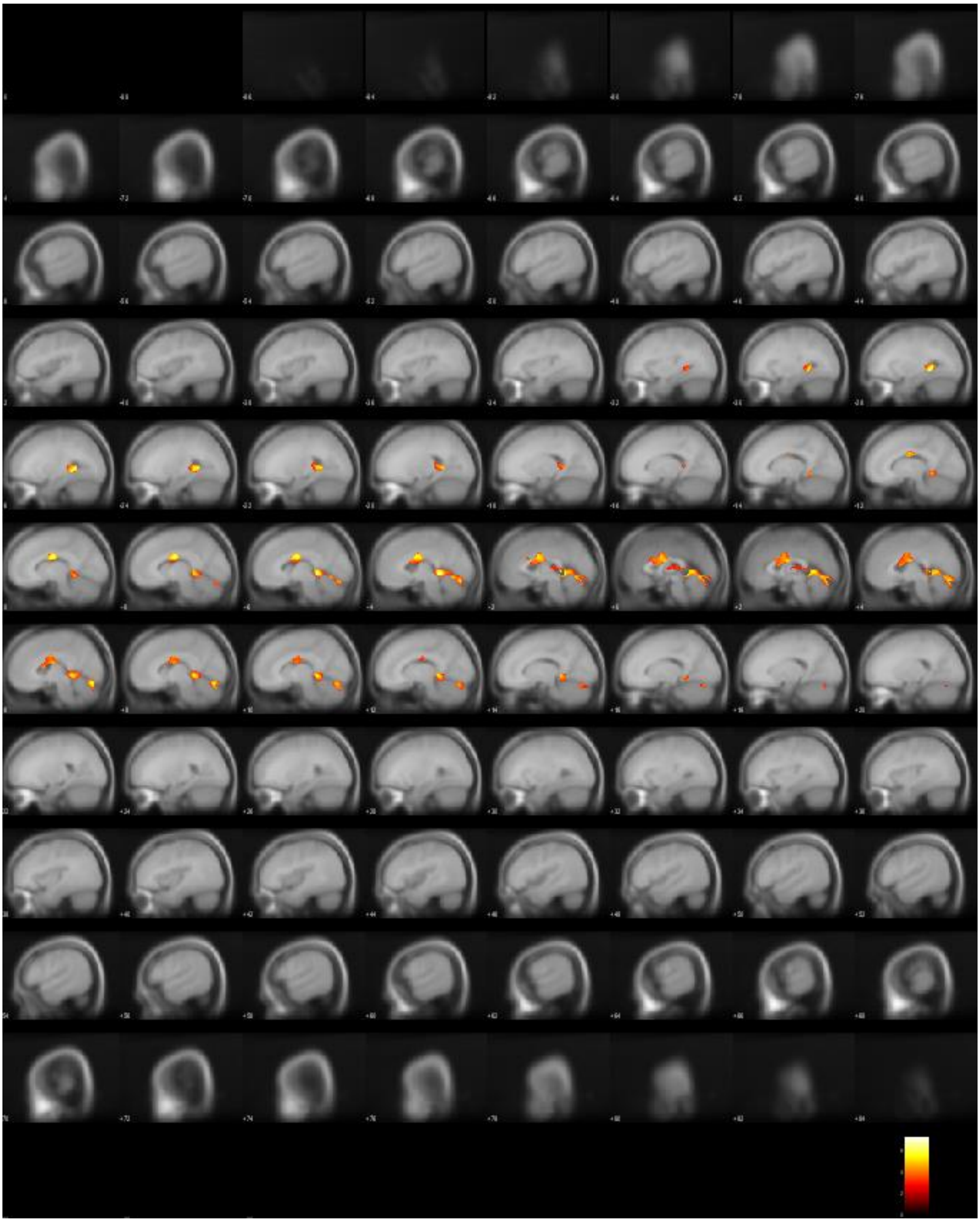

E) Patch-switching threshold (Negative association)

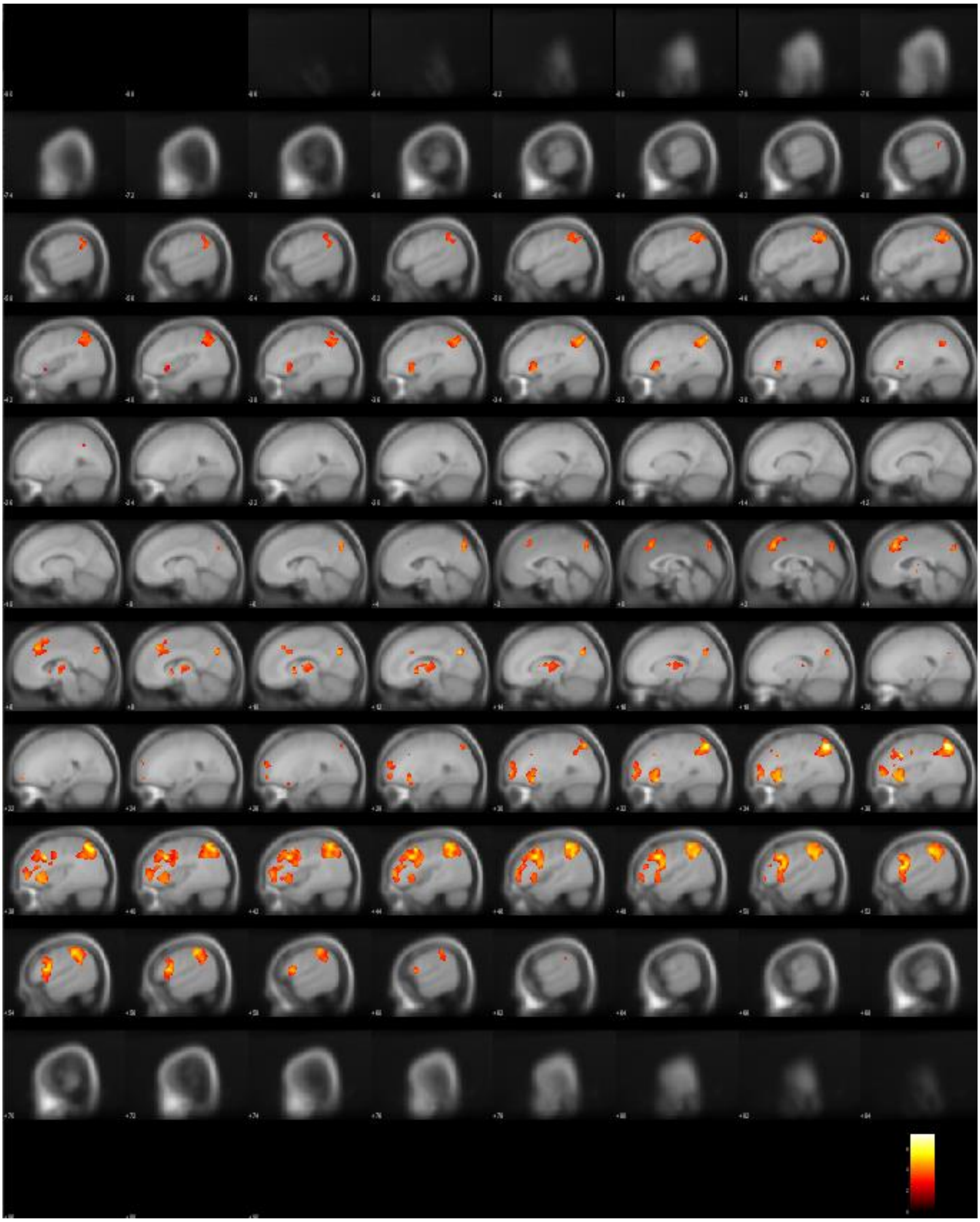

F) Relative forage value (Positive association)

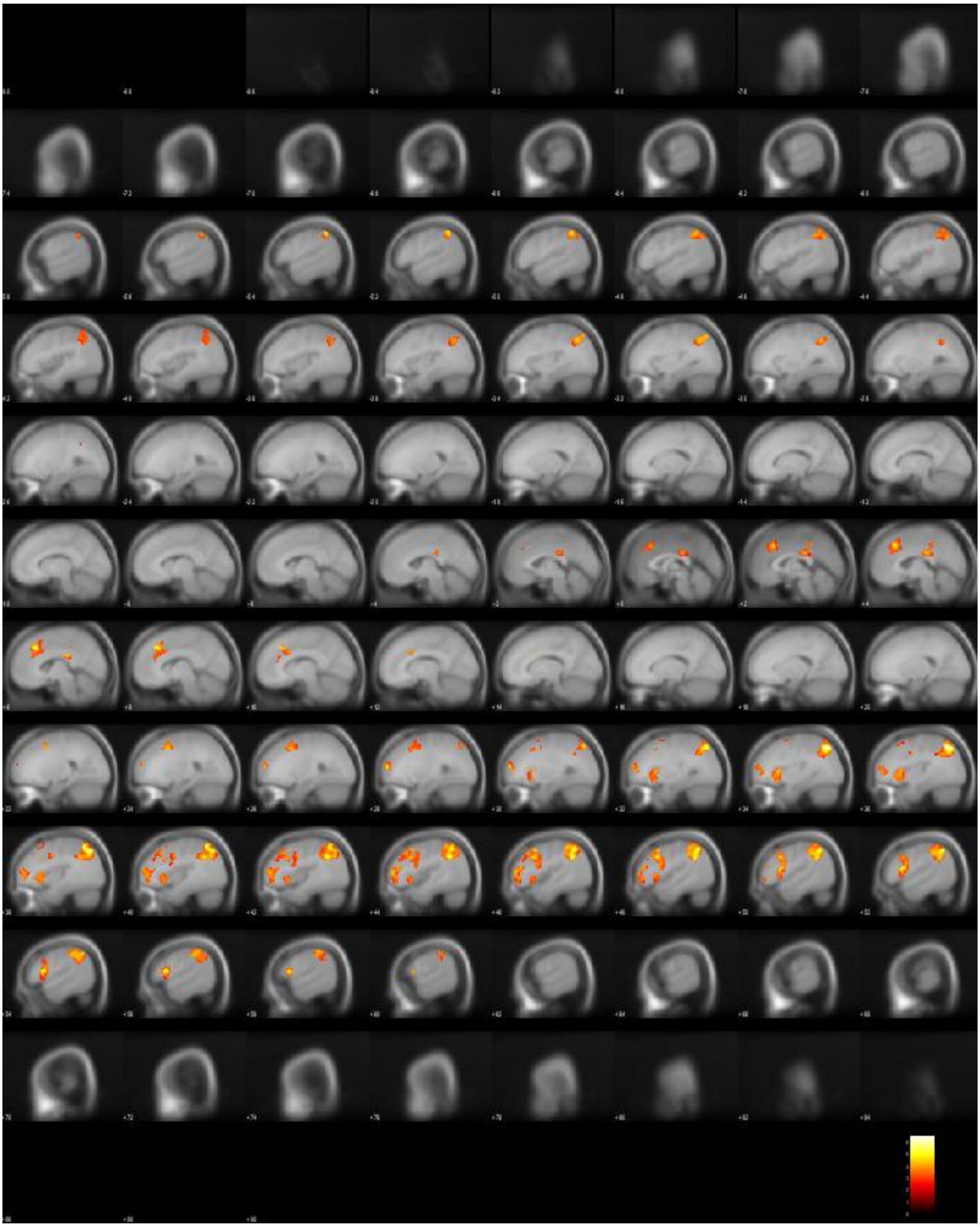

Supporting Information 14. Additional analyses examining the effect of the manipulation on the number of harvest decisions for every tree (before exiting). First, we calculated the number of harvest decisions for every tree (before exiting), then we computed the mean of this variable in each of the 2*2*2 conditions separately and subsequently performed a 2*2*2 ANOVA. The results mirror the corresponding ones obtained using the patch-switching threshold reported in the initial manuscript. Specifically, travel time ($F(1,16)=28.86$, $p<.001$)

and depletion rate (F(1,16)=12.79, p=.003) were statistically significant in predicting the aforementioned dependent variable and as found previously neither source (F(1,16)=.172, p=.684) nor any of the interactions were significant (source*travel time: F(1,16)=1.57, p=.228, source*depletion rate: F(1,16)=.161, p=.693, travel time*depletion rate: F(1,16)=2.84, p=.111, source*travel time*depletion rate: F(1,16)=1.36, p=.261).

Supporting Information 15. Additional analyses examining whether the variability in the patch-switching threshold significantly reduced with time, (i) for every participant individually, we (temporally) segmented each orchard into two segments, the first half and the second half and we calculated the standard deviation of the patch-switching threshold in each of the two segments separately, yielding 8 standard deviation scores for each participant (i.e., 2 segments*4 environment types), (ii) we calculate the average standard deviation across the four environments for each segment separately yielding 2 standard deviation scores for each participant (i.e., 2 segments), (iii) we performed a paired-sample t-test to assess whether there was a reduction in the standard deviation scores as expected, (iv) we found that to be the case where the mean standard deviation of the first segment (M=.97) was significantly higher than the standard deviation of the second segment (M=.82), t(16)=3.45, P=.003, two-tailed). This finding provides additional evidence that our patch-switching measure is an adequate measure to capture learning-induced changes.

Supporting Information 16. Additional results of two simpler GLMs. We conducted two simpler additional GLMs, (i) repeating the original GLM but excluding the predictor relative forage value, which we term "GLM#2 excluding relative forage value", and (ii) repeating the original GLM but excluding the predictor harvest value, which we term "GLM#3 excluding harvest value". The "GLM#2 excluding relative forage value" yielded significant activation for the predictor harvest value, both negative and positive, as can be seen in the table and screenshots below. The "GLM#3 excluding harvest value" yielded significant negative activation for the predictor patch-switching threshold and produced significant positive activation for the predictor relative forage value, mirroring the results of the original GLM.

Positive (+) or negative (-) association	pFWE	k	x	y	z
**GLM#3 excluding harvest value**					
Patch-switching threshold					
-	0.037	493	6	10	50
-	0.012	1208	46	6	22
-	0.028	580	54	-36	48
-	0.048	372	18	-6	60
Relative Forage Value					
+	0.005	973	32	40	30
+	0.005	1054	10	22	32
+	0.042	328	42	6	26
+	0.009	859	48	-38	44
**GLM#2 excluding relative forage value**					
Harvest Value					
-	0.037	330	32	40	30
-	0.015	526	8	14	44

+	0.037	283	-44	-16	10
+	0.014	692	-4	-26	40

GLM#2 excluding relative forage value- Positive association for harvest value

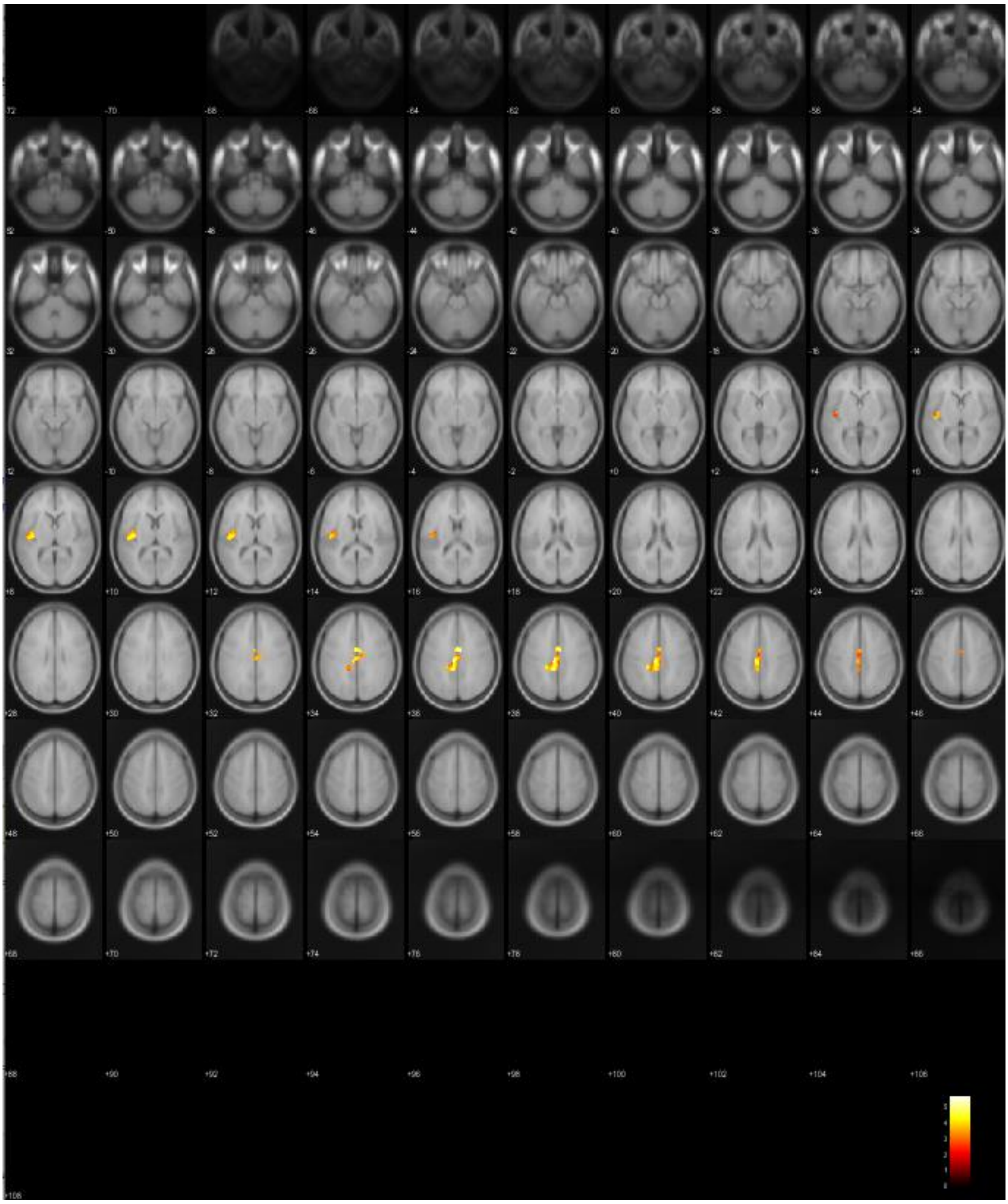

GLM#2 excluding relative forage value- Negative association for harvest value

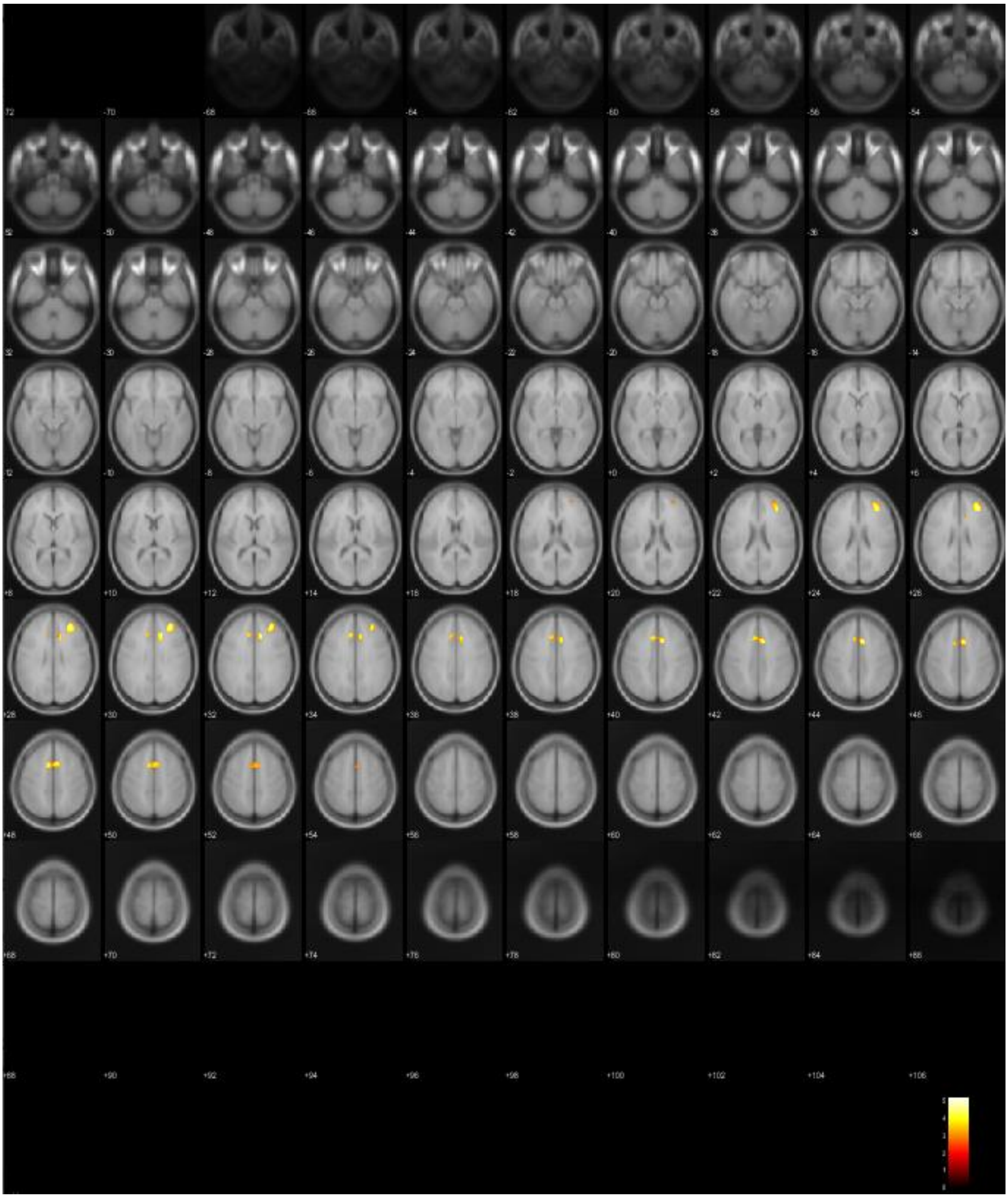

GLM#3 excluding harvest value- Negative association of patch-switching threshold

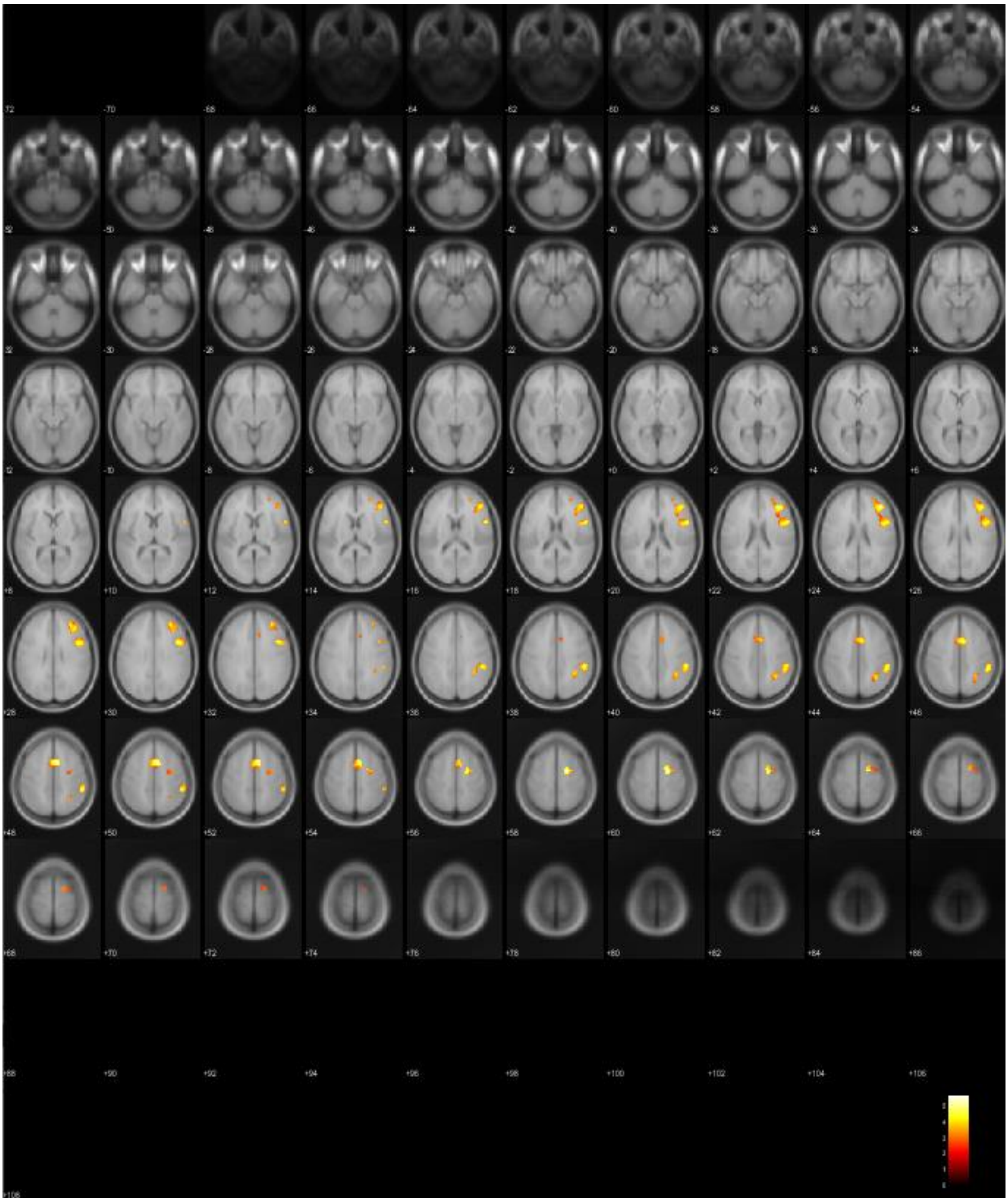

GLM#3 excluding harvest value- Positive association of relative forage value

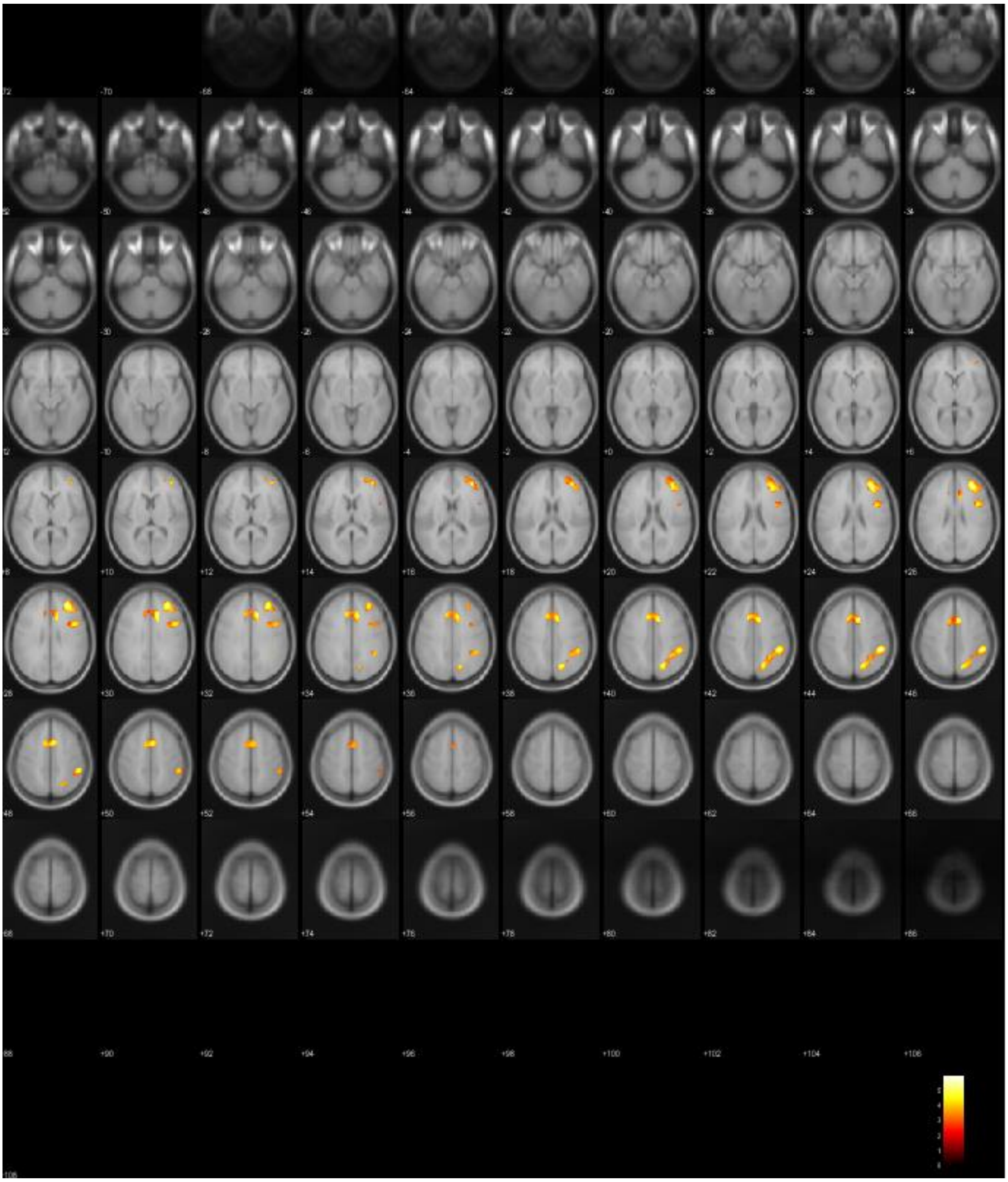

Supplement: SM_bhad088 [file sm_bhad088.pdf]
